# The temporal profile of activity-dependent presynaptic phospho-signalling reveals long-lasting patterns of poststimulus regulation

**DOI:** 10.1371/journal.pbio.3000170

**Published:** 2019-03-01

**Authors:** Kasper Engholm-Keller, Ashley J. Waardenberg, Johannes A. Müller, Jesse R. Wark, Rowena N. Fernando, Jonathan W. Arthur, Phillip J. Robinson, Dirk Dietrich, Susanne Schoch, Mark E. Graham

**Affiliations:** 1 Synapse Proteomics, Children’s Medical Research Institute, The University of Sydney, Westmead, Australia; 2 Department of Biochemistry and Molecular Biology, University of Southern Denmark, Odense, Denmark; 3 Cell Signalling Unit, Children’s Medical Research Institute, The University of Sydney, Westmead, Australia; 4 Bioinformatics Unit, Children’s Medical Research Institute, The University of Sydney, Westmead, Australia; 5 Genome Biology Unit, European Molecular Biology Laboratory, Heidelberg, Germany; 6 Institute of Neuropathology, University of Bonn Medical Center, Bonn, Germany; 7 Department of Neurosurgery, University of Bonn Medical Center, Bonn, Germany; Stanford University School of Medicine, UNITED STATES

## Abstract

Depolarization of presynaptic terminals stimulates calcium influx, which evokes neurotransmitter release and activates phosphorylation-based signalling. Here, we present the first global temporal profile of presynaptic activity-dependent phospho-signalling, which includes two KCl stimulation levels and analysis of the poststimulus period. We profiled 1,917 regulated phosphopeptides and bioinformatically identified six temporal patterns of co-regulated proteins. The presynaptic proteins with large changes in phospho-status were again prominently regulated in the analysis of 7,070 activity-dependent phosphopeptides from KCl-stimulated cultured hippocampal neurons. Active zone scaffold proteins showed a high level of activity-dependent phospho-regulation that far exceeded the response from postsynaptic density scaffold proteins. Accordingly, bassoon was identified as the major target of neuronal phospho-signalling. We developed a probabilistic computational method, KinSwing, which matched protein kinase substrate motifs to regulated phosphorylation sites to reveal underlying protein kinase activity. This approach allowed us to link protein kinases to profiles of co-regulated presynaptic protein networks. Ca^2+^- and calmodulin-dependent protein kinase IIα (CaMKIIα) responded rapidly, scaled with stimulus strength, and had long-lasting activity. Mitogen-activated protein kinase (MAPK)/extracellular signal–regulated kinase (ERK) was the main protein kinase predicted to control a distinct and significant pattern of poststimulus up-regulation of phosphorylation. This work provides a unique resource of activity-dependent phosphorylation sites of synaptosomes and neurons, the vast majority of which have not been investigated with regard to their functional impact. This resource will enable detailed characterization of the phospho-regulated mechanisms impacting the plasticity of neurotransmitter release.

## Abstract 2

Analysis of activity-dependent phosphorylation-based signalling in synaptosomes revealed six patterns of long-lasting presynaptic regulation from 1,917 phosphopeptides. The authors identified patterns most likely to be regulated by CamKII and MAPK/ERK and showed the active zone scaffold protein bassoon to be a major signalling target.

## Author summary

Neurobiological processes are altered by linking neuronal activity to regulated changes in protein phosphorylation levels that influence protein function. Although some of the major targets of activity-dependent phospho-signalling have been identified, a large number of substrates remain unknown. Here, we have screened systematically for these substrates and extended the list from hundreds to thousands of phosphorylation sites, thereby providing a new depth of understanding. We monitored phospho-signalling for 15 min after the stimulation, which to our knowledge had not been attempted at a large scale. We focused on presynaptic protein substrates of phospho-signalling by isolating the presynaptic terminal. We also stimulated hippocampal neurons but did not monitor the poststimulus. Although the phospho-signalling is immensely complex, the findings could be simplified through data exploration. We identified distinct patterns of presynaptic phospho-regulation across the time course that may constitute co-regulated protein networks. In addition, we found a subset of proteins that had many more phosphorylation sites than the average and high-magnitude responses, implying major signalling or functional roles for these proteins. We also determined the likely protein kinases with the strongest responses to the stimulus at different times using KinSwing, a computational tool that we developed. This resource reveals a new depth of activity-dependent phospho-signalling and identifies major signalling targets, major protein kinases, and co-regulated phosphoprotein networks.

## Introduction

Depolarization of the presynaptic plasma membrane stimulates the opening of voltage-gated Ca^2+^ channels, and Ca^2+^ rapidly enters at the active zone. Increased Ca^2+^ triggers neurotransmitter release by binding to Ca^2+^ sensors such as synaptotagmin 1, which facilitates fast synchronous fusion of docked synaptic vesicles [1–3]. Docking and priming of synaptic vesicles, as well as Ca^2+^ channel clustering [4, 5], are coordinated by scaffold proteins at the active zone. Ca^2+^ channels are tethered in proximity to synaptic vesicles directly by Rab3-interacting molecules (RIMs) [6, 7] and/or by a complex including bassoon and RIM-binding protein [8]. As such, the protein composition of the active zone scaffold not only is important for vesicle release but also exerts influence on release probability and presynaptic homeostatic plasticity [9].

The influx of Ca^2+^ following depolarization also stimulates phosphorylation-based signalling. Phosphorylation and dephosphorylation of presynaptic proteins at the active zone are strongly coupled to Ca^2+^ influx in the vicinity of Ca^2+^ channels, rather than the cytosolic Ca^2+^ concentration [10]. Ca^2+^ binds calmodulin, and this complex activates downstream phospho-signalling pathways that directly affect release properties. For example, Ca^2+^- and calmodulin-dependent protein kinase II (CaMKII) phosphorylation of synapsin 1 has a role in changing the availability of synaptic vesicles for release [11]. Ca^2+^/calmodulin also activates the phosphatase calcineurin (protein phosphatase 2B) and downstream phosphatases. Three synapsin 1 sites—S62, S67, and S549—are dephosphorylated by calcineurin and may contribute to increased availability of synaptic vesicles [12]. Calcineurin has also been shown to directly regulate synaptic vesicle endocytosis [13, 14]. The main protein kinases mediating presynaptic phospho-signalling and plasticity are protein kinase C (PKC) [15, 16], protein kinase A (PKA) [9], cyclin-dependent kinase 5 (CDK5) [17], and extracellular signal–regulated kinase 1/2 (ERK)/mitogen-activated protein kinase 1/3 (MAPK1/3) [18]. However, the identity of the presumably large number of substrates, the timing and interdependence of protein kinase activation or inactivation, and mechanistic consequences remain largely unresolved, as the complex activity-driven network of presynaptic kinases has previously not been explored systematically, to our knowledge.

There are few global studies of activity-dependent neuronal phospho-signalling. The postsynaptic density has been enriched to reveal postsynaptic signalling [19, 20]. Here, we use a subcellular compartment enrichment strategy to focus on presynaptic proteins. Sucrose homogenates of brain tissue produce a particulate fraction that contains both pre- and postsynaptic components but is primarily enriched in isolated presynaptic terminals, i.e., synaptosomes [21, 22]. Synaptosomes contain synaptic vesicles [22], are metabolically active, generate adenosine triphosphate [23], and maintain a membrane potential [24] and calcium homeostasis [25]. Thus, synaptosomes are a highly functional model for studying presynaptic phospho-signalling in isolation. Depolarization of synaptosomes with KCl stimulates concentration-dependent Ca^2+^ influx [25, 26]. A subset of the targets of presynaptic phospho-signalling, which are dependent on the initial Ca^2+^ influx, has been identified [27]. However, the broader and functionally important question of how synaptic phospho-signalling develops following a period of neuronal activity remains to be addressed. The poststimulus period has only been examined with very limited scope for a few well-characterized phosphorylation sites in synapsin 1 and MAPK [28, 29]. Such transient and persistent changes in the presynaptic phosphoproteome following activity are of crucial importance, as they are likely tightly linked to several forms of synaptic plasticity, which ensure that activity in neuronal networks remains within physiological limits and provide the basis for information storage, learning, and memory.

Here, we used quantitative phosphoproteomics to extensively define the targets and regulators of presynaptic phospho-signalling by examining both the stimulus and poststimulus periods. Exploration of the data revealed distinct temporal patterns of regulation. The relevance of our data is further underscored by the finding that cultured hippocampal neurons exhibited a similar activity-dependent response and confirmed the identity of several predominantly presynaptic phospho-signalling integrator proteins. A new computational method, KinSwing, was used to determine the profile of relative protein kinase activity, based on protein kinase substrate prediction. The inferred protein kinase activity was matched to the patterns of presynaptic phospho-regulation, revealing that a poststimulus up-regulation of phosphorylation was likely to be substantially mediated by MAPK/ERK. Proteins regulating vesicle release were prominent substrates of poststimulus up-regulation of phosphorylation.

## Results

### Phosphoproteomic analysis of depolarized and repolarized synaptosomes and depolarized hippocampal neurons

**Fig 1:**
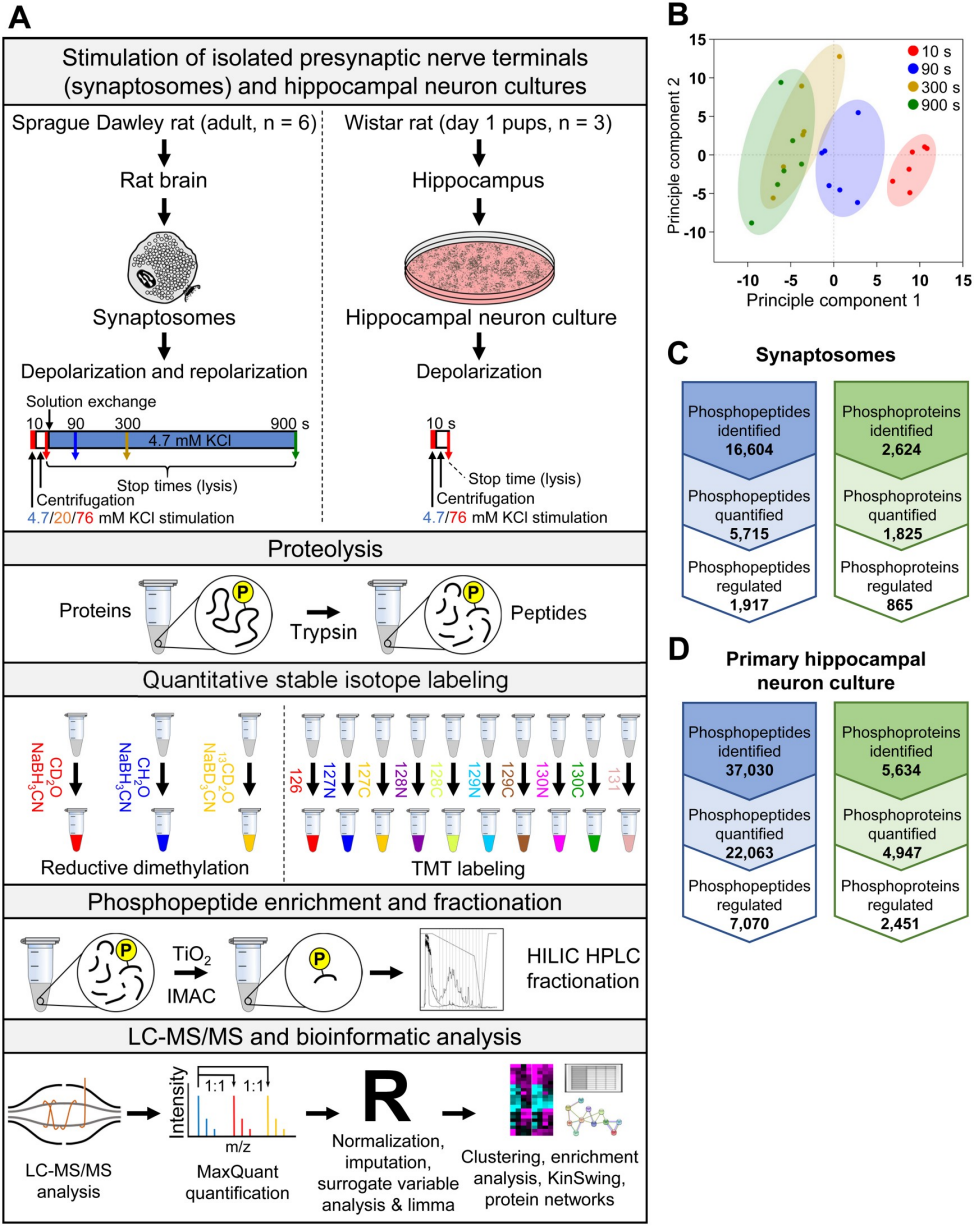
Quantitative analysis of activity-dependent phospho-signalling in synaptosomes and hippocampal neurons. (A) The experimental workflow started with either synaptosomes from the whole brain of adult rats or cultured primary hippocampal neurons. Shown is a schematic of the stimulation protocol for synaptosomes and neurons and the time points examined. Only the synaptosomes were repolarized by including a centrifugation and solution exchange step and were studied for the temporal evolution of phospho-signalling. The lysed samples were digested, enriched for phosphopeptides, analyzed by quantitative phosphoproteomics, and processed using a bioinformatics approach that included determination of inferred protein kinase activity using KinSwing. (B) Principal component analysis of the processed time course data for 76 mM KCl stimulation. All six biological replicates for each time point are shown in the same colour. Counts of the phosphopeptides and phosphoproteins identified are shown for (C) synaptosomes and (D) primary hippocampal neurons. The counts were calculated with and without filtering for phosphorylation site localization and quantification confidence. The phosphopeptide data are available in S1 Table. The synaptosome data are the result of six independent experiments for each stimulation condition (20 mM and 76 mM KCl). The cultured hippocampal neuron data are from three independent experiments. HILIC, hydrophilic interaction liquid chromatography; HPLC, high-performance liquid chromatography; IMAC, immobilized metal affinity chromatography; LC-MS/MS, liquid chromatography–tandem mass spectrometry; TMT, tandem mass tag.

Synaptosomes were stimulated with 20 mM KCl or 76 mM KCl (half of total monovalent salt)—or mock treated by keeping 4.7 mM KCl—for 10 s and subsequently returned to 4.7 mM KCl to enable repolarization. We confirmed the acute nature of this stimulation protocol by showing that the depolarization only acted during the 10-s period (S1 Fig, S1 Data). The synaptosomes were lysed at specific times to monitor changes to the phosphoproteome—i.e., at the end of the depolarization (10 s) and at three poststimulus time points (90, 300, and 900 s) (Fig 1A). Changes in phosphorylation levels relative to the mock stimulation over time were determined using a global quantitative phosphoproteomics workflow [30] (Fig 1A and Materials and methods).

The synaptosome data were processed and interrogated using a robust bioinformatics approach. Only high-confidence phosphopeptides with a probability score ≥ 0.75 for phosphorylation site assignment detected in at least three of the six biological replicates were used for further analyses. Our statistical workflow (Fig 1A and Materials and methods) involved normalization, missing value imputation, and correction for nonbiological sources of variation, as described [31, 32]. Unsupervised principle component analysis (PCA) of all phosphopeptides separated the time points across the first principle component, indicating that phosphorylation of peptides was largely occurring in a time-dependent manner (Fig 1B). A total of 5,715 unique phosphopeptides were quantified in the intersection of 20 and 76 mM KCl stimulation experiments from 1,825 proteins (not counting isoforms and multiprotein identifications) (Fig 1C and S1 Table). A total of 1,917 phosphopeptides were significantly up-/down-regulated over time and detected in both the 20 mM and 76 mM KCl conditions. Significance across time was determined using a moderated F-statistic adjusted for multiple hypothesis testing. *P* < 0.05 was required for inclusion in the set of 1,917 phosphopeptides.

The phosphoproteome was perturbed more by 76 mM KCl stimulation, when compared to 20 mM KCl, and at earlier time points. At 10 s, there were approximately double the number of significant changes resulting from 76 mM KCl stimulation compared to 20 mM KCl (S2A–S2C Fig; a moderated t-statistic adjusted for multiple hypothesis testing was used to determine the significance of single time points, *P* < 0.05). This supports the view of a graded response of the presynaptic phosphoproteome to different levels of stimulation. At 300 and 900 s, significant phospho-signalling persisted, but the difference in number of significantly regulated phosphorylation sites between 20 mM and 76 mM KCl stimulation was smaller (S2A–S2C Fig). Thus, perturbation of phospho-signalling was found to be long-lasting as a consequence of acute stimulation.

To provide a resource of broad utility, we also stimulated cultured hippocampal neurons with 76 mM KCl for 10 s and quantified changes in phospho-signalling using our bioinformatics approach (Fig 1A**)**. This resulted in the confident identification of 22,063 unique phosphopeptides (Fig 1D and S1 Table), of which 7,070 were significantly regulated by the stimulation (moderated t-statistic adjusted for multiple hypothesis testing, *P* < 0.05). Only 4.6% of these activity-dependent sites have a known function or regulatory role, and only four of the top 100 largest magnitude significant changes have been explored up to now (PhosphoSitePlus, [33]). A similarly small fraction of activity-independent phosphorylation sites (4.8%) identified have a known function or regulatory role. This indicates a very large gap in the knowledge of signalling mechanisms dependent on neuronal activity.

**Fig 2:**
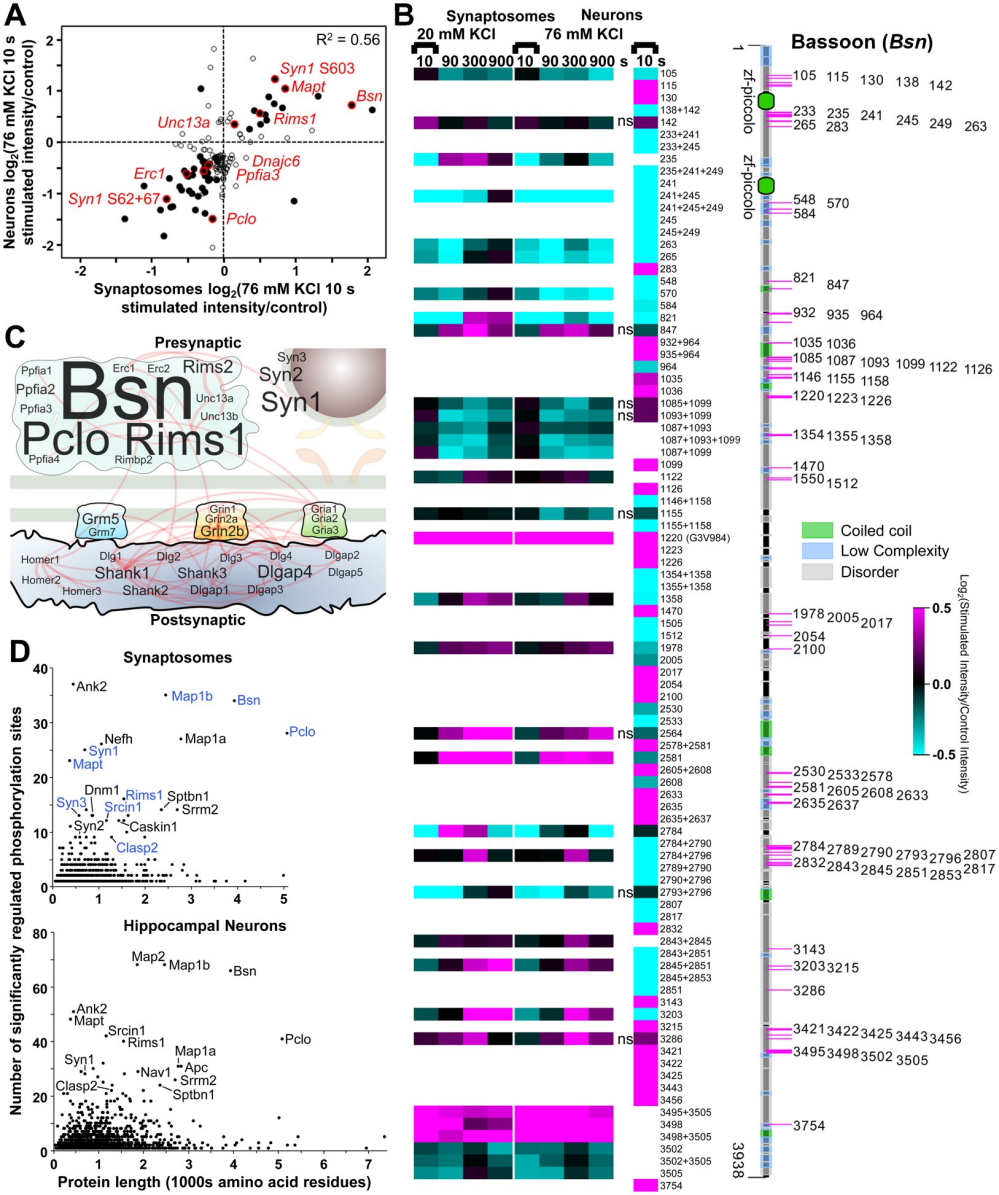
Comparison of activity-dependent changes in synaptosomes and cultured neurons identifies bassoon as a major target of phospho-signalling. (A) Plot of log_2_(76 mM KCl 10-s-stimulated intensity/control intensity) for selected phosphopeptides from cultured hippocampal neurons versus whole-brain synaptosomes. The phosphopeptides compared were required to be from well-established presynaptic proteins. That is, they were exclusively active zone scaffold proteins or synaptic vesicle–associated proteins. Thus, by default, all postsynaptic and ubiquitous proteins were excluded. Solid circles are phosphopeptides significantly regulated in both types of samples. Open circles are phosphopeptides significantly regulated in at least one sample. Selected solid circles have a red outline and are labelled with a gene name. Two specific synapsin 1 phosphorylation sites are featured. Underlying data for this figure can be found in S1 Data. (B) Heat map of bassoon log_2_(stimulated intensity/control intensity) values across time for significantly regulated phosphopeptides from synaptosomes and hippocampal neurons using the indicated colour scale. Note that some log_2_ fold changes are outside of the colour scale range. The chosen scale was intended to provide greater emphasis for low-moderate fold changes throughout this work, even though some signals will be saturated. See (A) for an example of the data shown on a larger log_2_ scale. The bassoon domain structure of UniProt accession O88778, obtained from Pfam [34], is shown with accurate phosphorylation site positions. Synaptosome data were required to have significant up-/down-regulation at one or more time points. For hippocampal neurons, “ns” means not significantly regulated. Phosphorylation site positions are from bassoon accessions O8878 and G3V984 (see S1 Table). (C) A protein localization and interaction network for selected protein components of the active zone scaffold, synaptic vesicles, postsynaptic density, and glutamate receptor proteins. The log_2_ intensities for phosphopeptides after 10 s of 76 mM KCl stimulation of hippocampal cultured neurons were summed for each protein. This value was used to scale the letter size of the gene name in the protein network. Experimentally verified protein interactions (STRING) are shown as red edges. (D) Graphs of the number of significantly regulated phosphorylation sites against the protein length (amino acid residues) for synaptosomes (upper) and hippocampal neurons (lower). Proteins with relative high numbers of regulated phosphorylation sites are labelled by their gene name. Proteins proposed to be presynaptic signal integrators have blue labels. There was no linear correlation between the parameters in either graph of synaptosome (R^2^ = 0.06) or neuronal data (R^2^ = 0.04). The synaptosome data are the result of six independent experiments for each stimulation condition (20 mM and 76 mM KCl). The cultured hippocampal neuron data are from three independent experiments. Underlying data for this figure can be found in S1 Data.

A total of 3,549 phosphopeptides were identified in both neurons and synaptosomes (intersection). An intersection of 195 phosphopeptides from 123 proteins were significantly regulated after 10 s of 76 mM KCl stimulation in synaptosomes and neurons. These phosphopeptide signals were well correlated (R^2^ = 0.51, S2D Fig, S1 Data). To avoid comparison of phosphoproteins that occur in multiple subneuronal compartments, we focused on active zone scaffold and synaptic vesicle–associated proteins, which are highly specific to the nerve terminal and key components of the release machinery (listed in S1 Table), thereby excluding postsynaptic proteins by default. This presynaptic-focused correlation analysis is shown in Fig 2A (S1 Data). Active zone scaffold proteins (bassoon; piccolo; RIM1; liprin-α3; protein rich in E, L, K, and S 1 [ELKS1]; corresponding to gene names: *Bsn*, *Pclo*, *Rims1*, *Ppfia3*, and *Erc1*), synaptic vesicle endocytosis-specific clathrin uncoating protein auxilin 1 (*Dnajc6*), and presynaptic-specific cytoskeletal organizing protein tau (*Mapt*) were among those proteins with highly correlated activity-dependent phospho-signalling (R^2^ = 0.56, Fig 2A, S1 Data). The high correlation between the activity-induced phospho-signalling in synaptosomes and cultured hippocampal neurons indicates that signalling pathways in synaptosomes were well preserved.

Eight of the top 100 largest changes from hippocampal neurons were sites from bassoon (Fig 2B). Bassoon appears to be the major target of neuronal activity-dependent phospho-signalling and is targeted for phosphorylation and dephosphorylation along its entire lengthy sequence (Fig 2B). In contrast, the postsynaptic density proteins and glutamate receptors had a relatively modest response to stimulation (Fig 2C). A formal comparison of the number of significantly regulated phosphorylation sites against protein length revealed that bassoon did indeed have one of the highest numbers of regulated phosphorylation sites per amino acid residue. There was no correlation between number of regulated phosphorylation sites and length (Fig 2D, S1 Data). The same lack of correlation was observed for the regulated phosphorylation sites from hippocampal neurons (Fig 2D). Presynaptic substrates, such as RIM1 and microtubule-associated protein 1B (MAP1B; gene names: *Rims1* and *Map1b*) featured among the high-magnitude changes (S1 Table and S1 Data), indicating that activity-dependent phospho-signalling has a pronounced effect on a subset of presynaptic substrates.

### Validation of activity-dependent phospho-signalling

**Fig 3:**
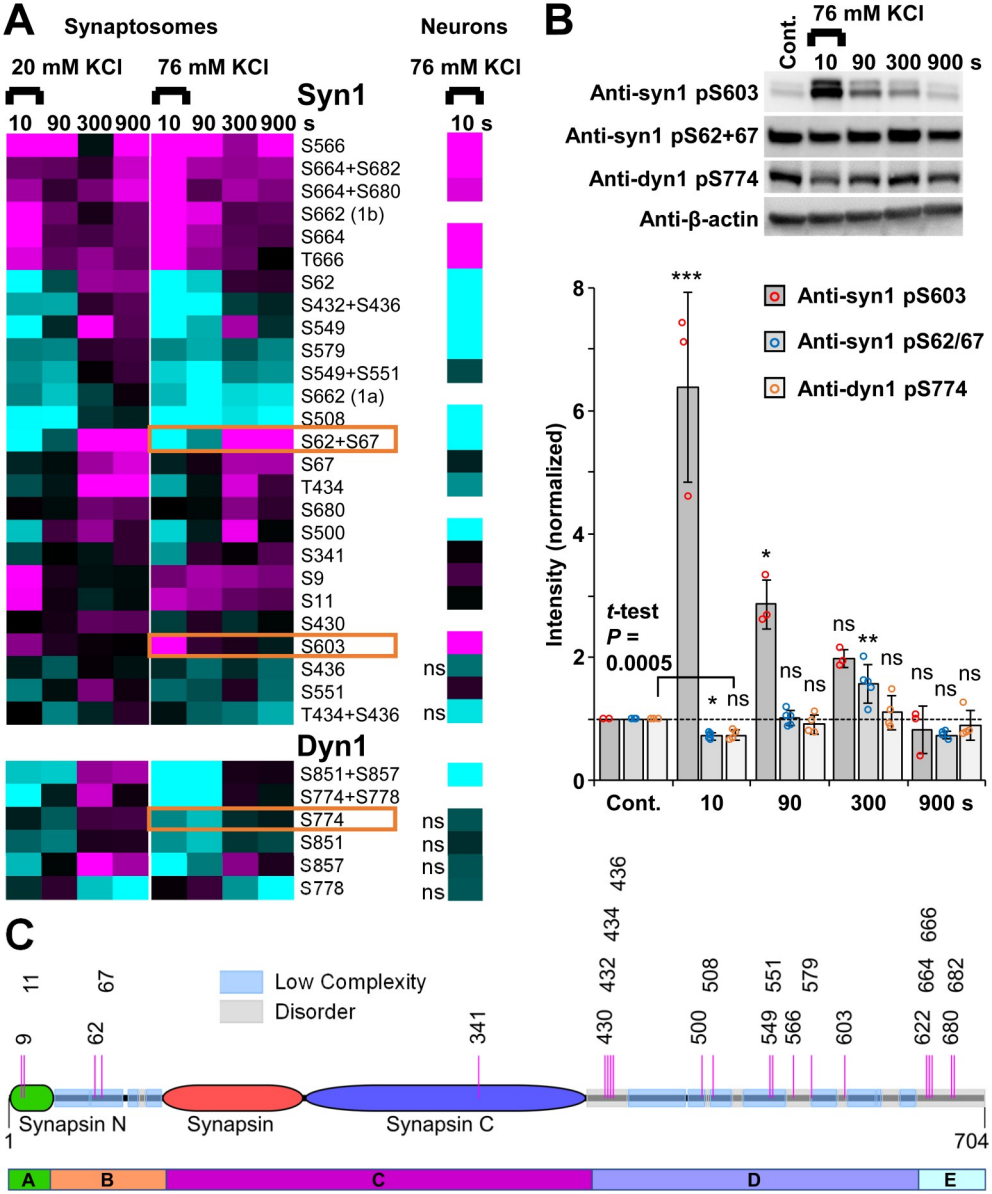
Validation of stimulus and poststimulus phospho-signalling. (A) Heat map of significantly regulated phosphorylation sites for syn1 (upper) and dyn1 (lower) from 20 mM and 76 mM KCl stimulated synaptosomes (left, *n* = 6) and a single 10-s time point of 76 mM KCl stimulated hippocampal neurons (right, “ns” means not significantly regulated, *n* = 3). The log_2_(stimulated intensity/control intensity) is shown using the same colour scale as Fig 2B. Note: phospho-S662 in syn1 was detected with differential regulation from 1a and 1b isoforms. The synaptosome data are the result of six independent experiments for each stimulation condition (20 mM and 76 mM KCl). The cultured hippocampal neuron data are from three independent experiments. (B) Representative western blots of syn1-pS603, syn1-pS62+S67, dyn1-pS774, and β-actin loading control for 76 mM KCl depolarized and repolarized synaptosomes. Bar graphs of the densitometry of the western blots, after correction for loading, are shown (below). The intensities were normalized to the control (“Cont.”)/mock stimulation. The bar graphs show the mean and standard deviation of 3 (syn1 pS603), 4 (dyn1 S774), or 5 (syn1 S62+S67) independent experiments. Statistical significance was determined by one-way analysis of variance with Dunnett’s post hoc test; *P < 0.05; **P < 0.01; ***P < 0.001; in (B), “ns” means “not significant”. *P* = 0.0005 using Student *t* test to compare dyn1-pS774 intensity at 10 s versus control and *P* = 0.17 when adjusted for multiple comparisons (time points). The heat map rows in (A) for the sites examined in (B) are boxed. Underlying data for this figure can be found in S1 Data. (C) Domain structure of Syn1 using A-E domain naming [39] and Pfam evolutionarily conserved domains [34] with the activity-dependent phosphorylation site positions indicated. dyn1, dynamin 1; syn1, synapsin 1.

The vesicle-tethering protein synapsin 1 is the only presynaptic protein that has been substantially examined during the poststimulus period [28]. We identified 26 significantly regulated synapsin 1 phosphopeptides from synaptosomes. Synapsin 1 S566, a CaMKIIα substrate site from in vitro studies [35], was the most up-regulated site at 10 s (Fig 3A). Another CaMKIIα substrate and activity-dependent site, S603 [12], was also up-regulated at 10 s (Fig 3A), and this was confirmed by western blot (Fig 3B, S1 Data) using 76 mM KCl stimulation. Phosphorylation of these D domain sites is expected to promote the dispersion of the recently identified synapsin liquid phase and associated vesicles [36]. Phosphorylation was generally up-regulated for the vesicle binding A domain and the E domain of synapsin 1. The phospho-regulation patterns were complex for domains B and D that contain evolutionarily conserved low complexity and disordered sequences, as identified by the Pfam (protein families) database [34] (Fig 3C). In agreement with a previous report [28], we observed bidirectional regulation of S549 and S62+S67 in our synaptosome data for both 20 and 76 mM KCl stimulations (Fig 3A). Another well-studied presynaptic substrate, dynamin 1 [27, 37, 38], was down-regulated at major activity-dependent phosphosites S774 and S778 at 10 s (Fig 3A and 3B). Thus, our synaptosome phosphoproteomics data reproduce known stimulus and poststimulus phospho-specific profiles, verifying the validity of our stimulation and repolarization paradigm.

Furthermore, the majority of synapsin 1 phosphorylation sites had the same direction of regulation after 76 mM KCl stimulation in both synaptosomes and hippocampal neurons (16 of 19 significantly regulated at 10 s, Fig 3A). Western blotting confirmed up-regulated phosphorylation at S603 and down-regulated S62+S67 phosphorylation (Fig 3A, boxed), which were highly correlated phosphorylation sites in Fig 2A (red circles).

### Data clustering and enrichment analysis

**Fig 4:**
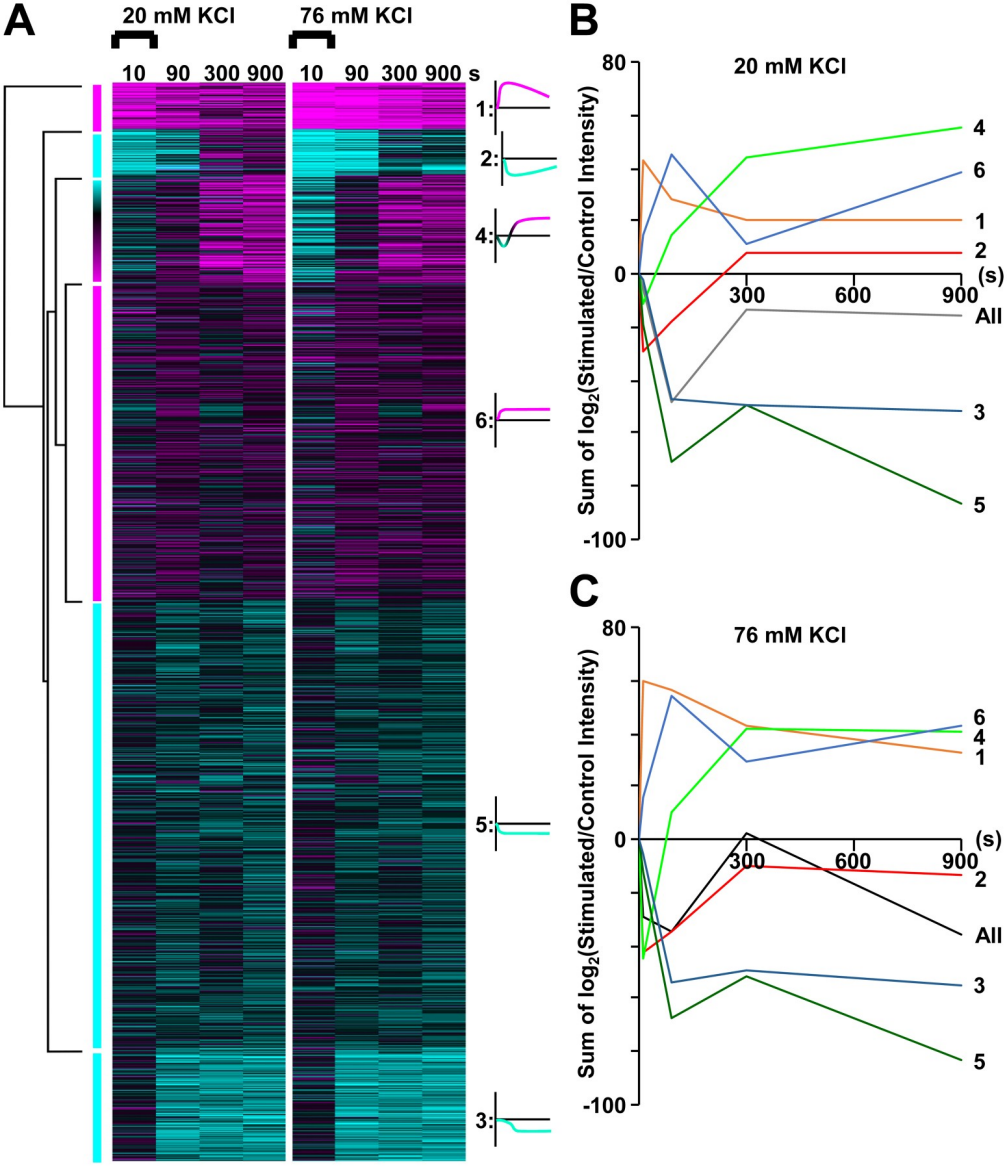
Clustering of temporally profiled phosphopeptides from synaptosomes stimulated at two KCl concentrations. (A) Heat map of k-means clustering of all 1,917 phosphopeptides for both 20 and 76 mM KCl experiments with at least one significant change at any time point. Log_2_(stimulated intensity/control intensity) is shown using the same colour scale as in Fig 2B. Dendrogram shown on the left. Representative line graph of each cluster shown on the right. (B-C) Graphs accurately depicting the sum of log2(stimulated intensity/control intensity) versus time for each of the six clusters are shown for 20 mM (B) and 76 mM (C) KCl stimulations. The data are the result of six independent experiments for each stimulation condition (20 mM and 76 mM KCl). Underlying data for this figure can be found in S1 Data.

To determine which groups of proteins and/or biological processes had similar activity-dependent temporal phospho-signalling, the 1,917 significantly regulated phosphopeptides from synaptosomes were k-means clustered across time for both concentrations of KCl (Fig 4A and S1 Table). Phospho-regulated postsynaptic proteins were not filtered from the data prior to the clustering, or for any analysis in this work, except where stated (Fig 2A). We rely on the knowledge that, although small intact postsynaptic compartments are present in synaptosome preparations, the predominant component is the isolated presynaptic terminal [22]. Six clusters were determined as optimal for enrichment analysis (see S3A and S3B Fig, S1 Data). Clusters were summarized as (1) high-magnitude up-regulated, (2) high-magnitude down-regulated, (3) poststimulus down-regulated, (4) poststimulus up-regulated, (5) low-magnitude down-regulated, and (6) low-magnitude up-regulated, represented by a small stylized line graph adjacent to the heat map in Fig 4A. These line graphs are representative of the overall 20 mM and 76 mM KCl stimulated patterns. Fig 4B and 4C (S1 Data) show the sum of log_2_ (stimulated intensity/control intensity) for each cluster versus time for the 20 and 76 mM KCl stimulations and are provided to allow a comparison of the cluster trends using accurate line graphs.

Cluster 4 phosphorylation was, overall, slightly down-regulated at 10 s and highly up-regulated at 300–900 s after 20 mM KCl (Fig 4A–4C). After 76 mM KCl, the down-regulation at 10 s was greater in magnitude, and there was less up-regulation at 300–900 s. Cluster 2 phospho-regulation also exhibited an overall slight bidirectionality; a change in direction occurred for some phosphopeptides from 90 to 300 s after 20 mM KCl (Fig 4A–4C). The pattern changed to down-regulation at all time points after 76 mM KCl. Cluster 1 and 2 had high-magnitude initial regulation that continued into the poststimulus, and the regulation extended further after 76 mM KCl (Fig 4A–4C). Cluster 1 up-regulation outlasted cluster 2 down-regulation for at least 10 min of the time course after 20 mM KCl (Fig 4A and 4B). Cluster 3 phosphorylation sites mainly failed to respond at 10 s but were down-regulated throughout the poststimulus, and this was independent of the strength of stimulus (Fig 4A–4C). This implies a temporally controlled signalling mechanism that specifically changes the poststimulus balance of phosphorylation and dephosphorylation independent of the level of Ca^2+^ influx. Cluster 5 was similarly disconnected from the stimulus strength in the poststimulus but at a relatively low level of down-regulation. Low-magnitude up-regulated cluster 6 had more phosphopeptides up-regulated at 300 s after 76 mM KCl (Fig 4A–4C). Thus, clustering enabled the identification of six specific patterns of phosphopeptide regulation, including high-magnitude up-regulation > 5 min post stimulus, and some were highly influenced by the stimulus strength.

**Fig 5:**
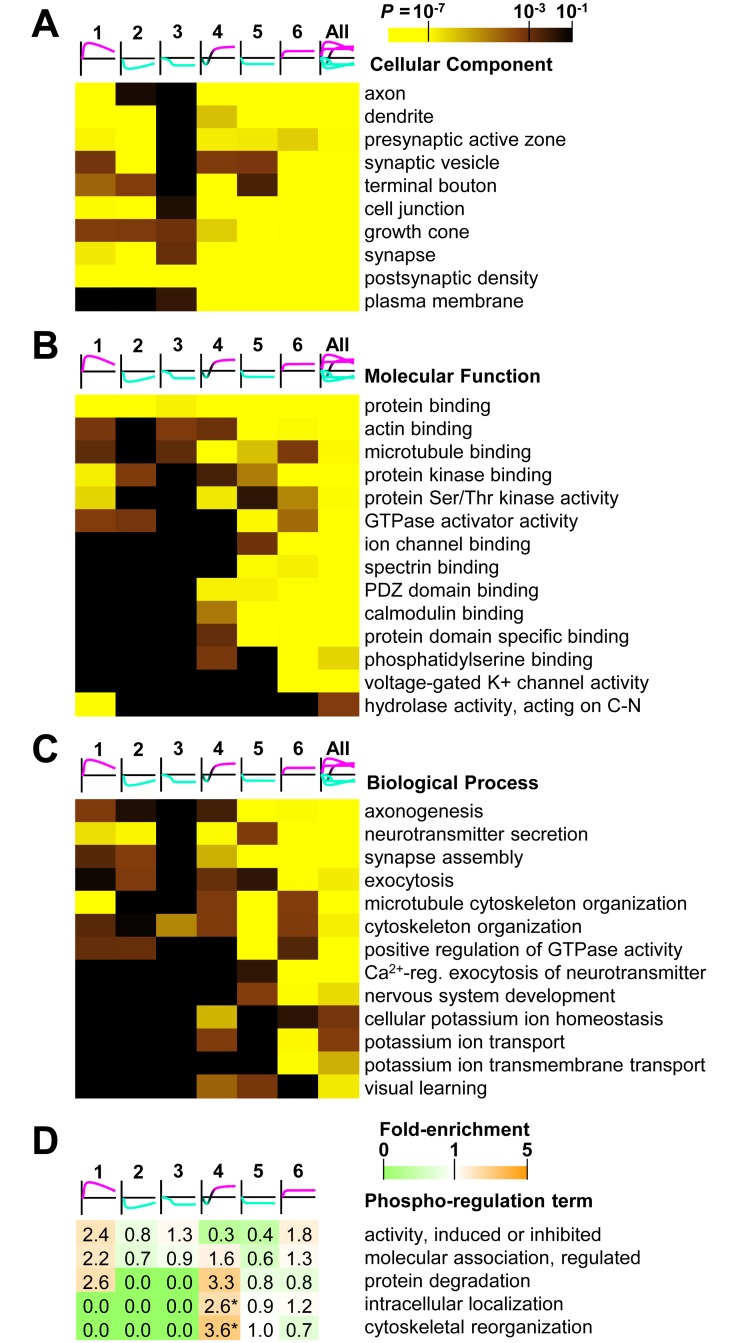
Enrichment of gene ontology terms for clusters of temporally regulated phosphopeptides. Heat maps are shown for the probability of (A) cellular component, (B) molecular function, and (C) biological process term enrichment for each cluster, using the indicated colour scale. Underlying data for this figure can be found in S1 Data. (D) Heat map and fold enrichment of phosphorylation site regulation ontology terms from PhosphoSitePlus [33] for each cluster (*P < 0.05). GTPase, guanosine triphosphatase; reg., regulated; Ser/Thr, serine/threonine.

Each cluster was subsequently investigated for gene ontology enrichment. The cellular component term “presynaptic active zone” was most enriched in cluster 2, followed by clusters 1, 4, and 5. There was a lack of enrichment in cluster 3 (Fig 5A, S1 Data). Clusters showing enrichment contained active zone proteins such as bassoon, piccolo, RIM1, liprin-α3, ELKS1, and ELKS2 [5]. High-magnitude down-regulated cluster 2 was also enriched with the “synaptic vesicle” term (Fig 5A) and contained synapsin 1 and endocytic proteins known to be substrates of calcineurin [12, 13]. Multiple members of the microtubule-binding collapsin response mediator protein family were specifically enriched in cluster 1, resulting in inclusion of the “hydrolase activity, acting on carbon-nitrogen (but not peptide) bonds” molecular function term in Fig 5B (S1 Data). The “protein serine/threonine kinase activity” molecular function term was enriched for clusters 1 and 4. The “protein kinase binding” term was enriched for up-regulated clusters 1 and 6 and somewhat enriched for down-regulated clusters 2 and 5 (Fig 5B). Poststimulus up-regulated cluster 4 was enriched with the molecular function term “microtubule binding” (Fig 5B). Low-magnitude down-regulated cluster 5 was most enriched for “GTPase activator activity”. Low-magnitude up-regulated cluster 6 was the only cluster enriched for “voltage-gated potassium channel activity” (Fig 5B) and the biological process term “potassium ion transmembrane transport” (Fig 5C, S1 Data). The term “neurotransmitter secretion” was enriched for clusters 1, 2, 4, and 6. Clusters 2 and 6 were enriched for “exocytosis” (Fig 5C). Cluster 1 was enriched for the “microtubule cytoskeleton organization” term (Fig 5C). Cluster 3 lacked significant enrichment for most terms, except “cytoskeletal organization” (Fig 5C). In general, higher-magnitude clusters 1, 2, and 4 revealed phospho-signalling that targeted active zone scaffold components, protein kinases, the collapsin response mediator protein family, and vesicular and cytoskeletal-related proteins, which may influence neurotransmitter release, the synaptic vesicle cycle, and regulation of the cytoskeleton/microtubules.

The enriched gene ontology terms lacked cluster specificity (Fig 5A–5C). The enrichment analysis may have been undermined by proteins participating in multiple distinct signalling pathways. These proteins contained phosphorylation sites spread across multiple clusters. Piccolo, bassoon, and MAP1B (gene names: *Pclo*, *Bsn*, and *Map1b*) had phosphopeptides represented in all six clusters. Cytoplasmic linker–associated protein 2 (*Clasp2*) as well as synapsin 1 and 3 were represented in five of six clusters. The number of regulated phosphorylation sites for each protein was plotted against the number of clusters of which each protein was a member in S4A Fig. This enabled the identification of proteins highly represented in greater than four clusters. Further separation of proteins was achieved by using the largest magnitude change of log_2_ intensity as a multiplier of cluster number (S4B Fig, S1 Data). Tau (*Mapt*), SNAP25-interacting protein (*Srcin1*), and RIM1 (*Rims1*) were identified as proteins represented in four of six clusters, with relatively high-magnitude responses and numbers of significantly regulated phosphorylation sites (S4A and S4B Fig, S1 Data). In contrast, spectrin beta chain (273 kDa, *Sptbn1*) had 14 regulated phosphopeptide signals that were present in only two clusters, 13 of which were in lower-magnitude cluster 5 (S1 Table). As shown in Fig 2D (blue-coloured gene names), the same proteins we singled out for representation in multiple clusters (S5A–S5F Fig) had higher numbers of regulated sites per length but were not required to be large proteins. Representation in many clusters suggests participation in multiple phospho-signalling pathways, which can potentially be independently modulated and impact multiple downstream functions. These highly phospho-regulated proteins are important presynaptic signal integrators and indicate that presynaptic protein functions can be subject to diverse phospho-signalling pathways.

Since phosphorylation site functions do not relate directly to ontology terms at the gene level (Fig 5A–5C), we investigated ontology at the level of phosphorylation sites. Publicly available curated descriptions of the regulatory role of specific phosphorylation sites are available from PhosphoSitePlus [33]. We determined the enrichment of these phospho-regulation terms for each cluster (Fig 5D, see Materials and methods). Most clusters exhibited a different pattern of enrichment. However, the low number of phosphorylation sites associated with phospho-regulation terms (<5%, see S1 Table) limited the power of this analysis. Significant fold enrichment was determined in poststimulus up-regulated cluster 4 for “intracellular localization” and “cytoskeletal reorganization” terms (*P* < 0.05, Fig 5D). The process of cytoskeletal reorganization was collectively regulated by specific phosphorylation sites in tau (*Mapt*), phosphatidylinositol 4-phosphate 5-kinase type-1 γ (*Pip5k1c*), insulin receptor substrate p53 (IRSp53; *Baiap2*), Septin 7 (*Sept7*), and exocyst complex component 7 (*Exoc7*) (S1 Table). Thus, poststimulus phospho-regulation of the cytoskeleton was a significant process identified in the pattern of cluster 4.

### Unbiased assessment of protein kinase contribution over time using KinSwing

The clusters of phosphopeptides in Fig 4A were first analyzed for protein kinase substrate motif enrichment using motif-x [40]. The CaMK protein kinase family RXXS motif was enriched for cluster 1 (S6A Fig). Proline-directed motifs, with proline in the +1 position relative to the phosphorylation site, were mainly enriched for down-regulated clusters. The PXSP motif associated with MAPK substrates [41] was enriched in cluster 4 (S6A Fig). These results indicate that particular families or classes of protein kinases may be associated with the clusters of regulated phosphorylation sites. However, this approach lacks the sophistication required to narrow the prediction to specific protein kinases.

**Fig 6:**
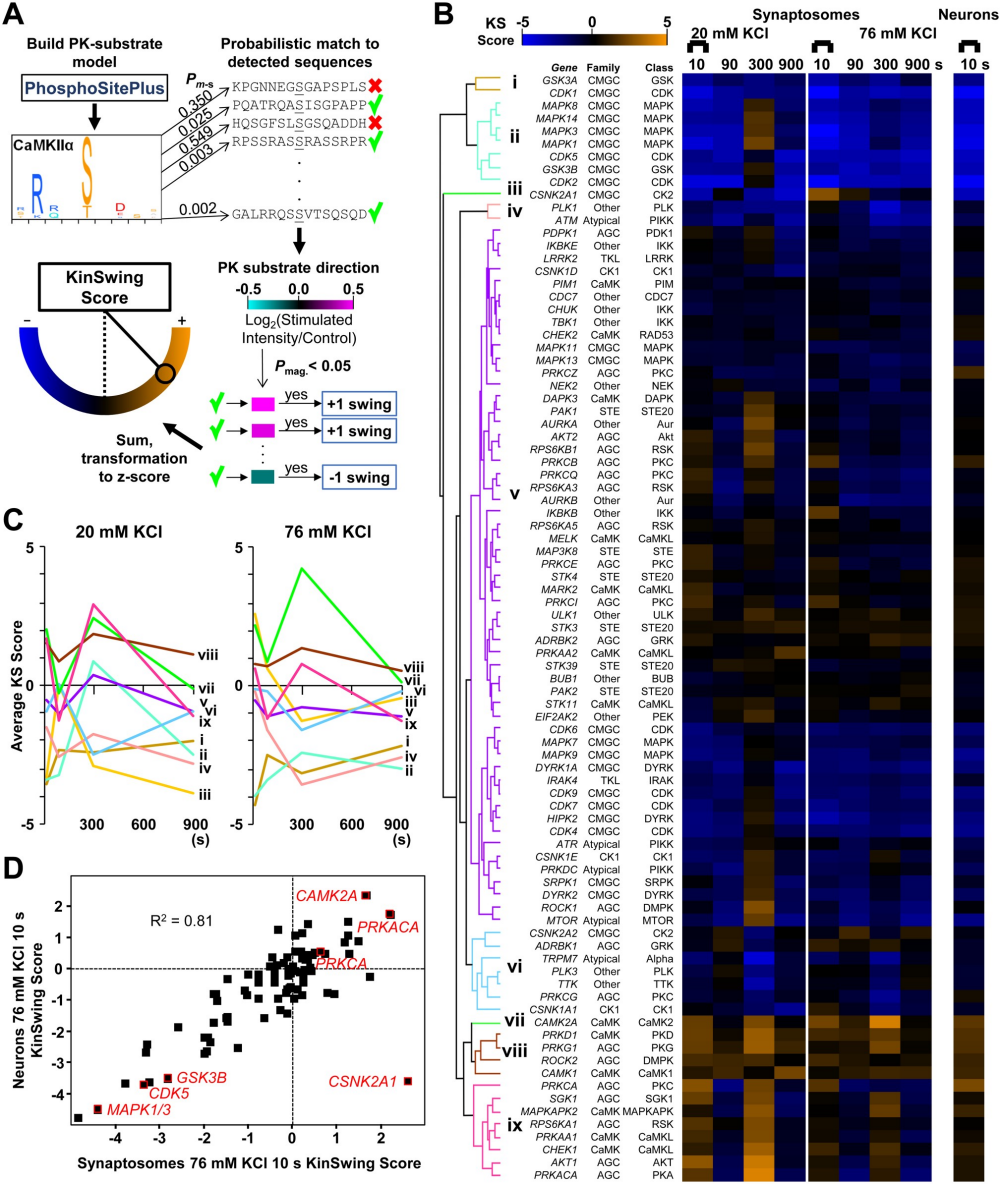
Determination of the inferred protein kinase activity across time using KinSwing. (A) Schematic of the process for generating KS scores. The human data contained in PhosphoSitePlus [33] were used to determine PK substrate motifs for each protein kinase (CaMKIIα shown as an example). The probability of a sequence match, *P*_m-s_, between the PK substrate motif and the centred sequence of each detected phosphorylation site was calculated. The magnitude of the significantly regulated phosphorylation sites (*P*_mag._ < 0.05) was used to assign positive or negative (+1/−1) swing. An overall swing was determined from the sum of swings for the group of probable PK substrates. The swing was transformed to a z-score (KS score) to allow for comparison across time points (see Materials and methods for a detailed description). The process was repeated for each condition and time point independently. (B) Left: Heat map of KS scores across time for 20 mM and 76 mM KCl for a filtered set of protein kinases arranged by hierarchical clustering and simplified to nine clusters (i to ix). The Ser/Thr PK families and classes are indicated. Right: Heat map of KS scores (meeting the criteria in Materials and methods) for cultured hippocampal neurons after 10 s of 76 mM KCl. (C) Graph of the average KS score versus time for each of the nine clusters for 20 mM and 76 mM KCl. Underlying data for this figure can be found in S1 Data. (D) Plot of KS scores after 10 s of 76 mM KCl for neurons versus synaptosomes (S1 Table). Selected protein kinases are shown in red. The synaptosome data are the result of six independent experiments for each stimulation condition (20 mM and 76 mM KCl). The cultured hippocampal neuron data are from three independent experiments. AGC, protein kinase A, G, and C; AKT, protein kinase B; CaMKIIα, Ca^2+^- and calmodulin-dependent protein kinase; CaMKL, CaMK-like; CDC7, cell division cycle 7-related; CDK, cyclin-dependent kinase; CGMC, CDK, GSK3, MAPK, and CLK; CK, casein kinase; DAPK, death-associated protein kinase; DMPK, myotonic dystrophy protein kinase; DYRK, dual-specificity tyrosine-regulated kinases; GRK, G protein–coupled receptor kinase; GSK, glycogen synthase kinase; IKK, I kappa B kinase; KS score, KinSwing score; LRRK, leucine-rich repeat kinase; MAPK, mitogen-activated protein kinase; MAPKAPK, MAPK-activated protein kinase; MTOR, mechanistic target of rapamycin; PDK1, pyruvate dehydrogenase kinase 1; PIKK, phosphatidylinositol 3-kinase-related kinase; PK, protein kinase; PLK, polo-like kinase; RSK, ribosomal S6 kinase 1; Ser/Thr, serine/threonine; STE, sterile; TKL, tyrosine kinase-like.

Ideally, we would identify and determine the contribution of the major protein kinases at specific time points. To achieve this, we developed a computational method named KinSwing. KinSwing is a statistical approach that integrates known protein kinase substrate motifs, probabilistic matching to substrate sequences, significance of phospho-regulation, and direction of phospho-regulation over time to infer the contribution of specific protein kinases to phospho-signalling (Fig 6A, see Materials and methods). KinSwing does not require the data to be clustered a priori. Instead, KinSwing determines protein kinase contribution at each time point, which can be subsequently used for clustering. First, position weight matrices, representing the frequency of occurrence for each amino acid residue, at and adjacent to the phosphorylation site, were determined for 355 protein kinases using experimentally verified substrate sequences described in the PhosphoSitePlus database [33]. The probability of a significant motif match for each phosphopeptide in our study was then determined and used to identify substrate sequences. The substrates that either significantly increased or decreased in phosphorylation, at each time point, were assigned a positive or negative “swing” (Fig 6A). An overall sum of “swings” was calculated, with consideration for the number of substrate sequence matches, such that the sum for each protein kinase was weighted and transformed into a z-score, enabling relative comparisons between protein kinases and across time (see Materials and methods). The z-score will henceforth be referred to as the KinSwing score (KS score).

KinSwing aims to predict the positive or negative inferred activity for a specific protein kinase. Eighty-seven KS score profiles were clustered (Fig 6B) after removing protein kinases based on consensus sequences with limited and highly variable scores (see Materials and methods and S1 Table). Clusters are represented as line graphs of averaged KS scores in Fig 6C (S1 Data). It is important to appreciate that protein kinase activity should be considered in light of opposing protein phosphatase activity. Since substrate motifs for protein phosphatases lack specificity relative to protein kinase substrates and protein kinases have a more diverse impact on cellular pathways [42], we take a protein kinase–centric view of activity. Also, KinSwing cannot resolve the activities of protein kinases and protein phosphatases at the subcellular level when applied to phosphoproteomics data from whole cells.

KS profiles allowed the identification of the major regulatory protein kinases and their patterns of regulation. CaMKIIα (*CAMK2A*) clustered alone (Fig 6B and 6C, cluster vii) and was the only protein kinase predicted to have increased activity after 76 mM KCl stimulation, relative to 20 mM KCl stimulation. Many protein kinases contain regulatory phosphorylation sites, which could be utilized to validate activity inferred by KinSwing. There were 23 and 68 significantly perturbed protein kinase regulatory sites identified from synaptosomes and hippocampal neurons, respectively (S1 Table, regulatory sites listed). The T286 autoactivation site and the T306 inhibitory site for CaMKIIα were up-regulated and down-regulated, respectively, at 10 s in hippocampal neurons but were not detected in synaptosomes using mass spectrometry (S7A Fig). Western blotting demonstrated that T286 was indeed significantly up-regulated at 10–300 s in synaptosomes (S7A Fig, S1 Data). In support of increased protein kinase activity, the CaMKIIα S275 putative autoactivation site [43] was significantly up-regulated at all time points in synaptosomes by mass spectrometry (S7A Fig).

CaMKIIα binds the postsynaptic density and to synaptic vesicles. In S7B Fig (S1 Data), we compared the regulatory patterns and probabilities of confident CaMKIIα, PKAα, and PKCα substrate motif matches for the pre- and postsynaptic substrates in Fig 2C. Disks large-associated protein 4 (*Dlgap4*) and SH3 and multiple ankyrin repeat domains protein 3 (*Shank3*) had the largest up-regulation of those predicted as CaMKIIα substrates by KinSwing (S7B Fig). Known CaMKIIα substrate glutamate receptor 1 S849 (S831 in mature protein, gene name: *Gria1*) was weakly up-regulated in hippocampal neurons (S1 Table) but was above the probability threshold for prediction as a CaMKIIα substrate in the KinSwing analysis. Of the presynaptic proteins in Fig 2C, RIM1, piccolo, and known substrate synapsin 1 (S5A, S5D and S5E Fig) were supported by probability as CaMKIIα substrates (S7B Fig). Bassoon had many long-lasting up-regulated phosphorylation sites, which can be considered as putative CaMKIIα substrates (Fig 2B, synaptosome data), but only S2845 and S1126 were supported by probability as CaMKIIα substrates (*P* < 0.05). Likely, many more substrates exist in nonlinear motifs, defying prediction. In these cases, the pattern of regulation may be useful for predicting kinase substrate relationships. Not all putative CaMKIIα substrates were up-regulated or regulated in the same direction on the same protein, indicating incorrect prediction or site-specific effects of CaMKIIα or opposing protein phosphatase activity that depended on localization or other biological factors. Nevertheless, correlation of protein kinase activity patterns, via KinSwing and regulatory sites, with substrate phosphorylation patterns allowed for the identification of approximately 20 putative CaMKIIα substrates.

Clusters vii, viii, and ix were exclusively made up of CaMK and protein kinase A, G, and C (AGC) family members and were generally up-regulated at 10 s and 300 s after 20 mM KCl (Fig 6B and 6C). The PKAα subunit (*PRKACA*) was the most up-regulated at 300 s after 20 mM KCl, but unlike CaMKIIα, its inferred activity was much reduced with increased stimulation strength (76 mM KCl). PKCα (*PRKCA*) in cluster ix underwent the most rapid reversal from positive to negative KS score from 10 s to 90 s after 20 mM KCl. Another pattern was that the PKA, CaMK-like (CaMKL), sterile 20 (STE20), and ribosomal S6 kinase 1 (RSK) classes of protein kinases had positive inferred activity after 20 mM KCl stimulation that was diminished or ablated after 76 mM KCl stimulation (S6B Fig). The PKC class separated into its own cluster (S6B Fig) and was not as affected by the stimulation level as PKCα individually (Fig 6B). PKCζ (*PRKCZ*) had a stimulation level–independent negative KS score for all poststimulus time points (Fig 6B and 6C cluster v), which most resembled the down-regulated phosphopeptide clusters 3 and 5 in Fig 4A. Thus, the inferred activity of some subclasses of the CaMK and AGC families were up-regulated at particular times and were dependent on the strength of the stimulus.

The CDK, glycogen synthase kinase 3 (GSK3), MAPK, and CLK (CGMC) family members CDK1/2/5, GSK3α/β, and MAPK1/3/8/14 in clusters i and ii (Fig 6B and 6C) had the most negative KS scores at 10 s, implying rapid deactivation of CMGC kinases and/or dephosphorylation of the putative CGMC substrates. GSK3β S9 up-regulation was detected at 10 s in hippocampal neurons, which inhibits GSK3β [44]. Protein kinase Bα (AKT1), which targets GSK3β S9, also had corresponding up-regulated phosphorylation at S129, which induces activity (S1 Table) in agreement with a positive AKT1 KS score at 10 s (Fig 6B). However, GSK3β KS scores were negative at all times after 76 mM KCl, and this did not correlate with increased AKT1 KS scores. Higher protein phosphatase activation may have had a greater effect on GSK3β substrates than AKT1-mediated GSK3β inactivation after 76 mM KCl. At 300 s after 20 mM KCl, MAPK1 had the highest inferred activity of cluster ii (Fig 6B). In contrast, at 300 s after 76 mM KCl stimulation, the positive KS score was ablated, indicating dominance of protein phosphatases in both stimulus and poststimulus periods following 76 mM KCl stimulation. KS profiles implicate cluster ii protein kinases, particularly MAPK1 in the poststimulus up-regulated cluster 4 pattern in Fig 4A.

In summary, several patterns of inferred protein kinase activity emerged. CaMKIIα was active under all conditions, aligning with the high-magnitude up-regulated cluster 1 in Fig 4A. A subset of CMGC protein kinases, including MAPKs, CDKs, and GSK3α/β, had negative KS scores at 10 s (Fig 6B), aligning with the high-magnitude down-regulated pattern of Fig 4A cluster 2. Poststimulus activity of MAPKs, PKA, and other protein kinases could contribute to the recovery of cluster 2 substrates or the poststimulus up-regulated pattern of cluster 4 in Fig 4A. Many protein kinase contributions were ablated after stronger stimulus. Thus, our newly developed KinSwing analysis identified protein kinases potentially contributing to the patterns of regulated phospho-signalling.

Using our activity-dependent phosphoproteome of hippocampal cultured neurons, we independently calculated KS scores for each protein kinase (Fig 6B). The response at 10 s after 76 mM KCl stimulation was highly correlated with that of synaptosomes (Fig 6D, R^2^ = 0.81). Casein kinase 2α (CK2α; *CSNK2A1*) was the only protein kinase with substantial deviation between synaptosomes and hippocampal neurons (Fig 6D). CK2α inferred activity also deviated greatly between 20 mM and 76 mM stimulations, resulting in a unique cluster (cluster iii, Fig 6B and 6C). Interpretation of the inferred CK2α activity requires further experiments. Overall, the high correlation supports the concept that the synaptosomes and hippocampal neurons had a similar signalling network.

### Networks of perturbed phospho-signalling at the presynaptic terminal

**Fig 7:**
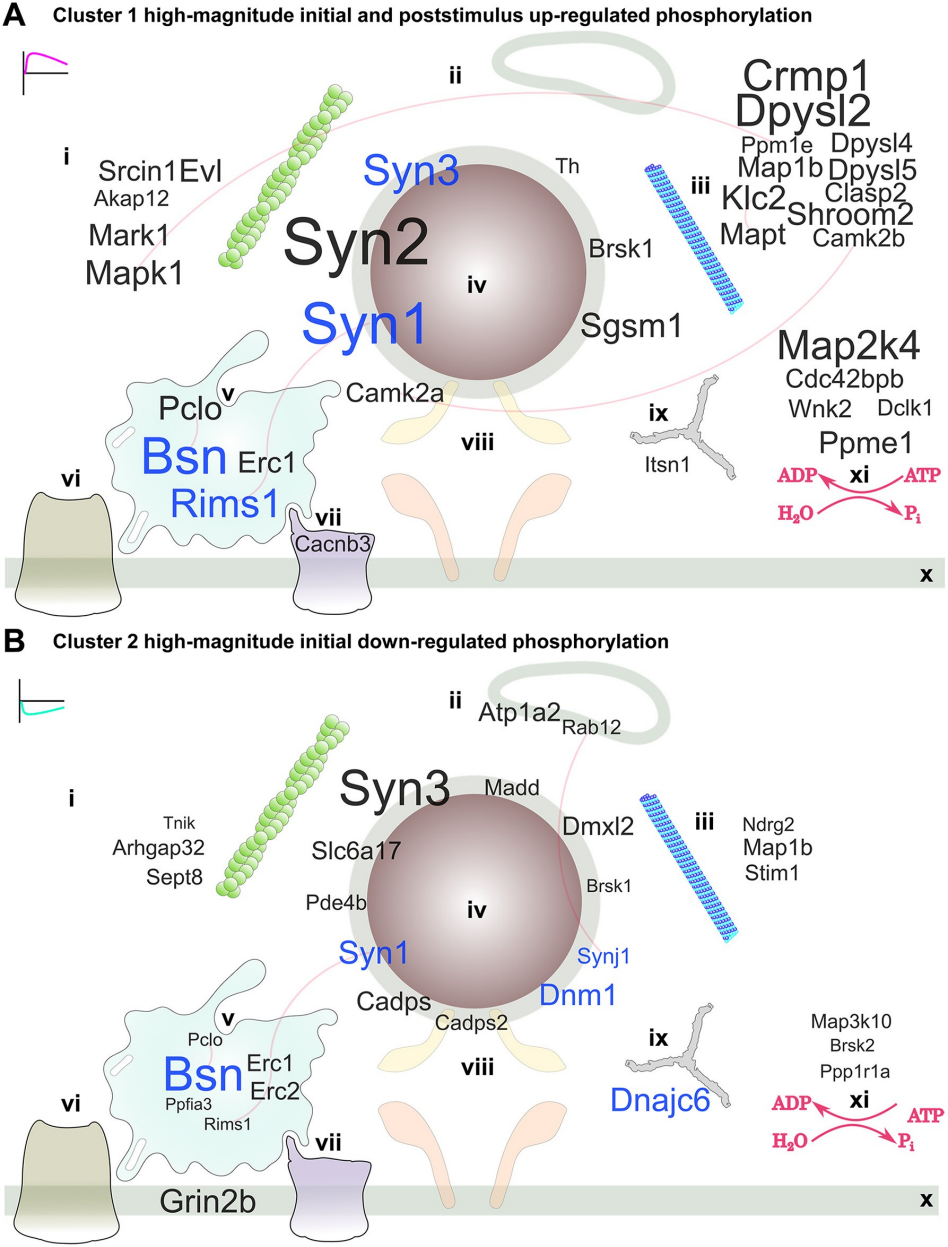
Cluster 1 high-magnitude up-regulated phosphorylation and cluster 2 high-magnitude down-regulated phosphorylation substrates within a network of presynaptic proteins. A word cloud visualization of the synaptosome phosphoproteomics data using gene names anchored to cellular component ontology (subcellular localization). The size of the gene name is scaled to the absolute value of the largest change in the log_2_ intensities at any time point or level of stimulation for (A) cluster 1 and (B) cluster 2 phosphopeptides. Localizations: (i) cytoskeleton and actin cytoskeleton, (ii) endosome, (iii) microtubule and microtubule organizing centre, (iv) synaptic vesicle, (v) active zone scaffold, (vi) voltage-gated potassium channel, (vii) voltage-gated calcium channel, (viii) SNARE complex, (ix) clathrin-coated pit, and (x) presynaptic membrane and membrane raft. (xi) Protein kinases and protein phosphatases, including regulatory subunits, with no specific localization are also shown. Gene names in blue letters have three or more responding phosphorylation sites. Experimentally verified protein interactions (STRING) are shown as red edges, i.e., connecting lines. The data are the result of six independent experiments for each stimulation condition (20 mM and 76 mM KCl). SNARE, soluble N-ethylmaleimide-sensitive factor attachment protein receptor.

To guide our spatial understanding of phospho-signalling at the site of vesicle release and visualize activity-dependent protein targets in the stimulus and poststimulus periods, we generated curated protein interaction networks of the active zone and periphery for each of the clusters determined in Fig 4A. In Fig 7A and 7B, we mapped the protein networks of clusters 1 and 2. Proteins were represented by their gene names, and the letter size was scaled by the highest up- or down-regulated change at any time point to emphasize strongly responding proteins. The blue letters indicate proteins with ≥3 represented phosphorylation sites. Active zone scaffold proteins, synapsins, and cytoskeletal and endocytic proteins were rapidly responding targets of activity-dependent phospho-signalling in clusters 1 and 2 (Fig 7A and 7B), of which a subset were identified previously as activity-dependent [27]. Up-regulated cluster 1 also included strongly responding protein kinases. This included activity-inducing phosphorylation of CaMKIIα (S7A Fig) and MAPK1 (Fig 7A).

To allow a comparison of the proteins affected by each cluster, substrates identified in down-regulated cluster 3 (S7C Fig), low-magnitude down-regulated cluster 5 (S8A Fig), and low-magnitude up-regulated cluster 6 (S8B Fig) were also presented as presynaptic protein interaction networks. Each cluster was also mapped separately onto the same, unfiltered protein network in S1–S6 Files. In each case, the average poststimulus log_2_ intensities were used to scale the gene name letter size. Activity-dependent vesicle and mitochondrion transport proteins syntabulin (*Sybu*) [45] and syntaphilin (*Snph*) [46] featured in the cluster 3 network as possessing multiple down-regulated phosphorylation sites (S7C Fig). Multiple voltage-gated Ca^2+^ channel subunits had down-regulated phosphorylation sites in cluster 5 (S8A Fig), and down-regulated phosphorylation was frequent on many cytoskeletal proteins. Voltage-gated K^+^ channel subunits and synaptic vesicle and exocytosis proteins had up-regulated phosphorylation in cluster 6 (S8B Fig), accounting for the enrichment of related gene ontology terms in Fig 5A–5C.

### A potential role for MAPK in poststimulus up-regulated phosphorylation of a network of presynaptic proteins

**Fig 8:**
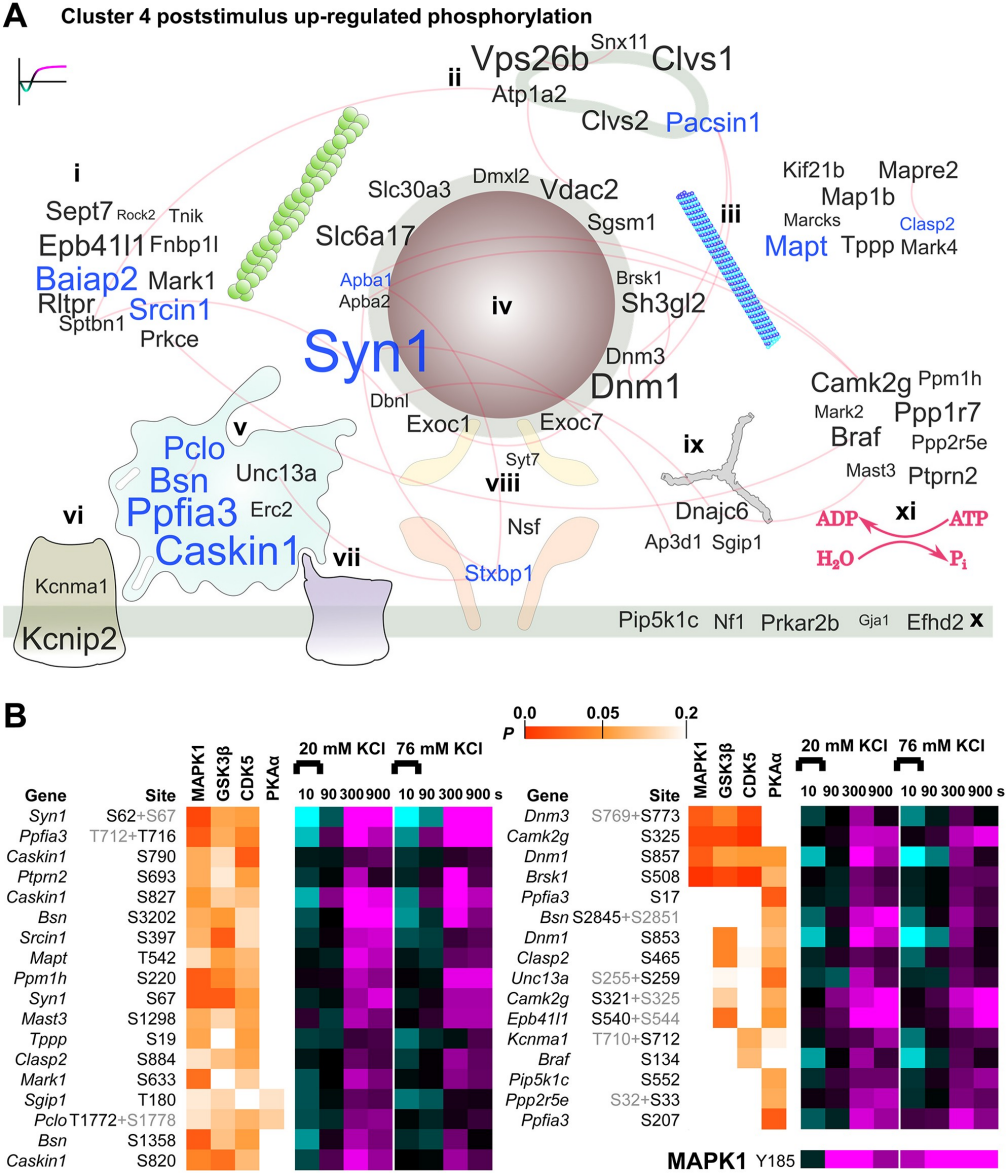
Cluster 4 poststimulus up-regulated phosphorylation substrates within a network of presynaptic proteins and protein kinase substrate probabilities. (A) A word cloud visualization of the synaptosome phosphoproteomics data using gene names anchored to cellular component ontology (subcellular localization). The size of the gene name is scaled to the absolute value of the difference between the log_2_ intensities after 20 mM KCl stimulation for 10 s versus 300 or 900 s (maximum value) for cluster 4 phosphopeptides. Localizations: (i) cytoskeleton and actin cytoskeleton, (ii) endosome, (iii) microtubule and microtubule organizing centre, (iv) synaptic vesicle, (v) active zone scaffold, (vi) voltage-gated potassium channel, (vii) voltage-gated calcium channel, (viii) SNARE complex, (ix) clathrin-coated pit, and (x) presynaptic membrane and membrane raft. (xi) Protein kinases and protein phosphatases, including regulatory subunits, with no specific localization are also shown. Gene names in blue letters have three or more responding phosphorylation sites. Experimentally verified protein interactions (STRING) are shown as red edges, i.e., connecting lines. (B) The phosphorylation sites from (A) with *P* < 0.1 prediction as substrates of MAPK1, GSK3β, CDK5, and PKAα, by KinSwing, shown as a heat map, with the indicated colour scale alongside the heat map of log_2_(stimulated intensity/control intensity) using the same colour scale as in Fig 2B. For data derived from multisite phosphorylated peptides, the nonrelevant phosphorylation site is shown in grey lettering. The up-regulation across time of an activity-inducing MAPK1 phosphorylation site is also shown. The data are the result of six independent experiments for each stimulation condition (20 mM and 76 mM KCl). Underlying data for this figure can be found in S1 Data. CDK5, calcium-dependent kinase 5; GSK3β, glycogen synthase kinase β; MAPK1, mitogen-activated protein kinase 1; PKAα, protein kinase Aα; SNARE, soluble N-ethylmaleimide-sensitive factor attachment protein receptor.

The poststimulus up-regulation observed for cluster 4 is of particular interest because the pattern of regulation implies a late but significant change in phospho-regulated protein function (Fig 5D). Cluster 4 substrates are displayed on the curated protein interaction network in Fig 8A. Letter size was mapped to the largest difference in magnitude from 10 s to 300 or 900 s after 20 mM KCl to identify the largest up-regulated phosphorylation relative to early time points. Motif analysis (S6A Fig) and KinSwing (Fig 6B) predicted that MAPK is likely to have phosphorylated cluster 4 substrates. In addition, a MAPK1 activation site was up-regulated at all poststimulus time points (Fig 8B, bottom). Thus, we compared the probability of a confident match for cluster 4 substrates to the MAPK1 substrate motif, as a heat map in Fig 8B (S1 Data), using the first step of the KinSwing process to generate probabilities (Fig 6B). Alongside, we also compared GSK3β and CDK5, which co-clustered with MAPK1, and PKAα, which was the most up-regulated at 300 s after 20 mM KCl (Figs 6B and 8B). Thus, we present the phospho-signalling network for cluster 4 and identify known and potential substrates of MAPK1 and other protein kinases.

Phosphorylation of (the protein expressed from) *Pip5k1c* at S552 (Fig 8B) promotes its role in the formation of focal adhesions [47]. B-Raf (*Braf*) phosphorylation at S134 is known to be up-regulated in a feedback loop within the MAPK pathway [48] and was up-regulated in cluster 4 (Fig 8B). Phosphorylation of two sites on IRSp53 (*Baiap2*) by a CaMKL family protein kinase Par1b (*Mark2*), itself a cluster 4 member, may negatively regulate cell polarity and spreading [49]. The calcium-activated potassium channel subunit alpha-1 (*Kcnma1*) was up-regulated from 90 s at S712, which is known to cause reduced probability of channel opening and is expected to be a substrate of PKC [50]. These phosphorylation sites with known functions in diverse processes can now be put into the context of neuronal activity through determination of their patterns of phospho-signalling.

In contrast to Fig 7A, in which all synapsins were prominent, only synapsin 1 had cluster 4 phosphorylation sites (Fig 8A). Synapsin 1 domain B sites S62 and S67 (Fig 8B) and S549 in domain D (Fig 3C) are known MAPK substrates [12, 28]. MAPK phosphorylation of synapsin 1 limits neurotransmitter release [18]. Active zone scaffold proteins were major targets within the rapidly responding clusters 1 and 2 (Fig 7A and 7B) and also within the poststimulus up-regulated phosphorylation of cluster 4, having four proteins with at least three represented phosphopeptides (Fig 8A). However, a different set of proteins and phosphorylation sites were prominent in cluster 4: liprin-α3 (*Ppfia3*), Munc13-1 (*Unc13a*), and Ca^2+^- and calmodulin-dependent serine protein kinase-interacting protein 1 (*Caskin1*). This indicates that a poststimulus phospho-signalling pathway targets the active zone scaffold using a distinct subset of protein components and activity-dependent sites. Phosphorylation of S2845 on bassoon promotes 14-3-3 binding and changes bassoon molecular exchange rates with the proposed effect of dissociation from the cytomatrix at the active zone [51]. S2845 is an in vitro RSK family substrate [51], and the RSK family had a pattern of inferred regulation that was consistent with cluster 4 (S6B Fig). Dynamin 1 was poststimulus up-regulated at S857, a phosphorylation site that is de-enriched at presynaptic terminals [38]. The up-regulation of dynamin 1 phosphorylation is inhibitory for synaptic vesicle supply [17], which could promote a depression of vesicle release. The dynamin 1 phospho-dependent binding partner syndapin 1 (*Pacsin1*) [52] was among a group of endosomal proteins that were prominent in cluster 4 (Fig 8A) but not cluster 1 and 2 (Fig 7A and 7B). The phosphorylation of Munc18-1 (*Stxbp1*) on Y473 has been shown to inhibit neurotransmitter release [53] and was one of three Munc18-1 sites with poststimulus up-regulation. Two other cluster 4 proteins were associated with the vesicle fusion machinery (Fig 8A, *Syt7* and *Nsf*). Thus, MAPK1 and other protein kinases are implicated as regulators of cluster 4. MAPK1 may be upstream of protein kinases in cluster 4, but this is currently only supported by motif prediction (Fig 8B). Many substrate sites in Fig 8B have not been functionally investigated; however; the known roles of bassoon, dynamin 1, synapsin 1, and Munc18-1 implicate the poststimulus up-regulation of cluster 4 in influencing the synaptic vesicle cycle.

## Discussion

This first study of the temporal dynamics of the presynaptic phosphoproteome has led to a number of new insights and provides a valuable resource for future analyses. Firstly, it is now apparent that activity-dependent poststimulus phospho-signalling is complex and long-lasting and targets distinct protein domains and networks of phosphoproteins across time. Secondly, initial activity-dependent phospho-signalling in synaptosomes closely correlates with signalling in cultured hippocampal neurons, despite the more numerous compartments harvested with the latter. Thirdly, we have identified specific protein kinases that mediate the initial and poststimulus response, which we have analyzed at unprecedented depth. This achievement was facilitated by our development of a new computational method, KinSwing. These insights greatly advance our knowledge about the stimulus-coupled dynamics of phospho-signalling at the nerve terminal.

A key feature of the KinSwing method was to enable a comparison of the inferred protein kinase activity at each time point and condition. The underlying protein phosphatase activity was also indicated. For example, MAPK1 was highly activated by the stimulus, but its substrates were mainly dephosphorylated prior to 300 s. Although KinSwing weights kinase activity by the number of substrates used to build a kinase model, the kinase substrate matching and the extent of representation of the kinome in the output is limited by the availability of protein kinase substrate data. This can be important when considering substrates that are targeted by closely related protein kinases with limited a priori specificity data [33]. In addition, KinSwing best leverages large-scale experimental data that are rich in repeated patterns of phospho-regulation. However, the value of KinSwing and similar approaches will scale well as the size of public phosphorylation site databases increases. Thus, KinSwing can be applied to any phosphoproteomics study, including any neuronal stimulation paradigm amenable to biochemical analysis, and demonstrates the value of using and developing probabilistic methods for future phosphoproteomics studies.

KinSwing identified CaMKIIα as a protein kinase with a large change in inferred activity across the time course that scaled with stimulation strength and CaMKIIα activity correlated with the profile of high-magnitude up-regulated phosphorylation (Fig 4A, cluster 1). CaMKIIα activation is known to enhance neurotransmitter release [54], but very few presynaptic substrates and mechanisms are known. The putative substrates identified here in the active zone scaffold, including bassoon, piccolo, liprin-α3, and RIM1, might participate in phospho-regulated mechanisms that promote vesicle release and presynaptic plasticity.

Calcineurin is the primary candidate for the observed high-magnitude dephosphorylation (Fig 4A, cluster 2), since known substrates associated with synaptic vesicles [12, 13] were enriched in this phospho-signalling pattern (Fig 5A). Protein phosphatase 2A (PP2A) is also a candidate, since it can undergo Ca^2+^-dependent activation [55]. The high-magnitude up-regulation (cluster 1) lasted longer than the high-magnitude dephosphorylation (cluster 2, Fig 4A–4C). This observation fits with the knowledge that calcineurin or PP2A are dependent on elevated calcium, but CaMKIIα becomes autoactivated [56]. Stronger stimulation increased the magnitude of dephosphorylation for the initially down-regulated patterns (Fig 4A–4C, clusters 2 and 4). This resulted in a delayed return to prestimulus levels and a dampened poststimulus up-regulation for the bidirectional pattern (Fig 4A–4C, cluster 4). Mechanistically, the former could be achieved by sustained inactivation of protein kinases (cluster 2), such as the inhibition of GSK3β [44]. For the latter, initial stronger down-regulation could have dampened up-regulation (cluster 4); however, an alternative mechanism could be the increased activation of protein phosphatases. Phosphatases could have feasibly kept up-regulation low at 900 s after 76 mM KCl while MAPK1 activation was high (Fig 8B). Protein phosphatase 1 (PP1) acts downstream of calcineurin [57, 58]. PP1 and PP2A account for the vast majority of basal phosphatase activity in presynaptic terminals [57] and are implicated in the regulation of neurotransmitter release [59–61].

Poststimulus up-regulated phosphorylation (cluster 4, Fig 8A) was associated with the increased activity of MAPK1 (Fig 6B and S6A Fig). KinSwing predicted that PKAα, CaMKL, STE20, and RSK classes of protein kinases also contributed to poststimulus up-regulation. These predictions were supported by evidence from the literature [49–51]. Substrates of poststimulus up-regulation included synapsin 1, Munc18-1, and distinct components of active zone scaffold proteins. Endocytosis and endosomal proteins were also implicated (Fig 8A). MAPK1/3 activation and targeting of synapsin 1 was found by others to have a negative effect on posttetanic enhancement [18]. In this context, the poststimulus up-regulation may be a homeostatic plasticity mechanism that dampens the effects of strong stimuli. The up-regulated phosphorylation we identified involves a possible MAPK-signalling pathway that has many more components beyond synapsin 1 (Fig 8B).

Activity-dependent phosphorylation in synaptosomes and hippocampal neurons was highly correlated for presynaptic proteins at the common 10-s time point (Fig 2A). Active zone scaffold proteins and synapsins were more highly targeted than postsynaptic scaffold proteins and neurotransmitter receptors (Fig 2C). This indicates that the active zone scaffold proteins may be more finely tuned to respond to Ca^2+^ influx, despite vastly greater postsynaptic abundance of Ca^2+^-sensitive proteins such as CaMKIIα. We have identified bassoon as the major presynaptic signalling hub and the most targeted protein for activity-dependent phospho-signalling within hippocampal neurons. Piccolo, RIM1, microtubule-regulating MAP1B, and tau, as well as SNAP25-interacting protein, were also identified as candidate signal integrators. Thus, phospho-signalling in synaptosomes and intact neurons was similar, and several presynaptic proteins were identified as potential signal integrators, of which bassoon was most prominent.

Our 10-s stimulation with KCl would have caused a large calcium influx, which likely exceeds that produced by electrophysiological stimulation. Thus, our results likely include some nonphysiological phospho-signalling, necessitating validation of our work with electrophysiological stimulation paradigms. However, the strength and duration of stimulation may be more important from a physiology prospective than the method of depolarization. A 10-s high-frequency (≥40 Hz) electrical stimulation, combined with phospho-specific antibody detection, produced virtually identical phospho-signalling responses for the proteins dynamin 1, Akt/PKB, and GSK3β [62]. High-frequency firing rates within the brain are not typical but occur naturally in specific neurons during tasks such as spatial navigation [63]. Obtaining meaningful biochemical measurements at low frequencies will be challenging. In this work, we could have missed regulated phosphorylation sites because of undersampling but also because the change in phosphorylation was too low to measure (despite functional significance). In general, our lower potassium concentration produced robust phospho-signalling, particularly during the poststimulus (S2B and S2C Fig), indicating that high potassium concentrations can potentially be avoided. Nevertheless, elevated potassium stimulation is highly physiologically relevant to understanding the molecular consequences of traumatic brain injury [64] and in the understanding of homeostatic plasticity mechanisms, since pretreatment with elevated potassium was shown to be neuroprotective in a model of excitotoxicity [65]. Our 10-s stimulation was relatively acute, compared to the previous sustained depolarization [27], and allowed for the first examination of the poststimulus response. Stimulating synaptosomes at a high and low level increased our ability to discern Ca^2+^ concentration–dependent patterns of temporal regulation. Future work will require a combination of synaptic vesicle turnover and electrophysiological measurements in neurons to obtain a clearer view of the functional consequences of our stimulations and phosphoproteome dynamics.

The value of information on activity-dependent phosphorylation is in knowing which protein domains and functions are mechanistically linked to neuronal activity, while ruling out phosphorylation that is independent of activity. Our studies revealed that the vast majority of phospho-regulated functions remain unidentified. The lack of association with activity indicates that neuroscience-relevant signalling mechanisms have not yet been a significant focus for research, which might be explained by known biases in research effort [66]. Here, we have highlighted the activity-dependent phosphorylation site data relevant to presynaptic mechanisms and neurotransmitter release. The sensitivity of presynaptic phospho-signalling, if translated into major functional changes, could have implications for the pharmacological targeting of protein kinase/phosphatase activity within neurons. Other important targets of phospho-signalling that regulate diverse cellular functions are contained within this resource for neuroscientists. Therefore, this resource, and our highly developed analysis approach, will allow others to further decipher the functional significance of synaptosomal and neuronal activity-dependent phosphorylation.

## Materials and methods

### Ethics statement

All experiments involving rats were conducted according to authorized procedures and with ethics approval from the Children’s Medical Research Institute/Children’s Hospital at Westmead Animal Ethics Committee (projects C116 and C353).

### Isolation of presynaptic terminals (synaptosomes)

Eight- to 20-wk-old Sprague-Dawley male rats were humanely killed by decapitation. The whole brain was extracted. P2 fraction synaptosomes were isolated and prepared as described previously [67], with minor modifications. Briefly, the S1 fraction was centrifuged at 948*g* for 10 min. All centrifugation steps were performed at 4 °C. The supernatant was discarded, and the P2 fraction was resuspended in a Krebs-like buffer solution (118 mM NaCl, 4.7 mM KCl, 20 mM 4-[2-hydroxyethyl]-1-piperazineethanesulfonic acid [HEPES]/trisaminomethane [Tris] [pH 7.4], 25 mM NaHCO_3_, 1.18 mM MgSO_4_, 1.2 mM CaCl_2_, 0.1 mM Na_2_HPO_4_, 1.85 g/L glucose), which had previously been bubbled with carbogen (95% O_2_, 5% CO_2_) for 45 min to produce a final pH of 7.3–7.5. The solution was centrifuged at 13,800*g* for 10 min, and the resulting pellet was resuspended in 10 mL Krebs-like solution and centrifuged at 948*g* for 10 min to obtain a P2 synaptosome pellet. The pellet was resuspended in 1.75 mL of Krebs-like solution, rested at 37 °C for 45 min, and then placed on ice until stimulated. The number of biological replicates is reported for each experiment. For the mass spectrometry and the western blotting, each biological replicate was produced using the brain tissue of an individual rat.

### Stimulation of presynaptic terminals for phosphorylation analysis

Prior to stimulation, the synaptosomes were warmed to 37 °C for 5 min. To depolarize the synaptosomes, aliquots of 300 μL of synaptosomes were mixed with an equal volume of solution such that the final KCl concentration was 20 or 76 mM, and the sodium concentration was lowered by a similar amount such that the monovalent salt and osmotic concentration was constant. The depolarization continued for 10 s only before centrifugation at 13,500 rpm in a CM-50 MP (ELMI, Riga, Latvia) benchtop centrifuge. To repolarize the synaptosomes, they were resuspended in Krebs solution and incubated for up to 15 min. Samples were collected after 10 s of mock treatment or depolarization and at 90 s, 300 s, and 900 s in the repolarization solution. Prior to lysis, the synaptosomes were pelleted by centrifugation and the supernatant removed. The synaptosomes were lysed in a 300 μL solution and briefly agitated with a benchtop vortex. The lysis solution consisted of 2% SDS, 25 mM HEPES/Tris (pH 7.4), 1 mM ethylenediaminetetraacetic acid (EDTA), 1 mM ethylene glycol-bis(β-aminoethyl ether)-N,N,N′,N′-tetraacetic acid (EGTA), 1x Roche Complete protease inhibitor cocktail, and 1x Calbiochem Phosphatase inhibitor cocktail II. The samples were incubated at 85 °C for 10 min to ensure inactivation of proteases and phosphatases.

### Comparison of the effect of elevated KCl concentration on suspended versus pelleted synaptosomes

Synaptosomes were prepared as above. Synaptosomes were stimulated with 76 mM KCl or mock stimulated with 4.7 mM KCl for 10 s, centrifuged to remove the liquid, and then lysed, as above. In a third condition, synaptosomes were centrifuged, and then KCl was added to achieve an isotonic solution of 76 mM KCl, as in the stimulated condition, but without disturbing the pellet. After 45 s, the supernatant was removed, and the sample was lysed. Equal amounts of each sample were applied to SDS-PAGE and examined by western blot with anti-synapsin 1 pS603 (see “SDS-PAGE and western blotting” section below).

### Preparation of synaptosome samples for large-scale phosphoproteomics using dimethylation

Samples were subjected to reduction with 10 mM dithiothreitol (DTT) for 45 min at 56 °C followed by alkylation with 20 mM iodoacetamide for 30 min in the dark at 23 °C. Another 10 mM DTT was added to quench excess iodoacetamide. The protein content of each sample was then precipitated using methanol/chloroform precipitation [68]. The dry protein pellets were redissolved in 90 μL 8 M urea and 10 μL 1 M triethylammonium bicarbonate (TEAB) and digested for 2 h at 23 °C using 0.15 U endoproteinase Lys-C per sample. The samples were then diluted to 1 M urea with 50 mM TEAB followed by digestion with 20 μg TrypZean trypsin (Sigma-Aldrich, St. Louis, MI, United States) per sample. After 4 h, another 20 μg was added and the samples digested for another 4 h (both steps performed at 23 °C). Digested samples were then subjected to in-solution reductive dimethylation [69] essentially according to Boersema and colleagues [70]. Labelling was performed according to the below scheme (note: control is mock stimulated for 10 s):

1. Replicates 1 and 2:
  - 20 mM KCl set 1: Control (light), 10 s (medium), 90 s (heavy)
  - 20 mM KCl set 2: Control (light), 300 s (medium), 900 s (heavy)
  - 76 mM KCl set 1: Control (light), 10 s (medium), 90 s (heavy)
  - 76 mM KCl set 2: Control (light), 300 s (medium), 900 s (heavy)
2. Replicates 3 and 4:
  - 20 mM KCl set 1: Control (medium), 10 s (heavy), 90 s (light)
  - 20 mM KCl set 2: Control (medium), 300 s (heavy), 900 s (light)
  - 76 mM KCl set 1: Control (medium), 10 s (heavy), 90 s (light)
  - 76 mM KCl set 2: Control (medium), 300 s (heavy), 900 s (light)
3. Replicates 5 and 6:
  - 20 mM KCl set 1: Control (heavy), 10 s (light), 90 s (medium)
  - 20 mM KCl set 2: Control (heavy), 300 s (light), 900 s (medium)
  - 76 mM KCl set 1: Control (heavy), 10 s (light), 90 s (medium)
  - 76 mM KCl set 2: Control (heavy), 300 s (light), 900 s (medium)

After dimethyl labelling, each set of light, medium, and heavy samples were mixed and acidified to 0.2% trifluoroacetic acid (TFA). Insoluble material was removed by centrifugation.

### Phosphopeptide enrichment and peptide fractionation

Samples were then subjected to phosphopeptide enrichment and fractionation by TiSH (TiO_2_, sequential elution from immobilized metal affinity chromatography [SIMAC] and hydrophilic interaction liquid chromatography [HILIC]) [30]. While multiphosphorylated peptides from the SIMAC procedure [71] were analyzed directly by LC-MS/MS, monophosphorylated peptide fractions were fractionated using HILIC. Samples were dissolved in 2 μL dimethyl sulfoxide, followed by 18 μL H_2_O, 180 μL acetonitrile (AcN), and 2 μL 10% TFA. Samples were then fractionated by HILIC using a 1 × 250-mm high-performance liquid chromatography (HPLC) column packed with 5-μm TSKGel Amide 80 resin (Tosoh, Tokyo, Japan) using a Thermo Scientific Ultimate 3000 HPLC system (Thermo Scientific, Bremen, Germany) operated at 50 μL/min. One-minute fractions were collected throughout the increasing aqueous solvent gradient going from 100% to 60% B for 35 min (B solvent: 90% AcN, 0.1% TFA, A solvent: 0.1% TFA). These fractions were pooled based on the UV absorption chromatogram recorded by an Ultimate 3000 variable wavelength detector at 210 nm to produce 10 fractions containing similar amounts of peptide, which were dried by vacuum centrifugation.

### Mass spectrometry of dimethylated peptides originating from synaptosomes

Each fraction was resuspended in 0.15 μL 100% formic acid (FA) followed by 2.4 μL 0.1% TFA and loaded in A solvent 0.1% FA directly onto a homemade 400 × 0.075-mm reversed phase capillary column packed with ReproSil Pur C18 AQ 1.9-μm resin (Dr Maisch, Ammerbuch-Entringen, Germany) using an Ultimate 3000 RSLC nano system (Thermo Scientific, Bremen, Germany). Peptides were analyzed over a 125-min (HILIC fractions) or 180-min (multiphosphorylated fraction) run with a flow rate of 250 nL min^− 1^, being eluted off the column using an increasing gradient from buffer A (0.1% FA) to B (90% AcN, 0.1% FA). Eluted peptides were sprayed into an LTQ-Orbitrap Elite hybrid high-resolution mass spectrometer (Thermo Scientific, Bremen, Germany) via a Proxeon nano-electrospray source (Thermo Scientific, Bremen, Germany) operating at 2.3 kV. A full mass spectrometry scan of the m/z 300–1,800 range was acquired in the orbitrap at a resolution of 60,000 full width at half maximum (FWHM) and a target value of 1 × 10^6^ ions. For each full scan, the 10 or seven (first or second injection replicate, respectively) most intense ions (>+1 charge state) were selected for higher-energy collision dissociation (HCD) and detected at a resolution of 15,000 FWHM. Settings for the HCD event were as follows: threshold for ion selection was 10,000, target value of ions for HCD was 1 × 10^5^, maximum injection time was 500 ms at the mass spectrometry level and 300 or 500 ms (first or second injection replicate, respectively) at the MS/MS spectrometry level, activation time was 0.1 ms, isolation window was 1.8 Da, first fixed mass was 120, and normalized collision energy was 35 or 30 (first or second injection replicate, respectively).

The mass spectrometry proteomics data have been deposited to the ProteomeXchange Consortium [72] via the PRIDE [73] partner repository with the dataset identifier PXD010007.

### MaxQuant processing of mass spectrometry data of dimethylated peptides originating from synaptosomes

Raw data files from the LTQ-Orbitrap Elite instrument were then processed and peak lists generated using MaxQuant version 1.5.2.8 [74]. Database searching of the resulting peak lists was performed using the Andromeda search engine [75] built into MaxQuant against the rat UniProt Reference Proteome database (containing all UniProtKB/Swiss-Prot+TrEMBL rat proteins including isoforms) (downloaded 11 March 2015, containing 29,390 entries). The database search was performed with trypsin/P digestion, allowing a maximum of two missed cleavages. Carbamidomethylation (C) was set as a fixed modification, whereas oxidation (M), acetylation (protein N-terminal), pyro-glutamate (N-terminal E and Q), deamidation (N and Q), and phosphorylation (S, T, and Y) were variable modifications. A maximum of five modifications per peptide was allowed, and the maximum peptide mass was 6,000 Da. The minimum peptide length was seven amino acids, and the default protein contaminants were included in the search. The first search mass spectrometry tolerance was 20 ppm, the main search mass spectrometry tolerance was 4.5 ppm, and the MS/MS tolerance was 20 ppm. Peptide-spectrum match (PSM), protein, and the modification site false-discovery rate were set to 1%. The minimum score for modified peptides was 40. Re-quantify and “match between runs” options were enabled (matching time window 0.7 min, alignment time window 20 min), and second peptide and dependent peptide searching was enabled and disabled, respectively.

### Preparation of samples, mass spectrometry, and data processing for determination of the P2 synaptosomes proteome used in the enrichment analysis

Synaptosomes lysate was generated from mock and 76 mM KCl stimulated synaptosomes at the time points described above. Samples were subjected to reduction with 10 mM tris(2-carboxyethyl)phosphine (TCEP) for 5 min at 85 °C followed by alkylation with 30 mM iodoacetamide for 40 min in the dark at 23 °C. Another 10 mM TCEP was added to quench excess iodoacetamide. Protein content of each sample was precipitated using methanol/chloroform [68]. Dry protein pellets were redissolved in 10 μL 8 M urea and digested for 3 h at 25 °C by adding 5 μg endoproteinase Lys-C (1 μg/μL in 2.5 mM HEPES/NaOH [pH 8.0]) per sample. Promega Sequencing Grade Trypsin (8 μg) in 80 μL 50 mM HEPES/NaOH (pH 8.0) was subsequently added and samples incubated for 16 h at 21 °C. Finally, 15 μg TrypZean trypsin was added to each sample, followed by a 6 h incubation.

The peptides were subjected to labelling with TMT10plex according to the vendor’s instructions. Note that although TMT10plex was used, all quantitative information was discarded, since only a gene list was extracted from this data. The sample was subjected to phosphopeptide enrichment. The TiO_2_ flow-through was desalted using a Waters Sep-Pak Plus C18 cartridge according to the manufacturer’s instructions and retained for LC-MS/MS. Note that no phosphopeptide enrichment analysis of these samples was done. This particular TMT10plex labelled sample was used as an experimental by-product to determine the protein content of synaptosomes.

An aliquot of the TiO_2_ flow-through, containing approximately 2 μg of peptide, was dried completely. The peptide was resuspended in 0.3 μL 100% FA followed by 2.8 μL 0.1% TFA and loaded in A solvent 0.1% FA directly onto a homemade 350 × 0.075-mm reversed phase capillary column with integrated emitter (prepared in-house using a P-2000 laser-based micropipette puller [Sutter Instrument, Novato, CA, US] packed with ReproSil Pur C18 AQ 1.9-μm resin [Dr Maisch, Ammerbuch-Entringen, Germany] using an Ultimate 3000 RSLC nano system [Thermo Scientific, Bremen, Germany]). The peptides were eluted off the column and introduced into a Q Exactive Plus mass spectrometer (Thermo Scientific, Bremen, Germany) via Nanospray Flex nano-electrospray source (Thermo Scientific, Bremen, Germany) operating at 2.3 kV.

Peptides were analyzed over a 240-min run selecting the 12 most intense ions for HCD MS/MS. The settings for the HCD MS/MS event were as follows: minimum automatic gain control target was 5,500, intensity threshold was 50,000, the maximum injection time was 110 ms, isolation window was 1.4 Da, and normalized collision energy was 34. Peptide match was preferred, and dynamic exclusion was 20 s.

Raw LC-MS/MS data files were processed and peak lists generated using MaxQuant version 1.5.8.3 [74]. Database searching of the resulting peak lists was performed using the Andromeda search engine [75] built into MaxQuant against the rat UniProt Reference Proteome database (containing all UniProtKB/Swiss-Prot+TrEMBL rat proteins including isoforms) (downloaded 11 March 2015, containing 29,390 entries). The database search was performed with trypsin/P digestion, allowing a maximum of two missed cleavages. Carbamidomethylation (C) was set as fixed modification, whereas oxidation (M), acetylation (protein N-terminal), pyro-glutamate (N-terminal Q), and deamidation (N and Q) were variable modifications. A maximum of five modifications per peptide were allowed, and the maximum peptide mass allowed was 6,000 Da. The minimum peptide length was seven amino acids, and the default protein contaminants were included in the search. The first search mass spectrometry tolerance was 20 ppm, the main search mass spectrometry tolerance was 4.5 ppm, and the MS/MS tolerance was 20 ppm. PSM and protein, as well as the site false-discovery rate, was set to 1%, and the minimum score for modified peptides was 40. The re-quantify, match between runs, second peptides, and dependent peptide searching options were disabled.

The list of identified proteins from the MaxQuant “proteinGroups.txt” output file was used as the background protein list for the “Enrichment analysis” section below.

### Processing of MaxQuant output

To map and quantify individual phosphosites, we utilized the MaxQuant “evidence.txt” (ProteomeXchange dataset identifier PXD010007) output file containing all identified PSMs for all further analyses. To remove redundancy due to sequences being present as both Swiss-Prot and TrEMBL entries, instances of PSMs to duplicated protein sequences in UniProtKB were removed using a custom Perl script. Only Swiss-Prot entries were kept where exact sequence duplication was observed (two instances: P03926;Q7HKW2 and P05508;Q7HKW3). Then, to determine the amino acid position of phosphosites relative to the full-length canonical protein sequence, a “mapping file” was created (S2 Table), using a custom Perl script. A total of 22,643 unique phosphopeptides were present in the “evidence.txt” file. A total of 11,055 phosphopeptides mapped to a single unique protein accession. Of those phosphopeptides, 10,530 contained more than one protein accession, and 478 mapped to more than one unique gene identifier. Using a custom Perl script, 1,058 were excluded from downstream analyses because they did not contain a localized phosphosite or were contaminant peptides (“CON__”) or those identified in the reverse database by MaxQuant processing for a false-discovery rate of identification (“REV_”). To map the phosphosite positions, we developed a “best” evidence algorithm according to the following rules:

1. For phosphopeptides that mapped to multiple UniprotKB protein accessions, the accession with the highest “protein existence (PE)” value was kept as the best evidence (http://www.uniprot.org/help/protein_existence). Where the protein accession was an isoform (therefore lacking PE information), the PE value was taken from the parent protein.
2. When the PE value was equal, a Swiss-Prot (sp) entry was taken over a TrEMBL (tr) entry.
3. If both entries were Swiss-Prot, the nonisoform was selected.
4. If both entries were isoforms, the lowest-numbered isoform was selected.
5. If the phosphopeptide mapped to multiple unique genes, we determined the “best” annotation for each unique gene (steps 1–5) and recorded this as a multigene-mapped phosphopeptide.

For each of the annotations and phosphosites, the centred sequence of amino acids mapping to each phosphosite was retrieved from the UniProtKB fasta file (i.e., phosphopeptides containing more than one phosphosite would also have more than one centred sequence). Each phosphopeptide identified was then given an identifier, which was composed of the “BEST_PROTEIN” and “BEST_PHOSPHO_POSITION” (e.g., D4A3S7:15, see S2 Table) and was the basis for downstream statistical analysis of phosphorylation.

### Statistical analysis of phosphoproteomics data

To determine the significance of phosphorylation changes over time, the mapping file (S2 Table) and “evidence.txt” (ProteomeXchange dataset identifier PXD010007) were imported into R version 3.4.0 (R Foundation for Statistical Computing, Vienna, Austria) for further analysis. Each entry (phosphopeptide) in the “evidence.txt” file was merged to its corresponding identifier from the mapping file. The MaxQuant normalized intensity for each unique identifier for the 20 and 76 mM KCl 10-s, 90-s, 300-s, and 900-s time point samples was divided by the intensity of the mock-stimulated (4.7 mM KCl 10 s) sample to provide a ratio, which was log_2_ transformed. Where merged phosphopeptides did not contain a minimum phosphosite localization probability score of 0.75 in any of the experiments conducted or were not detected in at least three of the six replicates in either the 20 mM or 76 mM KCl conditions, these phosphopeptides were excluded from further analysis. These criteria retained 6,489 phosphopeptides for the 20 mM KCl treatment and 6,215 unique phosphopeptides for the 76 mM KCl treatment. For the combined 20 mM and 76 mM KCl analysis, this was the intersect of phosphopeptides passing these criteria in both 20 mM and 76 mM KCl treatments, a total of 5,715.

For 20 mM and 76 mM KCl data, these data were normalized independently for separate analyses as well as together (the intersection) for comparative analyses. Initial inspection indicated requirement for normalization and correction of nonbiological sources of variation. For downstream normalization and analysis, we followed the statistical preprocessing method we previously implemented for analysis of phosphoproteomics data [31], and more detail is provided in Waardenberg, 2017 [32]. Data were first rescaled using quantile normalization, assuming that data were missing at random [76], followed by missing value imputation using the k-nearest neighbour approach (k = 10) [77], and data were iteratively reweighted using the surrogate variable analysis [78]. Consistent with previous findings [31], this improved separation of biological samples, where the biological variable of interest (time) was clearly clustering together and separating across the first principle components (Fig 1B).

For statistical comparison of 76 mM or 20 mM KCl data, we fit a linear model with Bayes variance shrinkage using limma [79] and tested for differential phosphorylation at each time point by computing moderated t-statistics (10 s versus control; 90 s versus control; 300 s versus control; 900 s versus control) and the F-statistic for change at any time point and performed pairwise comparisons for each time point. *P* values were corrected for multiple hypothesis testing using the Benjamini and Hochberg method [80]. For 76 mM versus 20 mM KCl, we tested for differential phosphorylation between these concentrations at each time point using the intersected data (as per above). Unless reported otherwise, a corrected *P* value less than or equal to 0.05 was reported as significant.

All data and statistics for each comparative analysis are available in S1 Table.

### Primary hippocampal neuron culture

One-day-old Wistar rat pups were humanely killed by decapitation. The brain was removed and dissected on ice to isolate the hippocampus. The hippocampi from eight pups were incubated in 4 mL dissection buffer (Hank’s Balanced Salt Solution supplemented with penicillin/streptomycin, 1 mM sodium pyruvate, 20 mM HEPES/Tris [pH 7.4]) and 0.45% glucose (Thermo Scientific, North Ryde, NSW, Australia) with 120 μL 43.6 mg/mL (≥104 units) papain solution (Worthington Biochemical Corporation, Lakewood, NJ, US) at 37 °C for 20 min. The buffer was aspirated, and the hippocampi were washed three times in 4 mL Neurobasal medium supplemented with 5% foetal bovine serum, 4 mM GlutaMax, penicillin/streptomycin, and B27 (Thermo Scientific, North Ryde, NSW, Australia) (“NM5 medium”) followed by trituration using glass pipettes with an increasingly smaller tip diameter. The dissociated neurons were suspended in 50 mL NM5 medium and seeded into poly-D-lysine-coated six-well cell culture plates (4-mL suspension per well). The following day, the medium was replaced with Neurobasal medium supplemented with 4 mM GlutaMax, penicillin/streptomycin, and B27 (“NM0 medium”). Until 21 d in vitro (DIV21), half of the medium was replaced with fresh NM0 twice per week.

### Stimulation of primary hippocampal neurons

At DIV21, the neurons were mock stimulated (4.7 mM KCl) or depolarized (76 mM KCl) for 10 s. At the 10-s time point, immediately after aspirating the stimulation buffer, the neurons were lysed by adding 500 μL 20 mM HEPES/NaOH (pH 8.0), 2% SDS, 2 mM EGTA, 2 mM EDTA with 1x Roche Complete protease inhibitor cocktail and 1x Calbiochem Phosphatase inhibitor cocktail II. The lysate was transferred to a 2-mL microcentrifuge tube and frozen on dry ice. At the end of the experiment, the lysates were heated to 90 °C for 5 min to inactivate all proteases and phosphatases.

### Preparation of hippocampal neuron samples for large-scale phosphoproteomics using tandem mass tags (TMTs)

All samples were subjected to reduction with 10 mM TCEP for 5 min at 85 °C followed by alkylation with 30 mM iodoacetamide for 40 min in the dark at 23 °C. The protein content of each sample was precipitated using MeOH/chloroform [68]. Dry protein pellets were redissolved in 20 μL 9 M urea and diluted to 1 M urea with 35 mM HEPES/NaOH (pH 8.0). TrypZean trypsin (8 μg; Sigma-Aldrich, Castle Hill, NSW, Australia) was subsequently added to each sample, followed by incubation for 16 h at 21 °C. The samples were dried by vacuum centrifugation and resuspended in 30 μL 50 mM HEPES/NaOH (pH 8.0).

The peptide samples were subjected to quantitative labelling with TMT10plex according to the vendor’s instructions and the following scheme. Three tags were used for an experiment that was discarded. The discarded experiment was a 20 mM KCl treatment. The experiment was discarded on the basis that well-known activity- and Ca^2+^ influx–dependent protein kinase substrate sites S603 and S566 in synapsin 1 were not up-regulated, and Ca^2+^ influx activity–dependent dynamin 1 phosphatase sites S774 and S778 were not down-regulated (all raw data are available via ProteomeXchange with identifier PXD010007).

1. TMT-126: 4.7 mM KCl, replicate 1
2. TMT-127N: Discarded condition
3. TMT-128C: Not used
4. TMT-128N: 76 mM KCl, replicate 1
5. TMT-128C: 4.7 mM KCl, replicate 2
6. TMT-129N: Discarded condition
7. TMT-129C: 76 mM KCl, replicate 2
8. TMT-130N: 4.7 mM KCl, replicate 3
9. TMT-130C: Discarded condition
10. TMT-131: 76 mM KCl, replicate 3

After 60 min of incubation at 21 °C, 5% hydroxylamine was added to an end concentration of 0.25%, followed by incubation for 20 min at 21 °C. The samples were then mixed and subjected to phosphopeptide enrichment and fractionation using TiO_2_ and HILIC as described for the reductive dimethylation experiment but without performing the SIMAC procedure. Based on the UV absorption chromatogram, the HILIC fractions were pooled into 14 fractions containing similar amounts of peptide, which were dried by vacuum centrifugation prior to LC-MS analysis.

### Mass spectrometry of TMT-derivatized peptides originating from hippocampal neurons

Each fraction was resuspended in 0.3 μL 100% FA followed by 2.8 μL 0.1% TFA and loaded in A solvent 0.1% FA directly onto a homemade 450 × 0.075-mm reversed phase capillary column with integrated emitter (prepared in-house using a P-2000 laser-based micropipette puller [Sutter Instrument, Novato, CA, US]) packed with ReproSil Pur C18 AQ 1.9-μm resin (Dr Maisch, Ammerbuch-Entringen, Germany) using an Ultimate 3000 RSLC nano HPLC system (Thermo Scientific, Bremen, Germany).

Peptides were analyzed using a 180-min LC-MS run with a flow rate of 250 nL min^− 1^ being eluted off the column using an increasing gradient from buffer A (0.1% FA) to B (90% AcN, 0.1% FA). Eluted peptides were introduced into an Q Exactive Plus mass spectrometer (Thermo Scientific, Bremen, Germany) via a Nanospray Flex ion source (Thermo Scientific, Bremen, Germany) operating at 2.3 kV. A full mass spectrometry scan of the m/z 375–1,500 range was acquired at a resolution of 70,000 FWHM, a target value of 3 × 10^6^ ions, and a maximum injection time of 100 ms (mass spectrometry). For each full scan, the 12 most intense ions (charge state +2 to +7) were selected for HCD and detected at a resolution of 35,000 FWHM. The settings for the HCD MS/MS event were as follows: Minimum automatic gain control target was set to 2E5, intensity threshold was 1.0E5, the maximum injection time was 120 ms, isolation window was 1.0 Da, first fixed mass was 120, and normalized collision energy was 34 (stepped collision energy). Peptide match was off, “Exclude isotopes” was on, “dynamic exclusion” was 12 s, and the “If idle” setting was set to “pick others”.

### MaxQuant processing of mass spectrometry data for TMT-derivatized peptides originating from hippocampal neurons

Raw LC-MS/MS data files were processed, and peak lists were generated using MaxQuant version 1.5.2.8 [74]. Database searching of the resulting peak lists was performed using the Andromeda search engine [75] built into MaxQuant against the rat UniProt Reference Proteome database (containing all UniProtKB/Swiss-Prot+TrEMBL rat proteins including isoforms) (downloaded 11 March 2015, containing 29,390 entries). The database search was performed with trypsin digestion, allowing a maximum of two missed cleavages. Carbamidomethylation (C) was set as fixed modification, whereas oxidation (M), acetylation (protein N-terminal), pyro-glutamate (N-terminal Q), deamidation (N and Q), and phosphorylation (S, T, and Y) were variable modifications. A maximum of five modifications per peptide was allowed, and the maximum peptide mass allowed was 6,000 Da. The minimum peptide length was seven amino acids, and the default protein contaminants were included in the search. The first search mass spectrometry tolerance was 20 ppm, the main search mass spectrometry tolerance was 4.5 ppm, and the MS/MS tolerance was 20 ppm. PSM and protein, as well as the site false-discovery rate, was set to 1%, and the minimum score for modified peptides was 40. The “re-quantify” and “match between runs” options were disabled.

### Statistical analysis of MaxQuant output for TMT-derivatized peptides originating from hippocampal neurons

For TMT-derivatized peptides, 47,674 unique phosphopeptides were present in the “evidence.txt” file. Following the same filtering procedure for the dimethylation data, 22,063 unique phosphopeptides remained for statistical analysis. Data were processed essentially as described for the dimethylation dataset. A log_2_ ratio was computed for each of the three paired replicates (76 mM KCl treated intensity/4.7 mM KCl control intensity) using the corrected reporter ion intensities provided in the MaxQuant file. Missing value imputation was not conducted. Thus, phosphopeptides with no, or zero, intensity values were discarded. Log_2_ ratios were then corrected via surrogate variable analysis, which included the discarded conditions (see above) to ensure that all sources of variation were modelled. Limma analysis was used to calculate moderated t-statistics. *P* values were corrected for multiple hypothesis testing using the Benjamini and Hochberg method [80].

All data and statistics for each comparison (dimethylated and TMT) are available in S1 Table.

### Enrichment analysis

The 1,917 unique phosphopeptides from the 20 mM and 76 mM KCl stimulated dimethylation dataset were imported into Perseus 1.5.8.5 for further analysis. Data were k-means clustered by row (each phosphopeptide) with 100 starts and 100 iterations. Six clusters (k) was determined to be optimal for enrichment analysis (S3A and S3B Fig). Thus, very small and very large clusters were avoided for enrichment analysis. Clusters 1 and 2, which consisted of the highest magnitude changes, contained 57 and 44 unique UniProt accessions, respectively. Clusters 3, 4, 5, and 6 contained 130, 129, 451, and 368 unique accessions. The entire set of 1,917 unique phosphopeptides contained 865 unique accessions. Each of these sets were imported into DAVID (database for annotation, visualization, and integrated discovery) [81] for gene ontology enrichment analysis. The protein content of our P2 synaptosomes was determined by LC-MS/MS analysis in a separate experiment (see section “Preparation of samples, mass spectrometry, and data processing for determination of the P2 synaptosomes proteome used in the enrichment analysis” above). The identified proteins contained 3,866 unique accessions. These accessions were combined with the 865 unique proteins accessions from phosphopeptides identified from synaptosomes and 4,947 unique protein accessions from phosphopeptides identified from hippocampal neurons. The intersection of these sets was a list of 7,588 nonredundant protein accessions, which was used as the background. Gene ontology enrichment was queried for “molecular function”, “biological process”, and “cellular component” using “MF_Direct”, “BP_Direct”, and CC_Direct”, since these sets provide direct annotations for each accession [82]. The default threshold *P* > 0.1 was used to extract an initial list of enriched terms, which was also required to have at least four accessions, prior to further filtering. For comparison of clusters, the top five most probable enriched terms/domains from each cluster with *P* < 0.05 were arranged into a heat map of probabilities. Columns (clusters) in the heat map were allowed to exceed the initial limit of five terms if they were highly ranked in another cluster, but each row was required to contain a term with at least *P* < 0.01. The *P* values for each term were hierarchically clustered by row (each term) using Perseus 1.5.8.5.

Phosphorylation site regulation ontology term enrichment was performed as follows. Terms were extracted from the “ON_FUNCTION” and “ON_PROCESS” columns of the “Regulatory_sites.gz” file, downloaded 26 May 2018 from PhosphoSitePlus [33]. The terms are available in S1 Table. Only terms with at least nine matching phosphorylation sites within the set of 1,917 significantly regulated phosphorylation sites from synaptosomes were considered. There were 83, 82, 201, 191, 797, and 563 unique phosphopeptides for clusters 1, 2, 3, 4, 5, and 6, respectively. This included possible redundant multisite phosphorylation assignments, which were kept. The number of occurrences for each term per cluster was determined. Fold enrichment was calculated by considering the ratio between foreground and background terms as a 2 × 2 contingency table of (1) the number of terms matched within a cluster, (2) the size of the cluster, (3) the number of term matches for all 1,917 peptides, and (4) 1,917 as the total background. For each term, significance of the fold enrichment was determined by applying a Fisher’s exact test to the 2 × 2 contingency table (*P* < 0.05). All clusters with no term matches were assumed nonenriched and given a *P* value of 1.

The centred peptide sequences for each of the six clusters were examined for enriched protein kinase substrate motifs using motif-x [40] with the following settings: central character, S; width, 15; occurrences, 20; significance, 0.000001; and background, IPI Rat Proteome.

### KinSwing analysis

Curated substrate sequences for all human protein kinases, which consisted of in vitro and in vivo evidence, were downloaded from PhosphoSitePlus (13 January 2016) [33]. Protein kinase family and class information was downloaded from kinase.com (26 July 2017) [83]. A human substrate dataset was used, since it was much larger than the rat dataset, including increased coverage of the kinome. Tyrosine protein kinases and their substrates were not considered and were removed from the experimental and curated datasets. All analyses were performed in R version 3.4.0 (R Foundation for Statistical Computing, Vienna, Austria).

### Building protein kinase substrate models

For building protein kinase substrate models, we considered the set of *n* centred amino acid sequences (13 amino acids in length, from PhosphoSitePlus), *S*, for each protein kinase, as a set of identity matrices, *K*_*a*,*p*_, for each amino acid, *a*, where *a* ∈ {1,2 … 20}, at each sequence position, *p*, where 1 indicates a match for each amino acid, *a*, at position *p* for the *i-th* amino acid sequence, and 0 indicates no match for amino acid residue, *a*, at position *p* for the *i-th* amino acid sequence:

$$K_{a,p}=\{\begin{array}{ll} 1, & if\;S_{i,p}=a\\ 0, & if\;S_{i,p}\neq a \end{array}$$

and determined the position probability matrix, *M*_*a*,*p*_, for each protein kinase, *K*, as a log likelihood ratio of the average frequency of each amino acid, *a*, at each position, *p*, for the set of sequences, *S*, divided by the background frequencies of amino acids *a*, *B*_*a*_:

$$M_{a,p}=\text{log}\left(\frac{(\frac{1}{n}\sum_{i=1}^{n}K_{i})+C}{B_{a}+C}\right)$$

A pseudocount, *C*, of 0.00001 was added to the position probability matrix to avoid log zero divisions and *B*_*a*_ = 0.05 assuming equal distribution of amino acids.

### Protein kinase substrate scoring and statistical significance

For each input sequence identified in our phosphoproteome (13 amino acids in length, centred on each phosphosite), we then score its match to protein kinase, *K*, as the sum of the corresponding entry, *M*_*ap*_, of matrix, *M*_*a*,*p*_, for each amino acid residue, *a*, at position, *p*, of sequence length *i*:

$$S_{score}=\sum_{i=1}^{n}f(a,p)$$

where

$$f(a,\;p)=M_{ap}\in M_{a,p}$$

We then determined the probability, *P*(*S*_*score*_|*R*, *N*), where the probability of *S*_*score*_ is conditional on a reference distribution of *N* randomly sampled sequences, of 1,000, and *R* sequences determined to have a test statistic less than or equal to *S*_*score*_:

$$P(score)=\frac{R+1}{N+1}$$

where

$$R=\sum_{n=1}^{N}I({(S}_{score})n*\geq {(S}_{score})i)$$

### Protein kinase substrate networks

To build protein kinase substrate networks, we consider the set of protein kinase substrate predictions *P*(*S*_*score*_|*R*, *N*) ≤ 0.05 as edges. *S*_*ipk*_ then represents the product of two logic functions: (1) *S*_*ik*_, directionality, based on the log_2_ fold change, *i*, of each predicted substrate of protein kinase, *k*; and (2) *S*_*pk*_, significance, *p*, of phosphorylation change for each predicted substrate of protein kinase, *k*:

$$S_{ipk}=S_{ik}∙S_{pk},\;S_{ipk}\neq 0$$

where *i* is given and is the log fold change:

$$S_{ik}=f\left(i\right)=\{\begin{array}{ll} -1, & i<0\\ 1, & i\geq 0 \end{array}$$

and *p* is given and is the significance of log fold change, *i*:

$$S_{pk}=f\left(p\right)=\{\begin{array}{ll} 1, & p<0.05\\ 0, & p\geq 0.05 \end{array}$$

*S*_*ipk*_ therefore represents the state of the kinase substrate network, where −1 represents protein kinase substrates significantly differentially phosphorylated and negatively regulated, and +1 represents protein kinase substrates significantly differentially phosphorylated and positively regulated.

### Protein kinase substrate network connectivity

For each protein kinase, *K*, the sum of the absolute values for each protein kinase, *C*_*k*_, therefore represents the overall connectivity of the protein kinase, *K* (i.e., how many substrates were associated with a protein kinase for each condition and time point):

$$C_{k}=\sum_{S_{ipk}\neq 0}\left[S_{ipk}\right]$$

*Pos*_*k*_ represents the positive proportion of edges for protein kinase, *K*,

$${Pos}_{k}=\frac{\sum_{S_{ipk}\geq 0}\left[S_{ipk}\right]}{C_{k}}$$

*Neg*_*k*_ represents the negative proportion of edges for protein kinase, *K*,

$${Neg}_{k}=\frac{\sum_{S_{ipk}<0}\left[S_{ipk}\right]}{C_{k}}$$

*swing*_*k*_ is an overall score of the proportion of positive and negative edges for protein kinase, *K*, weighted for the number of edges, *C*_*k*_, and the number of substrate sequences, *S*_*n*_. A pseudocount, *c*, of 1, is added to *Pos*_*k*_ and *Neg*_*k*_ to avoid log zero divisions, thereby weighting for protein kinases with the greatest swing and greatest evidence of swing based on the number of predicted substrates and the number of substrates used to infer the protein kinase position weight matrix:

$${swing}_{k}={log}_{2}(\frac{{Pos}_{k}+c}{{Neg}_{k}+c})*{log}_{2}(C_{k})*{log}_{2}(S_{n})$$

To permit comparison of kinase activity scores across time, *swing*_*k*_ was finally transformed into a z-score, *Z*(*swing*_*k*_), where *μ* is the mean of all swing scores, and *σ* is the standard deviation of all swing scores:

$$Z({swing}_{k})=\frac{{swing}_{k}-\;\mu }{\sigma }$$

The raw output was filtered such that each reported z-score, *Z*(*swing*_*k*_), which we name the KS score, required (1) at least 19 experimentally validated protein kinase substrate matches and (2) ≥1 time point with a KS score greater than the corresponding standard deviation for that score (such that the KS score deviated from zero). See S1 Table for KS scores and standard deviations for all protein kinases examined across time. KS scores were also calculated for groups of protein kinases that were annotated according to classes, and these results are also in S1 Table. The remaining scores were clustered using the Perseus 1.5.8.5 hierarchical clustering function.

During the course of this work, methods with similarities to KinSwing were published, i.e., KARP (kinase activity ranking using phosphoproteomics data) [84], IKAP (inference of kinase activities from phosphoproteomics) [85], and iKAP (in silico kinome activity profiling) [86]. KARP has a fundamentally different output, resulting in a ranking of protein kinases on a strictly positive scale, not inferring overall directionality of activity. Although iKAP is concerned with quantification of directionality of kinase substrates, they utilize a chi-squared test for testing the proportion of positive and negative phosphosite edges within a sample condition, rather than transforming to a statistic, which is valuable for comparison across multiple conditions and time courses, as we have done. IKAP uses an optimization procedure based on precise kinase substrate database matching followed by correlation, which assumes that kinase substrate networks act in a linear manner. Future studies that determine precision based on experimental prediction would further guide the development of these algorithms.

### Protein interaction network–cellular component mapping

Network edges were determined by submission of a nonredundant list of gene names for the 1,917 significantly regulated phosphopeptides to STRING [87] using *Rattus norvegicus* as the organism. Node and edge data from STRING were subsequently imported into Cytoscape version 3.5.1 [88]. The following *R*. *norvegicus* gene names used by STRING were manually changed to match the gene names used by DAVID and/or UniProt: ENSRNOG00000018712, *Camk2a*; *Dmxl2-ps1*, *Dmxl2*; *LOC687090*, *Tprg1l*; *Sec311*, *Exoc1*. Within Cytoscape, the network was filtered by discarding proteins without specific cellular component annotations. Nodes were required to have the following DAVID “CC_Direct” cellular component annotations, which were grouped into nine categories: (i) cytoskeleton and actin cytoskeleton, (ii) endosome, (iii) microtubule and microtubule organizing centre, (iv) synaptic vesicle, (v) active zone scaffold, (vi) voltage-gated potassium channel, (vii) voltage-gated calcium channel, (viii) soluble N-ethylmaleimide-sensitive factor attachment protein receptor (SNARE) complex, (ix) clathrin-coated pit, and (x) presynaptic membrane and membrane raft. Protein kinases, protein phosphatases, and subunits/regulators of phosphatases were also retained (identified by UniProt gene ontology annotation as of 31 July 2017).

The cellular component data were curated to improve relevance to synaptic biology. The following manual changes were made to cellular component annotation or protein kinase/phosphatase annotation based on UniProt annotations as of 31 July 2017 or references provided: *Syn1* changed from “active zone scaffold” to “synaptic vesicle”, *Syn2* changed from “SNARE complex” to “synaptic vesicle”, *Camk2a* changed from “presynaptic membrane” to “synaptic vesicle”, *Caskin1* annotated with “active zone scaffold” [5], *Stxbp1* annotated with “SNARE complex”, *Nefh* annotated with "cytoskeleton", *Sh3gl2* annotated with "synaptic vesicle", *Syt9* annotated with "synaptic vesicle", *Rab12* annotated with “endosome”, *Rab27a* annotated with "synaptic vesicle", *Rab8b* annotated with "synaptic vesicle", *Sgsm1* annotated with "synaptic vesicle", *Ppfia1* annotated with "active zone", *Exoc1* annotated with "synaptic vesicle", *Exoc7* changed from “microtubule” to “synaptic vesicle”, *Dbnl* annotated with "synaptic vesicle", *Slc30a3* annotated with "synaptic vesicle", *C2cd2l* annotated with "presynaptic membrane", *Dnajc6* annotated with "clathrin-coated pit", *Doc2a* annotated with "synaptic vesicle", *Dnm3* changed from “microtubule” to “synaptic vesicle”, *Cadps* annotated with “synaptic vesicle", *Synj1* changed from “microtubule” to synaptic vesicle, *Ap3b2* and *Ap3d1* annotated with “clathrin-coated pit”, *Dlg4* “synaptic vesicle” annotation was deleted, *Sptbn1* annotated with “actin cytoskeleton”, *Sptbn2* annotated with “actin cytoskeleton”, *Sptbn4* annotated with “actin cytoskeleton”, *Dyrk1b* annotated with “protein kinase”, *Itsn1* changed from cytoskeleton to “clathrin-coated pit”, and *Dpysl3*, *Dpysl4*, and *Dpysl5* annotated with “microtubule”. Each of the 11 groups were separately arranged in Cytoscape 3.5.1 using the “grid layout”. Arbitrary adjustments in the layout of each grid were made to improve clarity; thus, no conclusions should be drawn from the distance between nodes. Membrane proteins were arbitrarily arranged in a line. A subset of inositol kinases and phosphatases were moved to the membrane (*Pip5k1c*, *Inpp4a*, and *Pi4k2a*).

The network was made up of a total of 267 nodes and 907 edges. The edges were coloured red if the “experimentally_determined_interaction” value from STRING was ≥0.3. Proteins were represented by gene names. Proteins with three or more responding phosphorylation sites had gene names presented in blue letters. Letter size for each node/gene name was scaled linearly (from an arbitrary minimum value) by using the quantitative phosphopeptide data. For clusters 1 and 2, the absolute value of the largest log_2_(stimulated intensity/control intensity) after either 20 mM or 76 mM KCl stimulation was used to scale the letters. For cluster 4, the largest up-regulation from 10 s to 300 s or from 10 s to 900 s after 20 mM KCl was used to scale the letter size. For clusters 3, 5, and 6, the average poststimulus log_2_(stimulated intensity/control intensity), using all 20 mM and 76 mM KCl values, was used in scaling letters. Networks for specific clusters were filtered to remove proteins that were not significantly phospho-regulated within the cluster. Unfiltered networks for each cluster can be found in S1–S6 Files.

### SDS-PAGE and western blotting

Samples were separated via SDS-PAGE and transferred to 0.22-μm-pore-size nitrocellulose membrane using the Trans-Blot Turbo Transfer System (both Bio-Rad, Gladesville, NSW, Australia). Blots were blocked with a solution of phosphate-buffered saline (PBS) with 5% (w/v) bovine serum albumin, 0.5% PVP-40, 0.1% (v/v) TWEEN-20 (PBST) for 1–16 h at 4 °C. Blots were incubated with the following primary antibodies and their respective concentrations diluted into PBST:

1. 1:2,000 Anti-synapsin phospho-Ser-603 rabbit polyclonal (612-401-C95, Rockland Immunochemicals, Pottstown, PA, US)
2. 1:2,000 Anti-synapsin phospho-Ser-62+Ser-67 rabbit polyclonal (TA309241, Origene, Rockland, MD, US)
3. 1:1,000 Anti-dynamin phospho-Serine-774 sheep polyclonal [17]
4. 1:2,000 Anti-CaMKII phospho-Thr-286 rabbit monoclonal (D21E4, Cell Signalling Technology, Beverly, MA, US)
5. 1:50,000 Anti-β-actin horseradish peroxidase–conjugated mouse monoclonal (A3854, Sigma-Aldrich Castle Hill, NSW, Australia)

Primary antibody incubation was performed for 1 h at 22 °C, with agitation. Blots were then washed 3 times for 5 min each with PBST and incubated with the following secondary antibodies at their respective concentrations diluted into PBST:

1. 1:20,000 Anti-Rabbit IgG horseradish peroxidase–conjugated pig polyclonal (P0217, Dako, Glostrup, Denmark)
2. 1:20,000 Anti-sheep IgG horseradish peroxidase–conjugated rabbit polyclonal (P0163, Dako, Glostrup, Denmark)

Secondary antibody incubation was performed for 1 h at 22 °C with agitation and was not required for the anti-β-actin antibody. Blots were washed 3 times for 5 min per wash with PBST and then incubated for 5 min with SuperSignal West Pico Chemiluminescent Substrate (Thermo Fisher Scientific, West Ryde, NSW, Australia) solution composed of 1:1 peroxide:luminol enhancer solution. Blots were transferred to a transparent plastic pouch, gently strained of excess substrate, and then imaged using an ImageQuant LAS-4000 Camera (Fujifilm, Brookvale, NSW, Australia). Image files were processed and densitometry was performed using the associated Fujifilm Multi Gauge software.

## Supporting information

S1 Fig Investigation of solution exchange step in synaptosome stimulations. The switch from 20 or 76 mM KCl to 4.7 mM KCl required a centrifugation step and manual exchange of the solution, which was a 35-s (±1 s, standard deviation; *n* = 9) process, using our equipment. If Ca^2+^ could continue to enter the pelleted synaptosomes during the exchange process, then our stimulation would be longer than intended. We investigated the potential for KCl to influence pelleted synaptosomes. We used up-regulation of synapsin 1 S603 as a marker of depolarization. We compared standard stimulation with elevated KCl—i.e., 10-s 76 mM KCl stimulation, centrifugation, and manual solution exchange (35 s) to a 45-s stimulation of the supernatant after centrifugation. This latter condition was used to test if elevated KCl could stimulate/depolarize the pelleted synaptosomes. Volumes and ionic strength were consistent throughout (see Materials and methods). Left: representative western blot of the comparison of 4.7 mM KCl mock treatment and 76 mM KCl depolarizing treatment of suspended synaptosomes alongside a 76 mM KCl treatment of the supernatant of pelleted synaptosomes. Successful depolarization (positive control) was confirmed by western blot with anti-syn1-pS603. Anti-β-actin was used as a loading control. Right: quantification of the western blotting. Bar graph of the average and SEM of three independent experiments (normalized to mock-treated/control sample, one-way analysis of variance and Dunnett’s post hoc test; **P < 0.01). Below: schematic of the experimental procedure for each condition in the bar graph. The failure of high-concentration KCl in the supernatant to up-regulate pS603 allowed us to conclude that KCl present during the solution exchange is unlikely to influence phospho-signalling in pelleted synaptosomes. Thus, our 10-s stimulation paradigm was established as an acute stimulation. Underlying data for this figure can be found in S1 Data.
(PDF)

S2 Fig Comparison of the magnitude and count of the significantly regulated phosphopeptides for 20 mM and 76 mM KCl stimulated synaptosomes at each time point. (A) Plot of 20 mM KCl versus 76 mM KCl log_2_(stimulated intensity/control intensity) phosphorylation level changes at 10, 90, 300, and 900 s. The significant phosphorylation level changes detected in both 20 mM and 76 mM KCl experiments are shown in colour (the number, *n*, of intersecting data points for 20 mM and 76 mM KCl is shown for each time point). Those significant only after 76 mM KCl stimulation are shown in grey. Nonsignificant values are shown in black. The linear fit of significant values for each plot are shown (fitted line was required to pass through zero). Underlying data for this figure can be found in S1 Data. (B) Count of significantly regulated phosphopeptides after 20 mM (grey bars) and 76 mM KCl (black bars) stimulation for each time point. Phosphopeptides were not required to be detected in both stimulation level experiments. Underlying data for this figure can be found in S1 Data. (C) This figure differs from (B) because of the requirement that phosphopeptide signals were detected in both the 20 mM and 76 mM KCl stimulation experiments. Underlying data for this figure can be found in S1 Data. (D) Plot of log_2_(76 mM KCl 10-s stimulated intensity/control intensity) for phosphopeptides from cultured hippocampal neurons versus whole-brain synaptosomes. The plot includes only the 195 phosphopeptides that were significantly regulated in both types of samples. The synaptosome data are the result of six independent experiments for each stimulation condition (20 mM and 76 mM KCl). The cultured hippocampal neuron data are from three independent experiments using 76 mM KCl stimulation. A moderated t-statistic adjusted for multiple hypothesis testing was used to determine the significance of single time points, *P* < 0.05. Underlying data for this figure can be found in S1 Data.
(PDF)

S3 Fig Clustering analysis optimization and line graphs of the sum of intensities for each cluster. The cluster size, k, was varied using Perseus under the conditions described in Materials and methods, and the (A) average maximum and (B) minimum cluster size was determined for five applications of k-means clustering (error bars are SEM). Six clusters were considered optimal because of the peak in minimum cluster size and minor improvement (reduction) in maximum cluster size at k ≥ 6. Underlying data for this figure can be found in S1 Data.
(PDF)

S4 Fig Comparison of the number of significantly regulated phosphorylation sites for each protein versus membership in clusters and magnitude of regulation. (A) Graph of the number of significantly regulated phosphorylation sites for each protein from the analysis of activity-dependent phosphorylation in synaptosomes versus the number of clusters of which each protein is a member. Underlying data for this figure can be found in S1 Data. (B) Graph of the number of significantly regulated phosphorylation sites for each protein versus the number of clusters of which each protein is a member multiplied by the highest magnitude log_2_(stimulated intensity/control intensity) value, at any time point for all phosphopeptides detected for that protein. Proteins with relative high numbers of regulated phosphorylation sites are labelled by their gene name. Proteins proposed to be signal integrators (S5A–S5F Fig) have blue labels. The data are the result of six independent experiments for each stimulation condition (20 mM and 76 mM KCl). Underlying data for this figure can be found in S1 Data.
(PDF)

S5 Fig Heat maps of quantitative data from synaptosomes alongside domain structures for proteins with high numbers of phosphorylation sites that correspond to multiple regulatory patterns. Log_2_(stimulated intensity/control intensity) is shown using the indicated scale, across time after 20 mM or 76 mM KCl stimulation. Domain structure information obtained from Pfam, using the canonical isoform, is displayed with accurate positions of phosphorylation sites. Note that phosphorylation sites may correspond to specific UniProt accessions, which do not match the sequence numbering of the canonical isoform (see S1 and S2 Tables). Quantitative data were required to have significant up-/down-regulation at ≥1 time point(s). Proteins: (A) piccolo and bassoon; (B) MAP1B and tau; (C) CLASP2; (D) synapsin 1, 2, and 3; (E) RIM1; and (F) SNIP (gene name: *Srcin1*). Data for synapsin 1 and bassoon are shown both here and in the associated article to allow comparison to related proteins. Corresponding human tau phosphorylation sites are listed for easy comparison to rat sites. The data are the result of six independent experiments for each stimulation condition (20 mM and 76 mM KCl). CLASP2, cytoplasmic linker–associated protein 2; MAP1B, microtubule-associated protein 1B; RIM1, Rab3-interacting molecule 1; SNIP, SNAP25-interacting protein.
(PDF)

S6 Fig Enrichment of protein kinase substrate motifs and KS scores for protein kinase classes. (A) Heat map of probability of phosphorylation site motif enrichment using motif-x applied to the clusters of phosphopeptide temporal regulation (small stylized line graphs). The colour scale for motif-x score is indicated. Motifs are grouped by four broad types: basophilic, acidophilic, proline-directed, and other. (B) Heat map of KS scores for protein kinase classes arranged by hierarchical clustering. The colour scale for the KS score is indicated. KS score, KinSwing score.
(PDF)

S7 Fig Potential CaMKIIα substrates and substrates of poststimulus down-regulated phosphorylation within a network of presynaptic proteins. (A) Representative western blots of CaMKIIα-pT286 and β-actin loading control for 76 mM KCl depolarized and repolarized synaptosomes (upper). A bar graph of the densitometry of the western blots after correction for loading is shown (middle). The intensities were normalized to the control/mock stimulation. The bar graph shows the mean and standard error of the mean of three independently replicated experiments. Each time point measurement for the same replicate is shown in the same colour (solid circles). Statistical significance was determined by one-way analysis of variance and Dunnett’s post hoc test (*P < 0.05). The heat map of log_2_(stimulated intensity/control intensity) for CaMKIIα phosphopeptides containing S275, T286, or T306 detected in synaptosomes or neurons after 76 mM KCl (lower), using the same colour scale in Fig 2B and S5A–S5F Fig. Underlying data for this figure can be found in S1 Data. (B) The identity of the phosphorylation sites most likely to be phosphorylated by CaMKIIα, PKAα, or PKCα from the proteins shown in Fig 2C. The substrate probability for these protein kinases is shown as a heat map, with the indicated colour scale, alongside the heat map of log_2_(stimulated intensity/control intensity) for the corresponding phosphopeptides. For data derived from multisite phosphorylated peptides, the nonrelevant phosphorylation site is shown in grey lettering. The synaptosome data are the result of six independent experiments for the 76 mM KCl stimulation condition. The cultured hippocampal neuron data are from three independent experiments using 76 mM KCl stimulation. Underlying data for this figure can be found in S1 Data. (C) A word cloud visualization of the cluster 3 poststimulus down-regulated phosphorylation from synaptosomes using gene names anchored to cellular component ontology (subcellular localization). The size of the gene name was scaled to the average poststimulus log_2_(stimulated intensity/control intensity) value after both 20 mM and 76 mM KCl stimulation. Localizations: (i) cytoskeleton and actin cytoskeleton, (ii) endosome, (iii) microtubule and microtubule organizing centre, (iv) synaptic vesicle, (v) active zone scaffold, (vi) voltage-gated potassium channel, (vii) voltage-gated calcium channel, (viii) SNARE complex, (ix) clathrin-coated pit, and (x) presynaptic membrane and membrane raft. (xi) Protein kinases and protein phosphatases, including regulatory subunits, with no specific localization are also shown. Gene names in blue letters have three or more responding phosphorylation sites. Experimentally verified protein interactions (STRING) are shown as red edges, i.e., connecting lines. CaMKIIα, Ca^2+^- and calmodulin-dependent protein kinase IIα; ns, not significant; PKAα, protein kinase Aα; PKCα, protein kinase Cα; SNARE, soluble N-ethylmaleimide-sensitive factor attachment protein receptor.
(PDF)

S8 Fig The substrates of low-magnitude down-regulated and low-magnitude up-regulated phosphorylation within a network of presynaptic proteins. A word cloud visualization of (A) cluster 5 low-magnitude down-regulated phosphorylation and (B) cluster 6 low-magnitude up-regulated phosphorylation, from synaptosomes, using gene names anchored to cellular component ontology (subcellular localization). The size of the gene name was scaled to the average poststimulus log_2_(stimulated intensity/control intensity) value after both 20 mM and 76 mM KCl stimulation. Localizations: (i) cytoskeleton and actin cytoskeleton, (ii) endosome, (iii) microtubule and microtubule organizing centre, (iv) synaptic vesicle, (v) active zone scaffold, (vi) voltage-gated potassium channel, (vii) voltage-gated calcium channel, (viii) SNARE complex, (ix) clathrin-coated pit, and (x) presynaptic membrane and membrane raft. (xi) Protein kinases and protein phosphatases, including regulatory subunits, with no specific localization are also shown. Gene names in blue letters have three or more responding phosphorylation sites. Experimentally verified protein interactions (STRING) are shown as red edges, i.e., connecting lines. The data are the result of six independent experiments for each stimulation condition (20 mM and 76 mM KCl). SNARE, soluble N-ethylmaleimide-sensitive factor attachment protein receptor.
(PDF)

S1 Table Tables of processed quantitative phosphoproteomics data from synaptosomes and hippocampal neurons, including KinSwing data. (XLSX)

S2 Table Peptide–protein accession mapping file. The tables show the phosphopeptides from the synaptosome and hippocampal neuron experiments and the best matching protein accessions.
(XLSX)

S1 Data Tables of numerical data used to generate figures. (XLSX)

S1 File Substrates of cluster 1 from the synaptosome phosphoproteomics data mapped onto a presynaptic interaction network. In this presynaptic protein interaction network, the proteins that were not phospho-regulated were not filtered from the network. The edges (connecting lines between proteins) were scaled with the experimentally determined interaction value from STRING, in the range zero to one, linearly from white to red, and the line thickness was similarly scaled.
(PPTX)

S2 File Substrates of cluster 2 from the synaptosome phosphoproteomics data mapped onto a presynaptic interaction network. In this presynaptic protein interaction network, the proteins that were not phospho-regulated were not filtered from the network. The edges (connecting lines between proteins) were scaled with the experimentally determined interaction value from STRING, in the range zero to one, linearly from white to red, and the line thickness was similarly scaled.
(PPTX)

S3 File Substrates of cluster 3 from the synaptosome phosphoproteomics data mapped onto a presynaptic interaction network. In this presynaptic protein interaction network, the proteins that were not phospho-regulated were not filtered from the network. The edges (connecting lines between proteins) were scaled with the experimentally determined interaction value from STRING, in the range zero to one, linearly from white to red, and the line thickness was similarly scaled.
(PPTX)

S4 File Substrates of cluster 4 from the synaptosome phosphoproteomics data mapped onto a presynaptic interaction network. In this presynaptic protein interaction network, the proteins that were not phospho-regulated were not filtered from the network. The edges (connecting lines between proteins) were scaled with the experimentally determined interaction value from STRING, in the range zero to one, linearly from white to red, and the line thickness was similarly scaled.
(PPTX)

S5 File Substrates of cluster 5 from the synaptosome phosphoproteomics data mapped onto a presynaptic interaction network. In this presynaptic protein interaction network, the proteins that were not phospho-regulated were not filtered from the network. The edges (connecting lines between proteins) were scaled with the experimentally determined interaction value from STRING, in the range zero to one, linearly from white to red, and the line thickness was similarly scaled.
(PPTX)

S6 File Substrates of cluster 6 from the synaptosome phosphoproteomics data mapped onto a presynaptic interaction network. In this presynaptic protein interaction network, the proteins that were not phospho-regulated were not filtered from the network. The edges (connecting lines between proteins) were scaled with the experimentally determined interaction value from STRING, in the range zero to one, linearly from white to red, and the line thickness was similarly scaled.
(PPTX)

## Abbreviations

AGC: protein kinase A, G, and C
AKT: protein kinase B
CaMK: Ca^2+^- and calmodulin-dependent protein kinase
CaMKL: CaMK-like
*Caskin1*: Ca^2+^- and calmodulin-dependent serine protein kinase-interacting protein 1
CDC7: cell division cycle 7-related
CDK: cyclin-dependent kinase
CGMC: CDK, GSK3, MAPK, and CLK
CK2: casein kinase 2
*Clasp2*: cytoplasmic linker–associated protein 2
DAPK: death-associated protein kinase
*Dlgap4*: disks large-associated protein 4
DMPK: myotonic dystrophy protein kinase
DYRK: dual-specificity tyrosine-regulated kinases
ELKS: protein rich in E, L, K, and S
ERK: extracellular signal–regulated kinase
*Exoc7*: exocyst complex component 7
GRK: G protein–coupled receptor kinase
GSK3: glycogen synthase kinase
GTPase: guanosine triphosphatase
IKK: I kappa B kinase
IRSp53: insulin receptor substrate p53
*Kcnma1*: calcium-activated potassium channel subunit alpha-1
KS score: KinSwing score
LRRK: leucine-rich repeat kinase
MAP1B: microtubule-associated protein 1B
MAPK: mitogen-activated protein kinase
MAPKAPK: MAPK-activated protein kinase
MTOR: mechanistic target of rapamycin
PCA: principle component analysis
PDK1: pyruvate dehydrogenase kinase 1
PIKK: phosphatidylinositol 3-kinase-related kinase
*Pip5k1c*: phosphatidylinositol 4-phosphate 5-kinase type-1 γ
PKA: protein kinase A
PKC: protein kinase C
PLK: polo-like kinase
PP1: protein phosphatase 1
PP2A: protein phosphatase 2A
RIM: Rab3-interacting molecule
RSK: ribosomal S6 kinase 1
*Sept7*: Septin 7
*Shank3*: SH3 and multiple ankyrin repeat domains protein 3
*Snph*: syntaphilin
STE: sterile
*Sybu*: syntabulin
TKL: tyrosine kinase-like
