# Supplementary figures and images for: The temporal profile of activity-dependent presynaptic phospho-signalling reveals long-lasting patterns of poststimulus regulation
Source: PLoS Biol. 2019 Mar 1;17(3):e3000170. doi: 10.1371/journal.pbio.3000170 (PMC6415872; doi:10.1371/journal.pbio.3000170)

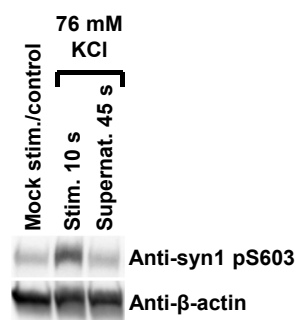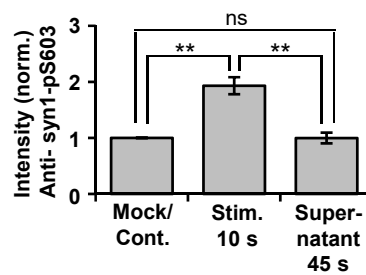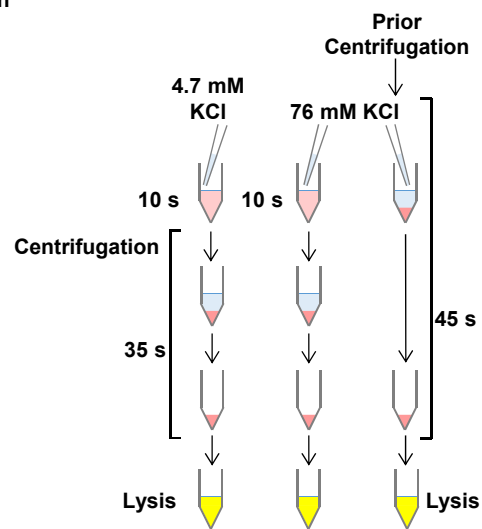

Supplement: S1 Fig — The switch from 20 or 76 mM KCl to 4.7 mM KCl required a centrifugation step and manual exchange of the solution, which was a 35-s (±1 s, standard deviation; n = 9) process, using our equipment. If Ca2+ could continue to enter the pelleted synaptosomes during the exchange process, then our stimulation would be longer than intended. We investigated the potential for KCl to influence pelleted synaptosomes. We used up-regulation of synapsin 1 S603 as a marker of depolarization. We compared standard stimulation with elevated KCl—i.e., 10-s 76 mM KCl stimulation, centrifugation, and manual solution exchange (35 s) to a 45-s stimulation of the supernatant after centrifugation. This latter condition was used to test if elevated KCl could stimulate/depolarize the pelleted synaptosomes. Volumes and ionic strength were consistent throughout (see Materials and methods). Left: representative western blot of the comparison of 4.7 mM KCl mock treatment and 76 mM KCl depolarizing treatment of suspended synaptosomes alongside a 76 mM KCl treatment of the supernatant of pelleted synaptosomes. Successful depolarization (positive control) was confirmed by western blot with anti-syn1-pS603. Anti-β-actin was used as a loading control. Right: quantification of the western blotting. Bar graph of the average and SEM of three independent experiments (normalized to mock-treated/control sample, one-way analysis of variance and Dunnett’s post hoc test; **P < 0.01). Below: schematic of the experimental procedure for each condition in the bar graph. The failure of high-concentration KCl in the supernatant to up-regulate pS603 allowed us to conclude that KCl present during the solution exchange is unlikely to influence phospho-signalling in pelleted synaptosomes. Thus, our 10-s stimulation paradigm was established as an acute stimulation. Underlying data for this figure can be found in S1 Data. (PDF) [file pbio.3000170.s001.pdf]

**A**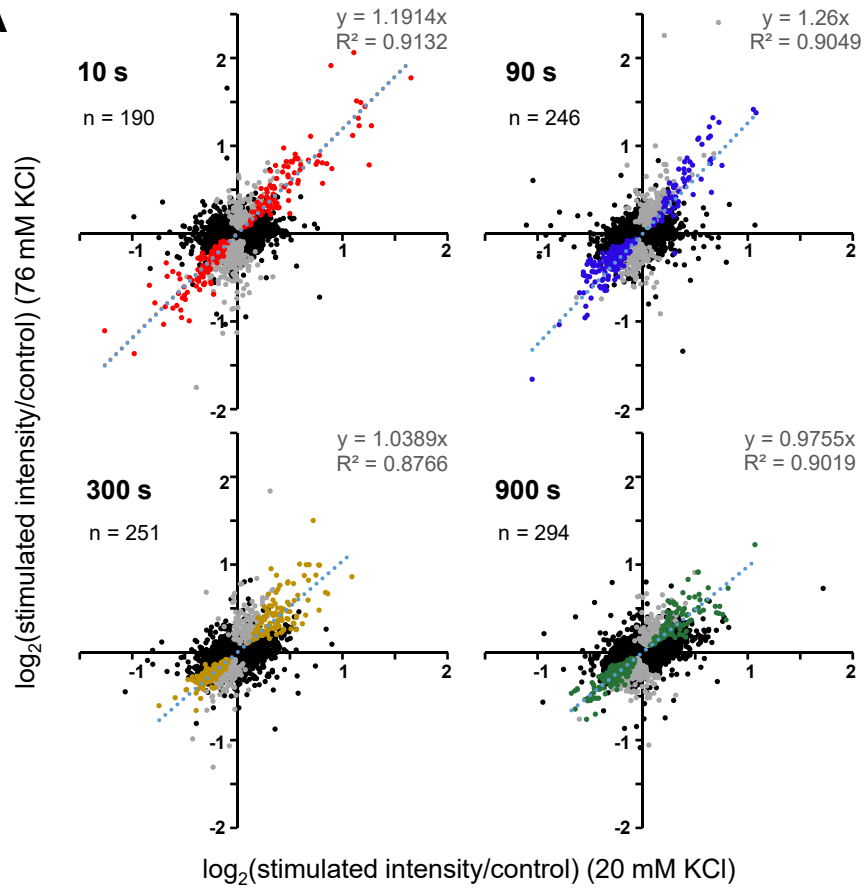**B**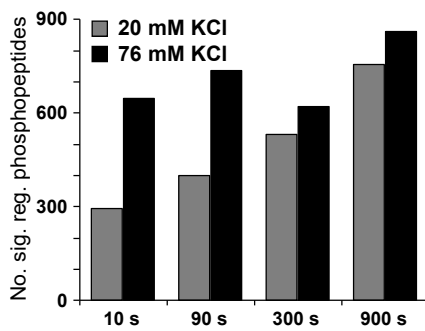**C**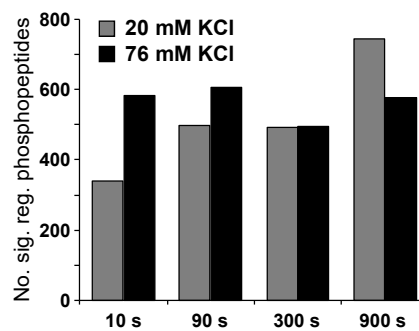**D**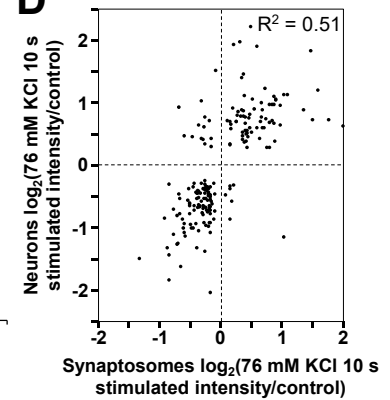

Supplement: S2 Fig — (A) Plot of 20 mM KCl versus 76 mM KCl log2(stimulated intensity/control intensity) phosphorylation level changes at 10, 90, 300, and 900 s. The significant phosphorylation level changes detected in both 20 mM and 76 mM KCl experiments are shown in colour (the number, n, of intersecting data points for 20 mM and 76 mM KCl is shown for each time point). Those significant only after 76 mM KCl stimulation are shown in grey. Nonsignificant values are shown in black. The linear fit of significant values for each plot are shown (fitted line was required to pass through zero). Underlying data for this figure can be found in S1 Data. (B) Count of significantly regulated phosphopeptides after 20 mM (grey bars) and 76 mM KCl (black bars) stimulation for each time point. Phosphopeptides were not required to be detected in both stimulation level experiments. Underlying data for this figure can be found in S1 Data. (C) This figure differs from (B) because of the requirement that phosphopeptide signals were detected in both the 20 mM and 76 mM KCl stimulation experiments. Underlying data for this figure can be found in S1 Data. (D) Plot of log2(76 mM KCl 10-s stimulated intensity/control intensity) for phosphopeptides from cultured hippocampal neurons versus whole-brain synaptosomes. The plot includes only the 195 phosphopeptides that were significantly regulated in both types of samples. The synaptosome data are the result of six independent experiments for each stimulation condition (20 mM and 76 mM KCl). The cultured hippocampal neuron data are from three independent experiments using 76 mM KCl stimulation. A moderated t-statistic adjusted for multiple hypothesis testing was used to determine the significance of single time points, P < 0.05. Underlying data for this figure can be found in S1 Data. (PDF) [file pbio.3000170.s002.pdf]

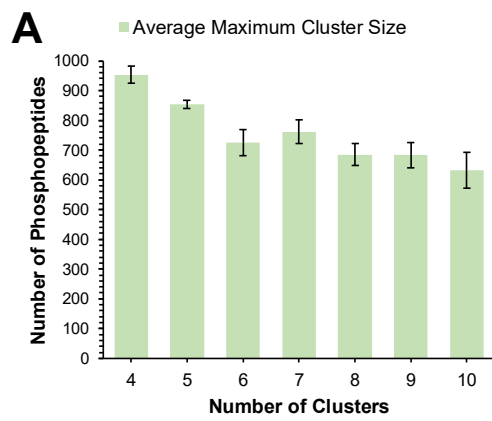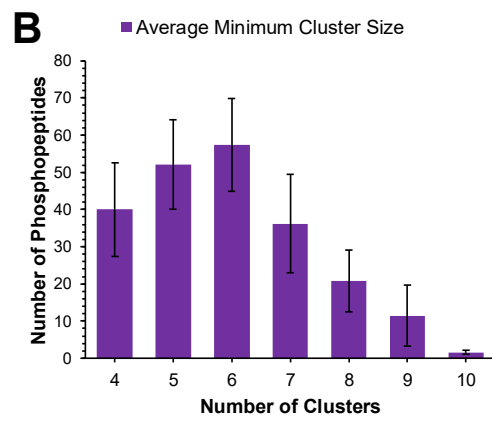

Supplement: S3 Fig — The cluster size, k, was varied using Perseus under the conditions described in Materials and methods, and the (A) average maximum and (B) minimum cluster size was determined for five applications of k-means clustering (error bars are SEM). Six clusters were considered optimal because of the peak in minimum cluster size and minor improvement (reduction) in maximum cluster size at k ≥ 6. Underlying data for this figure can be found in S1 Data. (PDF) [file pbio.3000170.s003.pdf]

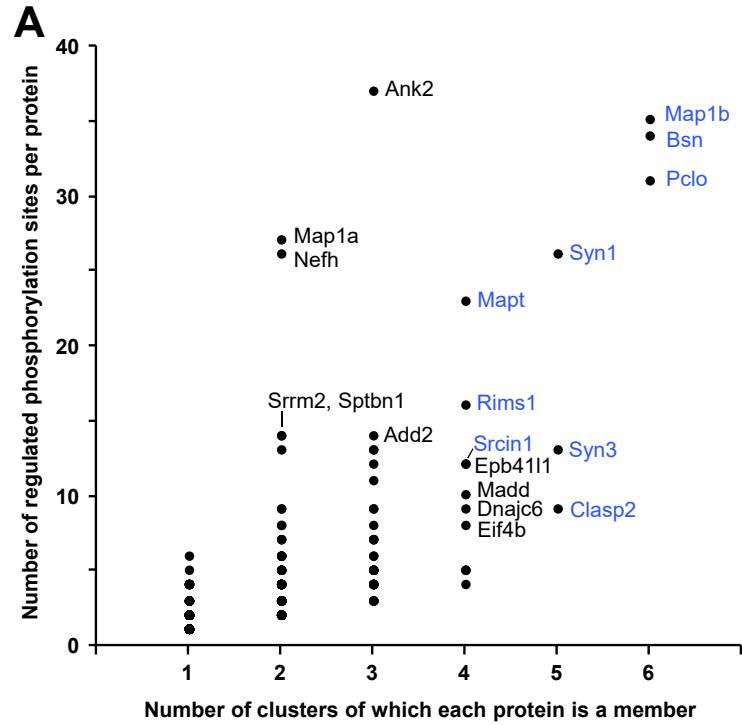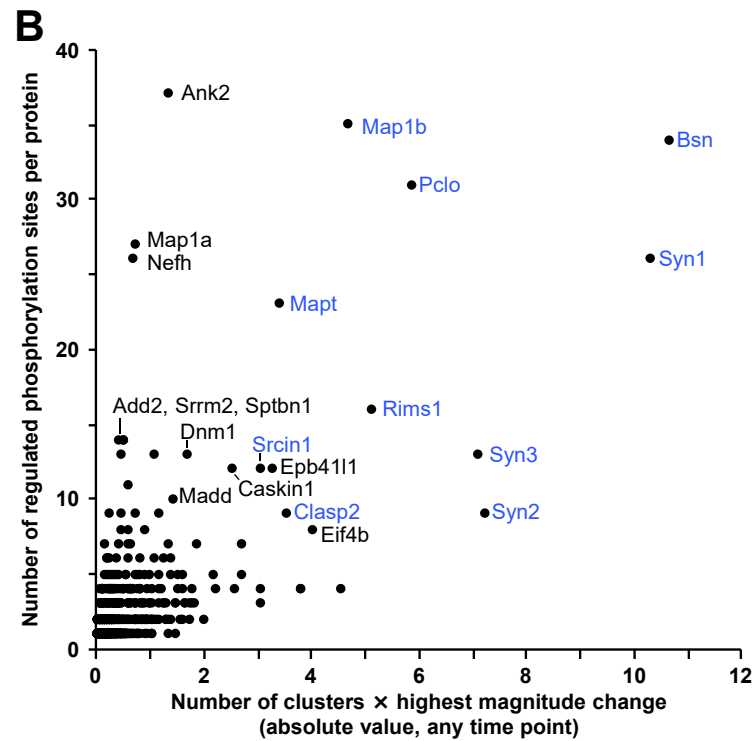

Supplement: S4 Fig — (A) Graph of the number of significantly regulated phosphorylation sites for each protein from the analysis of activity-dependent phosphorylation in synaptosomes versus the number of clusters of which each protein is a member. Underlying data for this figure can be found in S1 Data. (B) Graph of the number of significantly regulated phosphorylation sites for each protein versus the number of clusters of which each protein is a member multiplied by the highest magnitude log2(stimulated intensity/control intensity) value, at any time point for all phosphopeptides detected for that protein. Proteins with relative high numbers of regulated phosphorylation sites are labelled by their gene name. Proteins proposed to be signal integrators (S5A–S5F Fig) have blue labels. The data are the result of six independent experiments for each stimulation condition (20 mM and 76 mM KCl). Underlying data for this figure can be found in S1 Data. (PDF) [file pbio.3000170.s004.pdf]

**A**

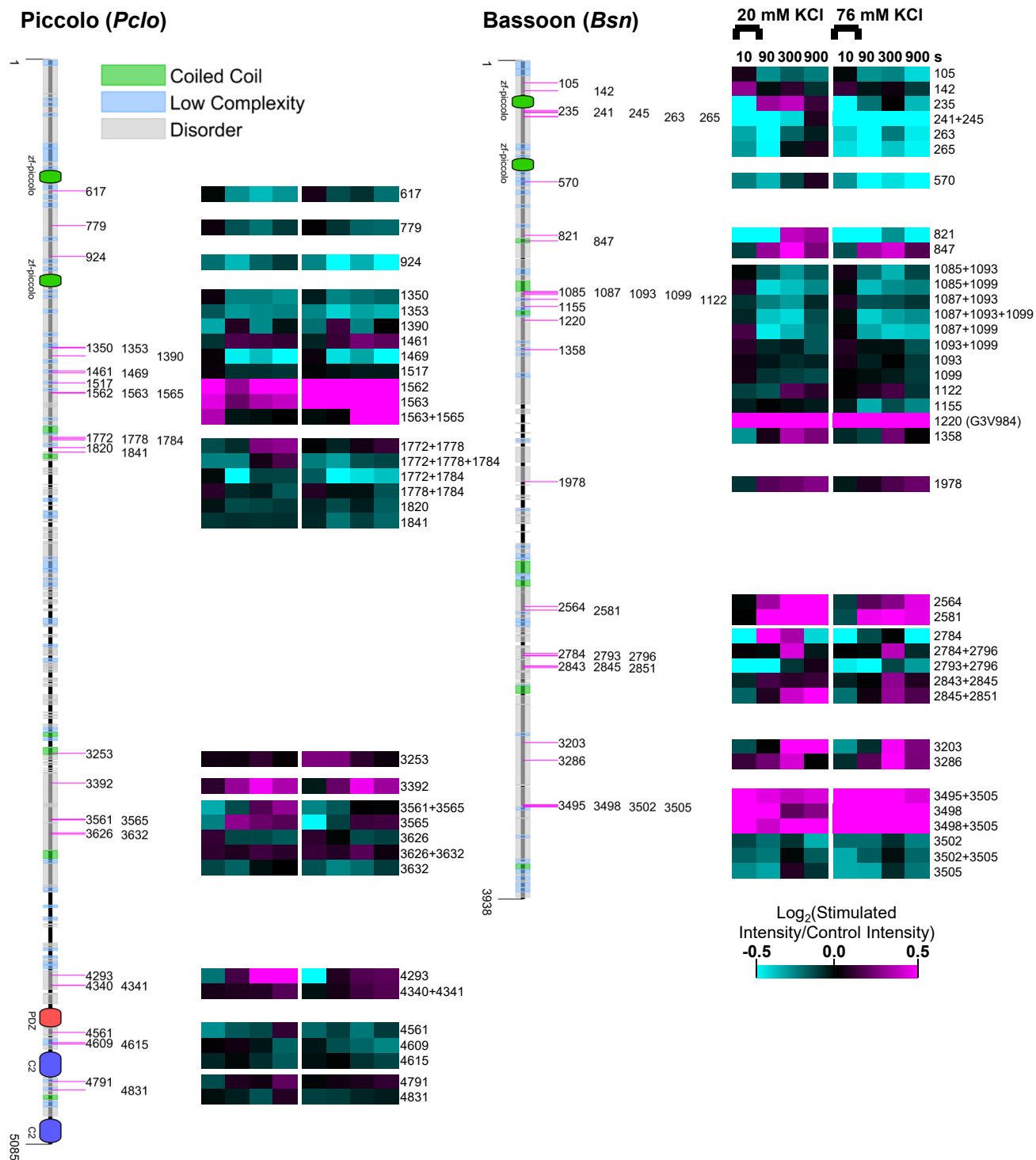

B

MAP1B (*Map1b*)

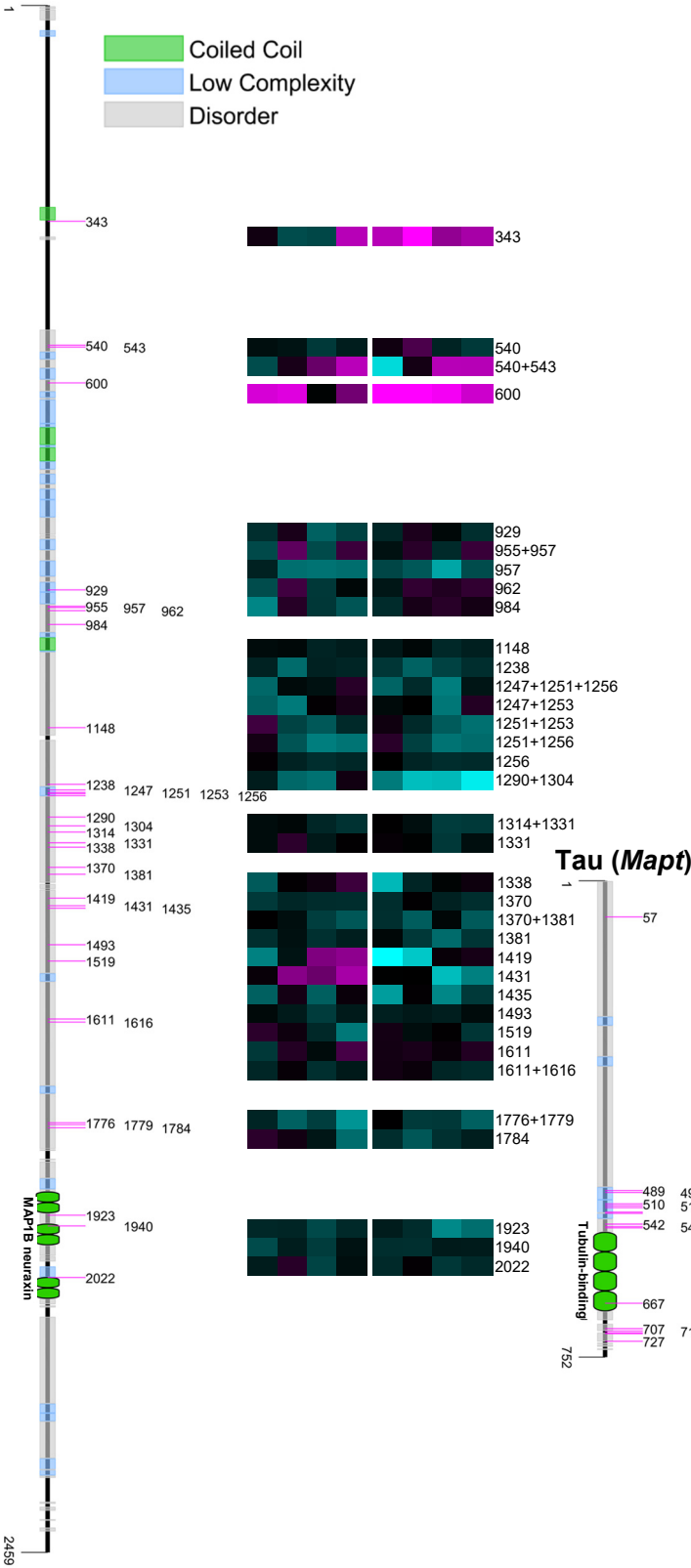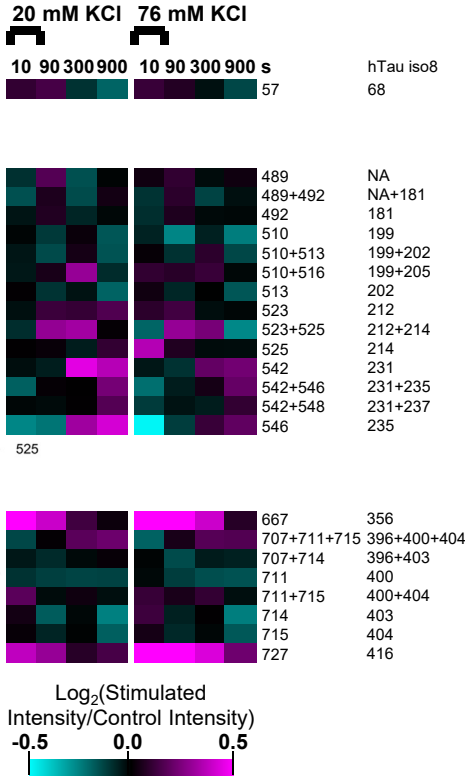

C

- Coiled Coil
- Low Complexity
- Disorder

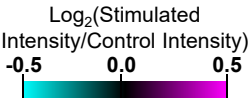

CLASP2 (*Clasp2*)

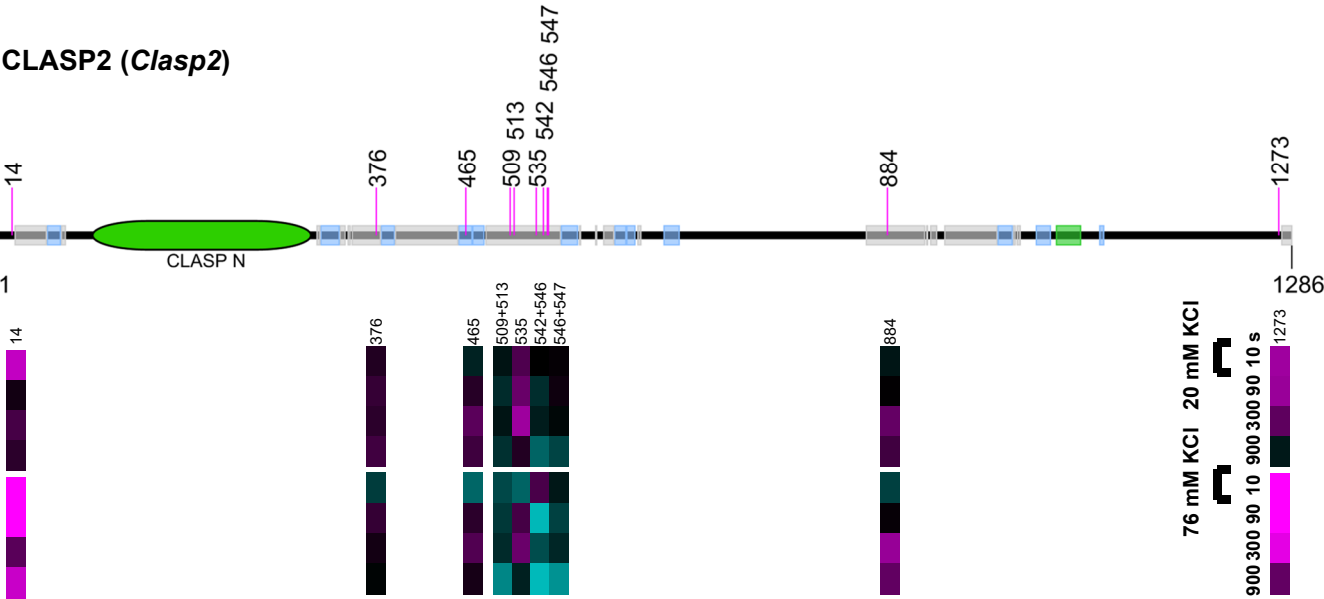

**D**

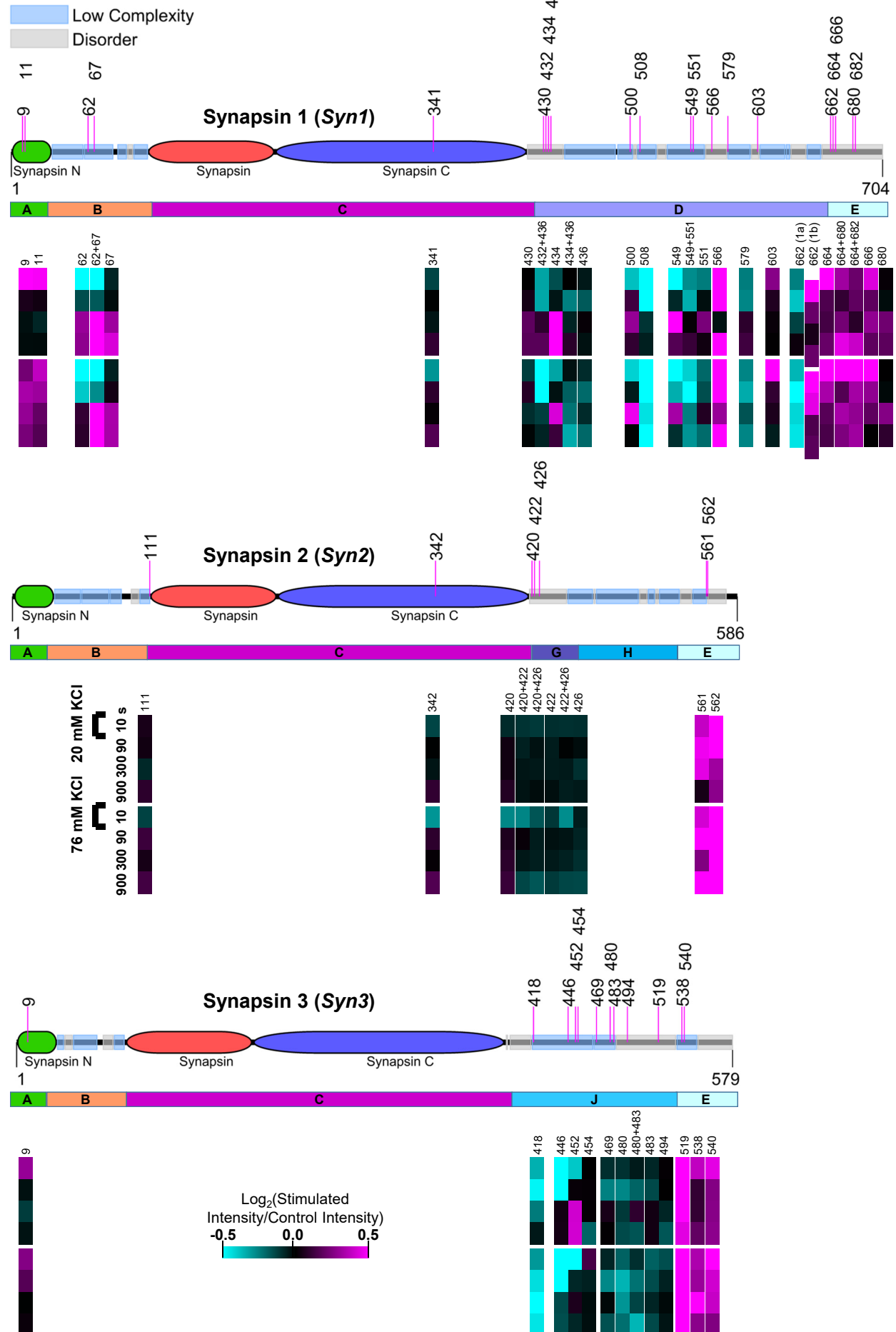

E

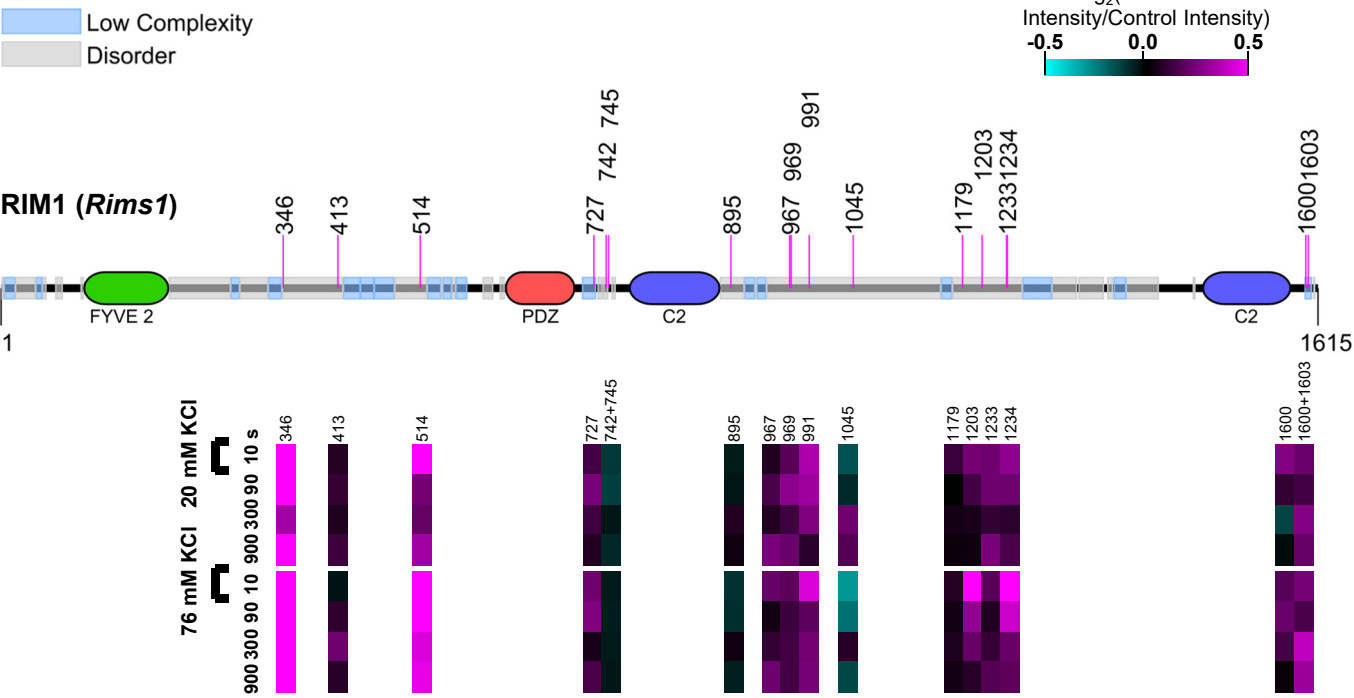

## F

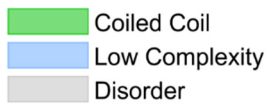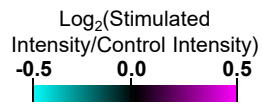

## SNIP (*Srcn1*)

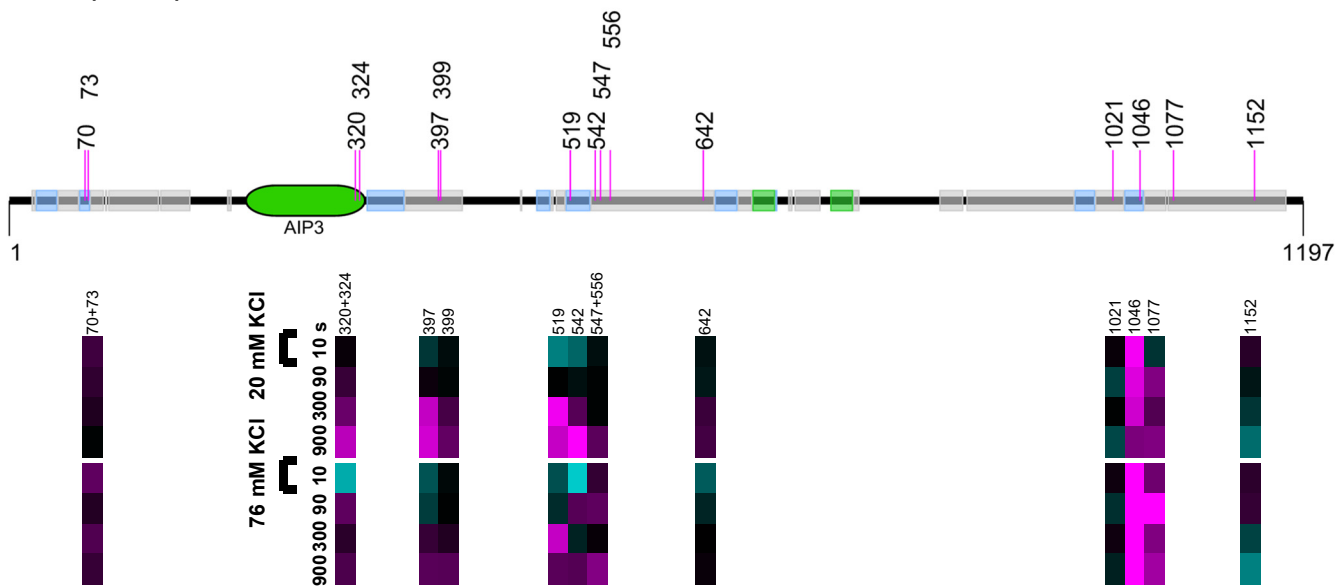

Supplement: S5 Fig — Log2(stimulated intensity/control intensity) is shown using the indicated scale, across time after 20 mM or 76 mM KCl stimulation. Domain structure information obtained from Pfam, using the canonical isoform, is displayed with accurate positions of phosphorylation sites. Note that phosphorylation sites may correspond to specific UniProt accessions, which do not match the sequence numbering of the canonical isoform (see S1 and S2 Tables). Quantitative data were required to have significant up-/down-regulation at ≥1 time point(s). Proteins: (A) piccolo and bassoon; (B) MAP1B and tau; (C) CLASP2; (D) synapsin 1, 2, and 3; (E) RIM1; and (F) SNIP (gene name: Srcin1). Data for synapsin 1 and bassoon are shown both here and in the associated article to allow comparison to related proteins. Corresponding human tau phosphorylation sites are listed for easy comparison to rat sites. The data are the result of six independent experiments for each stimulation condition (20 mM and 76 mM KCl). CLASP2, cytoplasmic linker–associated protein 2; MAP1B, microtubule-associated protein 1B; RIM1, Rab3-interacting molecule 1; SNIP, SNAP25-interacting protein. (PDF) [file pbio.3000170.s005.pdf]

**A**

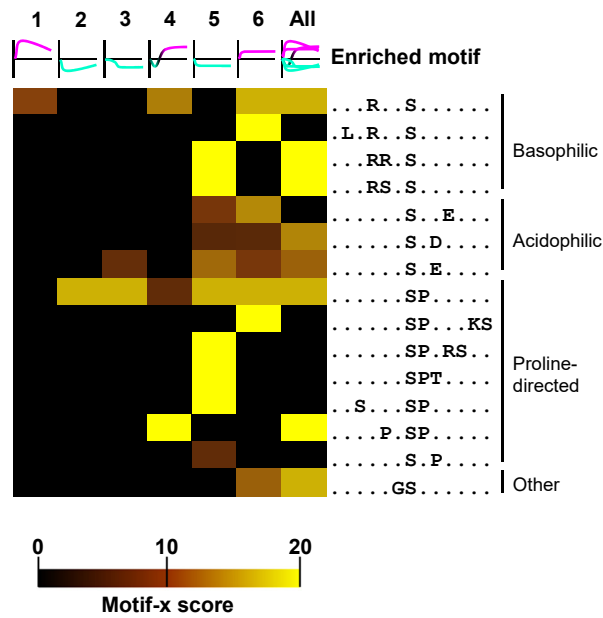

**B**

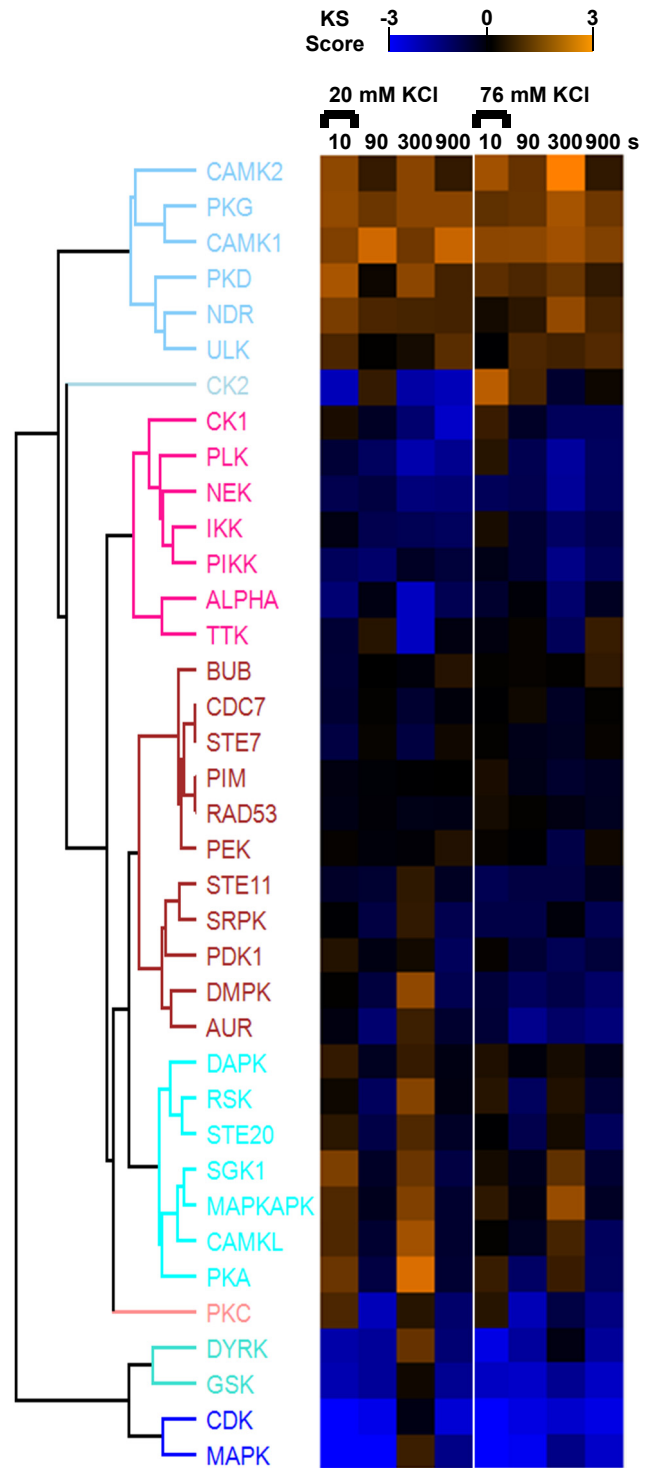

Supplement: S6 Fig — (A) Heat map of probability of phosphorylation site motif enrichment using motif-x applied to the clusters of phosphopeptide temporal regulation (small stylized line graphs). The colour scale for motif-x score is indicated. Motifs are grouped by four broad types: basophilic, acidophilic, proline-directed, and other. (B) Heat map of KS scores for protein kinase classes arranged by hierarchical clustering. The colour scale for the KS score is indicated. KS score, KinSwing score. (PDF) [file pbio.3000170.s006.pdf]

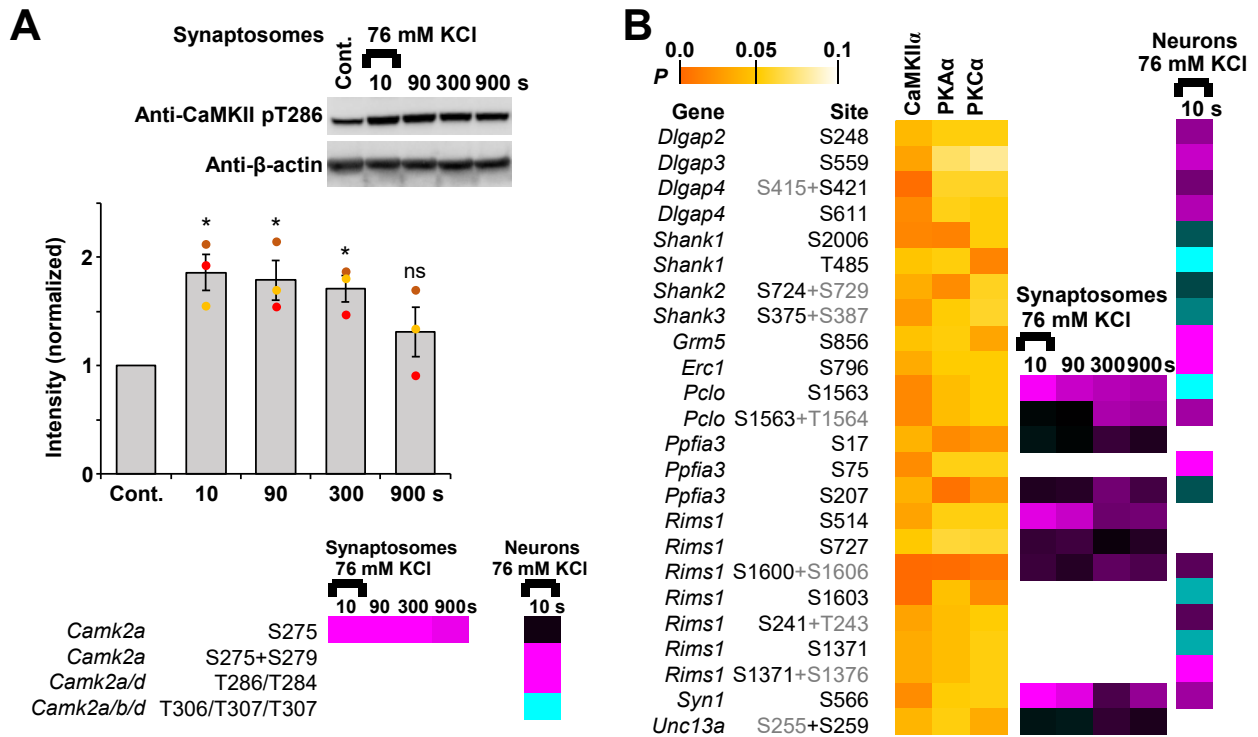

**C Cluster 3 poststimulus down-regulated phosphorylation**

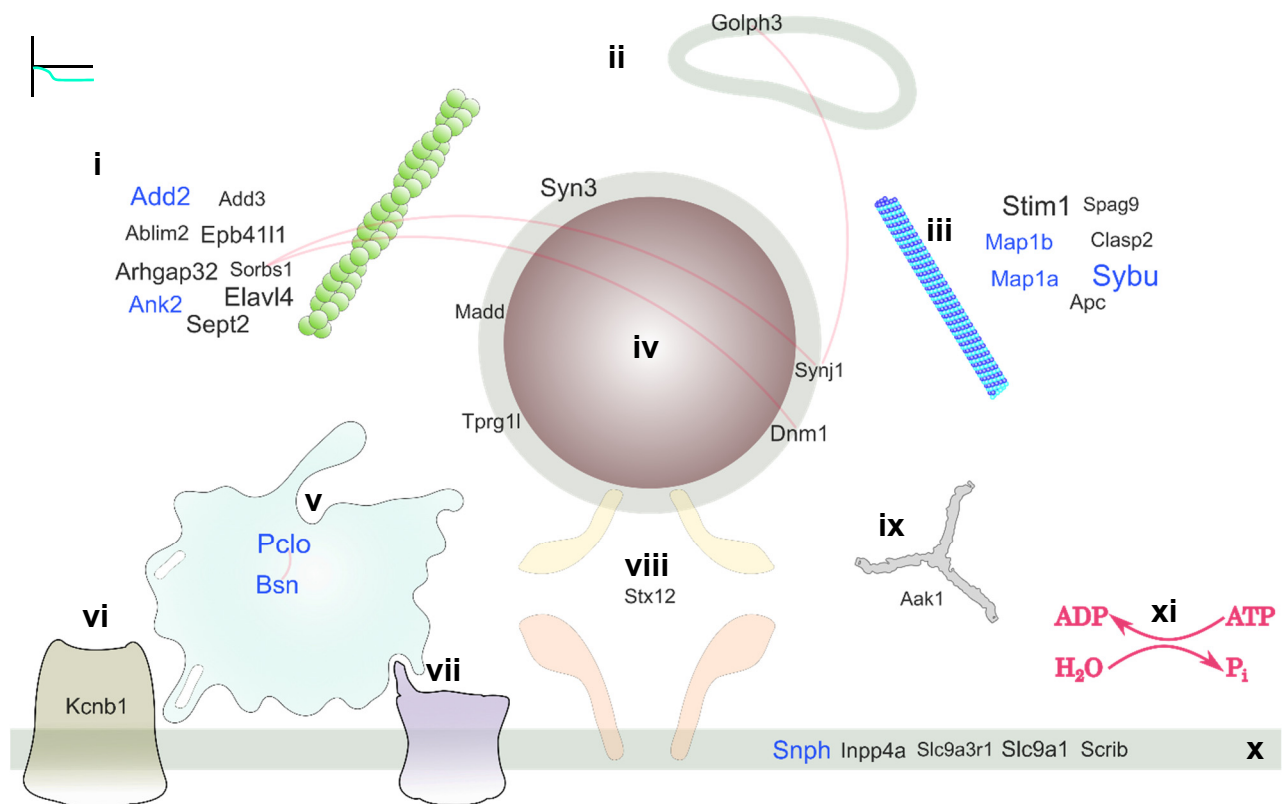

Supplement: S7 Fig — (A) Representative western blots of CaMKIIα-pT286 and β-actin loading control for 76 mM KCl depolarized and repolarized synaptosomes (upper). A bar graph of the densitometry of the western blots after correction for loading is shown (middle). The intensities were normalized to the control/mock stimulation. The bar graph shows the mean and standard error of the mean of three independently replicated experiments. Each time point measurement for the same replicate is shown in the same colour (solid circles). Statistical significance was determined by one-way analysis of variance and Dunnett’s post hoc test (*P < 0.05). The heat map of log2(stimulated intensity/control intensity) for CaMKIIα phosphopeptides containing S275, T286, or T306 detected in synaptosomes or neurons after 76 mM KCl (lower), using the same colour scale in Fig 2B and S5A–S5F Fig. Underlying data for this figure can be found in S1 Data. (B) The identity of the phosphorylation sites most likely to be phosphorylated by CaMKIIα, PKAα, or PKCα from the proteins shown in Fig 2C. The substrate probability for these protein kinases is shown as a heat map, with the indicated colour scale, alongside the heat map of log2(stimulated intensity/control intensity) for the corresponding phosphopeptides. For data derived from multisite phosphorylated peptides, the nonrelevant phosphorylation site is shown in grey lettering. The synaptosome data are the result of six independent experiments for the 76 mM KCl stimulation condition. The cultured hippocampal neuron data are from three independent experiments using 76 mM KCl stimulation. Underlying data for this figure can be found in S1 Data. (C) A word cloud visualization of the cluster 3 poststimulus down-regulated phosphorylation from synaptosomes using gene names anchored to cellular component ontology (subcellular localization). The size of the gene name was scaled to the average poststimulus log2(stimulated intensity/control intensity) value after both 20 mM and [file pbio.3000170.s007.pdf]

## Slide 1
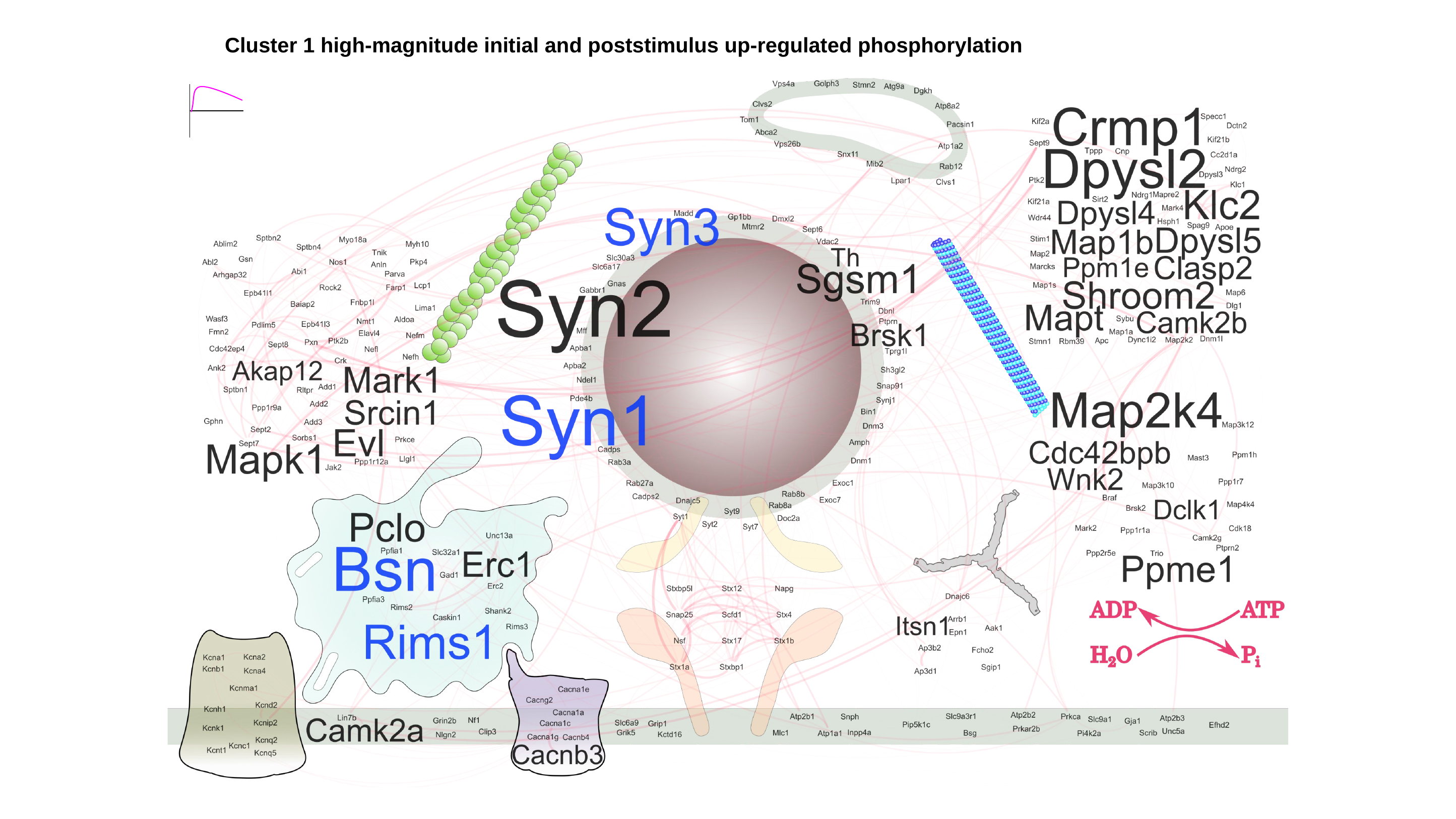

Cluster 1 high-magnitude initial and poststimulus up-regulated phosphorylation

Supplement: S1 File — In this presynaptic protein interaction network, the proteins that were not phospho-regulated were not filtered from the network. The edges (connecting lines between proteins) were scaled with the experimentally determined interaction value from STRING, in the range zero to one, linearly from white to red, and the line thickness was similarly scaled. (PPTX) [file pbio.3000170.s012.pptx]

## Slide 1
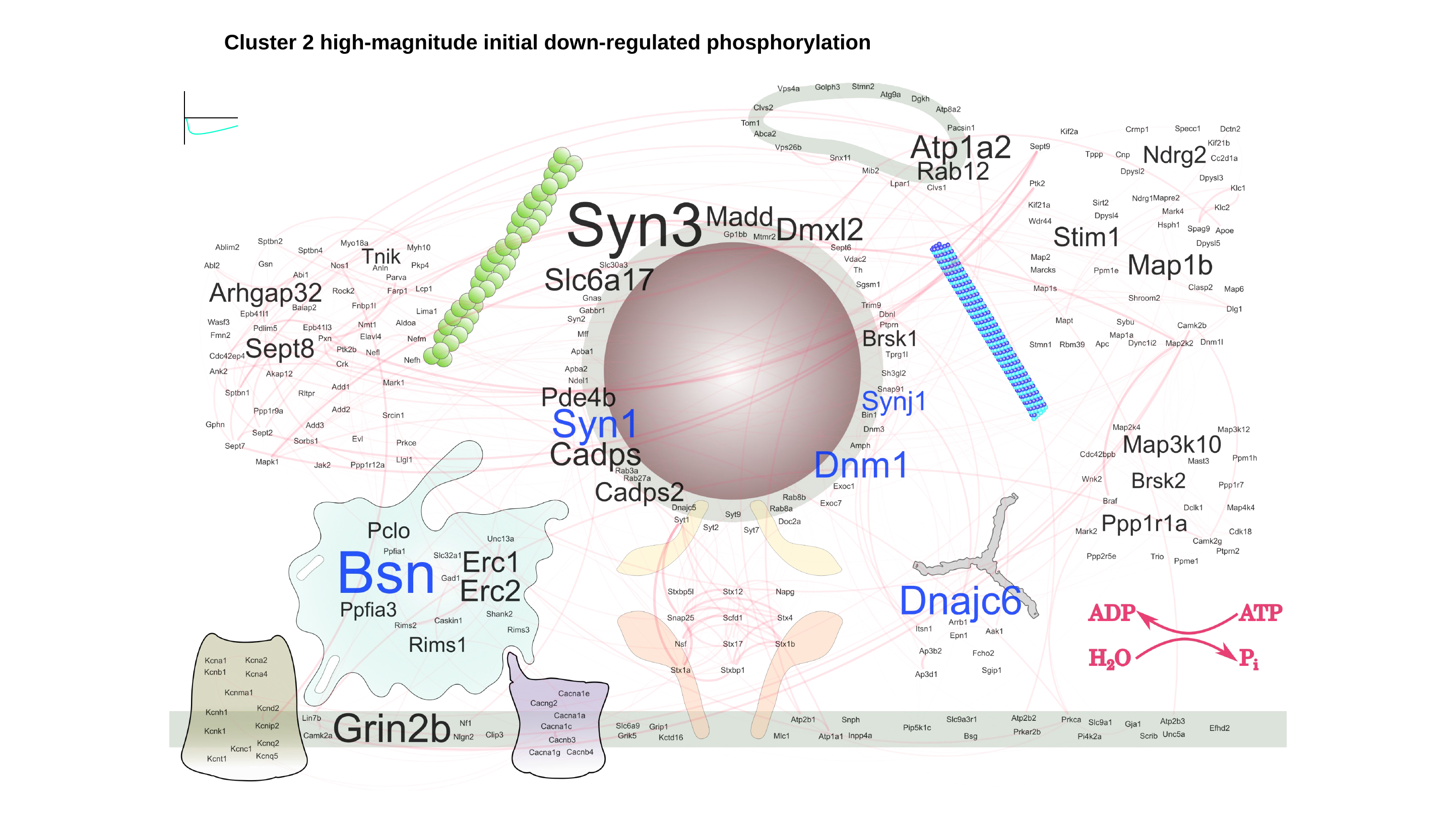

Cluster 2 high-magnitude initial down-regulated phosphorylation

Supplement: S2 File — In this presynaptic protein interaction network, the proteins that were not phospho-regulated were not filtered from the network. The edges (connecting lines between proteins) were scaled with the experimentally determined interaction value from STRING, in the range zero to one, linearly from white to red, and the line thickness was similarly scaled. (PPTX) [file pbio.3000170.s013.pptx]

## Slide 1
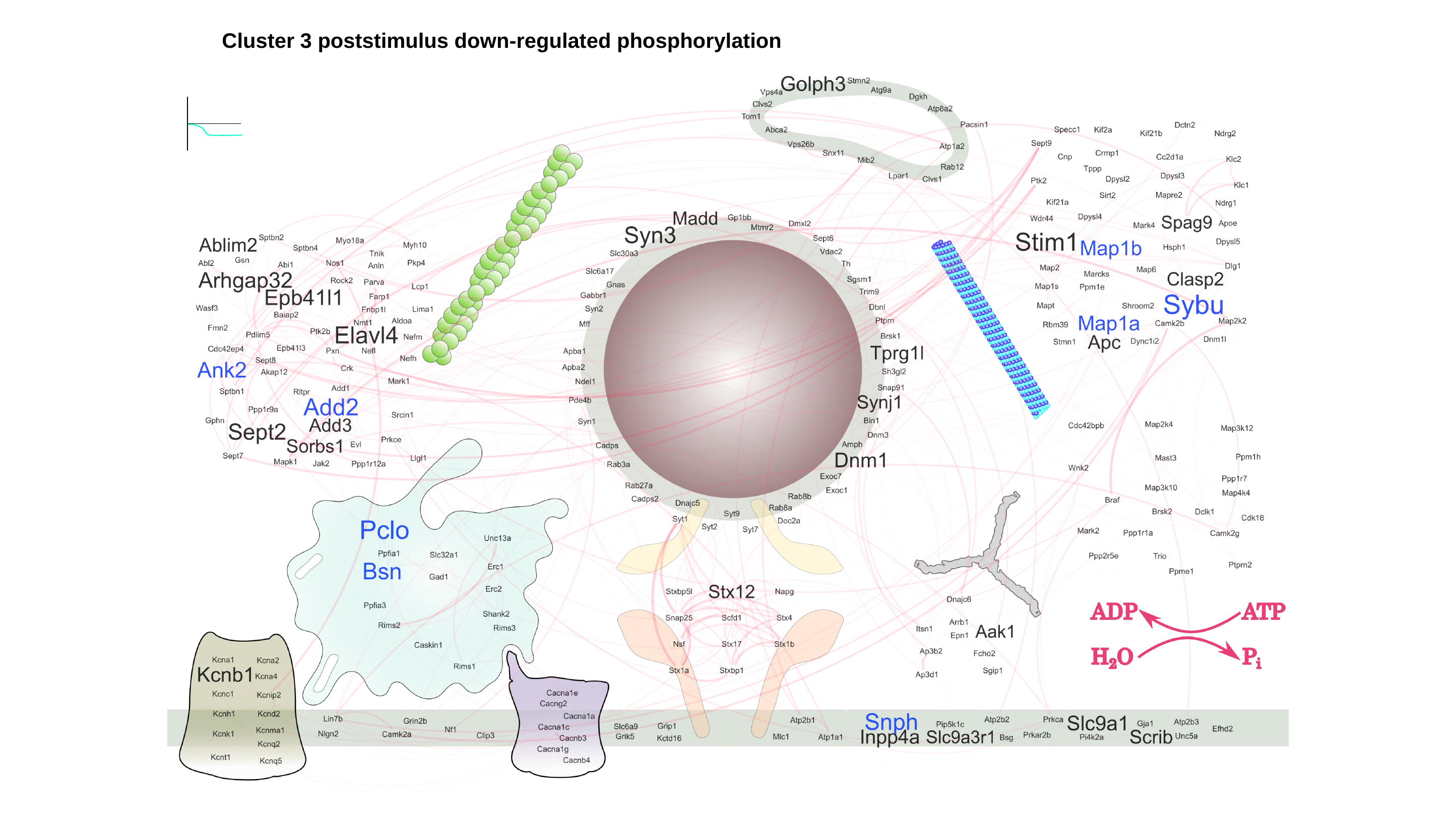

Cluster 3 poststimulus down-regulated phosphorylation

Supplement: S3 File — In this presynaptic protein interaction network, the proteins that were not phospho-regulated were not filtered from the network. The edges (connecting lines between proteins) were scaled with the experimentally determined interaction value from STRING, in the range zero to one, linearly from white to red, and the line thickness was similarly scaled. (PPTX) [file pbio.3000170.s014.pptx]

## Slide 1
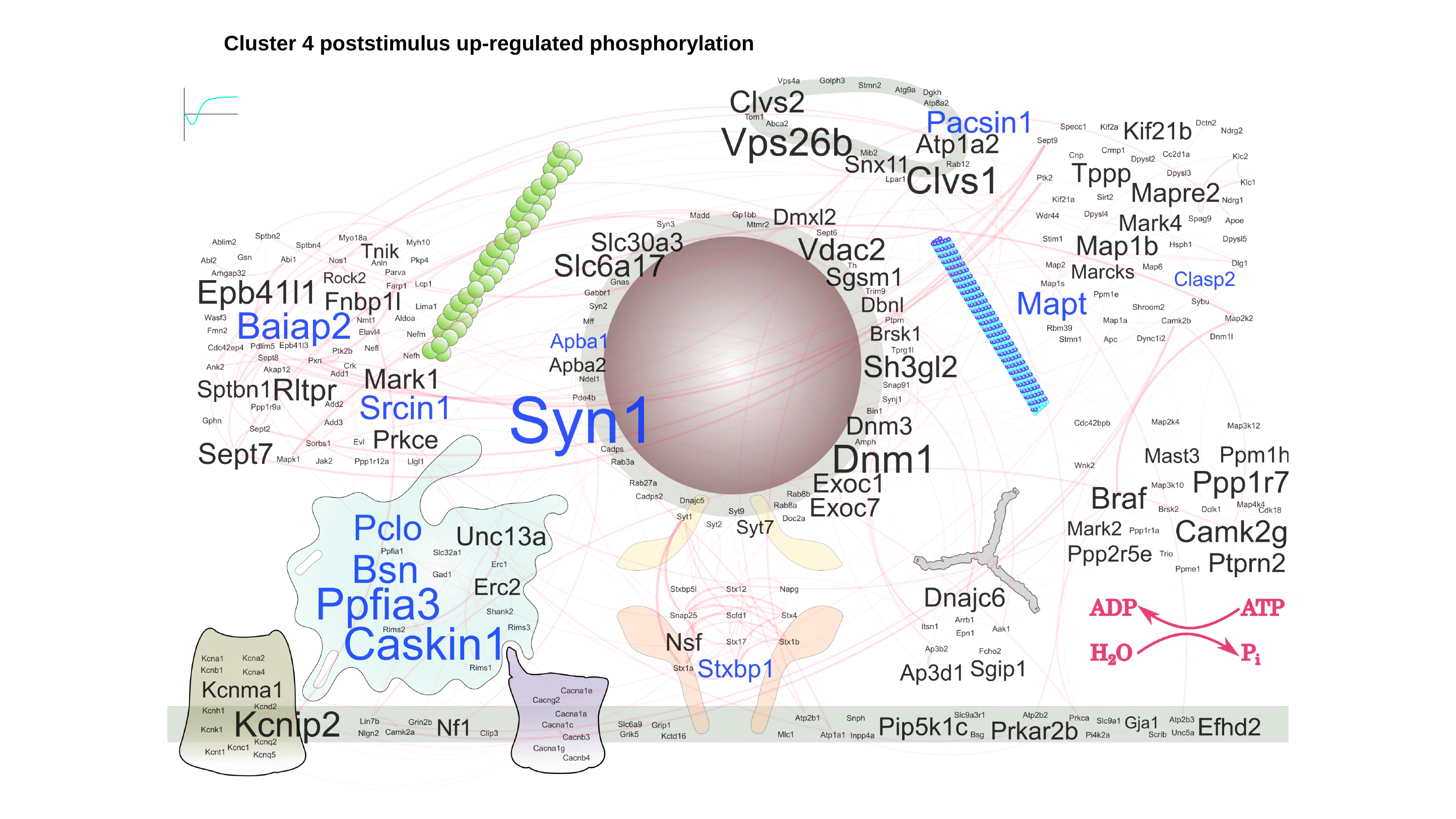

Cluster 4 poststimulus up-regulated phosphorylation

Supplement: S4 File — In this presynaptic protein interaction network, the proteins that were not phospho-regulated were not filtered from the network. The edges (connecting lines between proteins) were scaled with the experimentally determined interaction value from STRING, in the range zero to one, linearly from white to red, and the line thickness was similarly scaled. (PPTX) [file pbio.3000170.s015.pptx]

## Slide 1
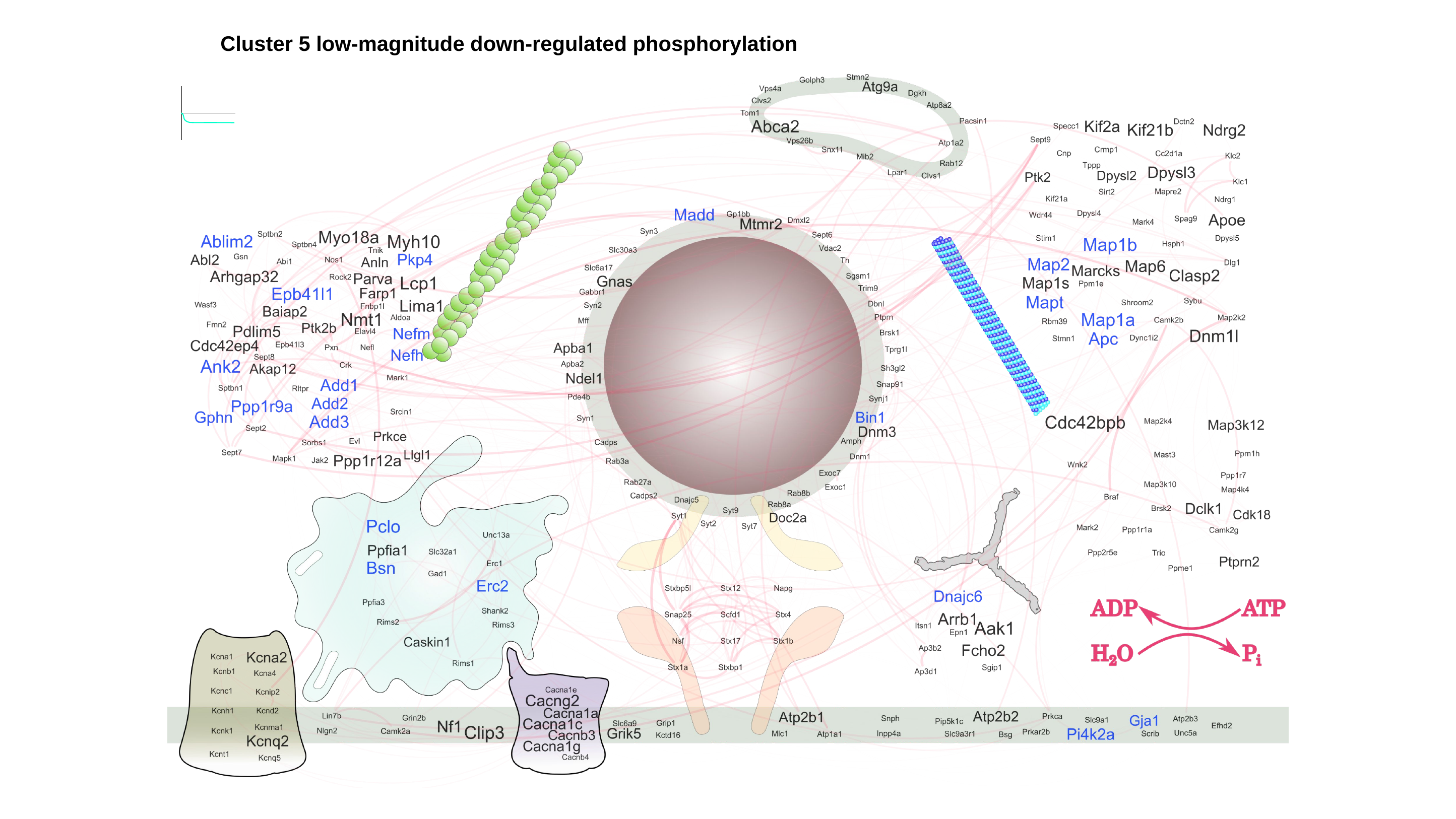

Cluster 5 low-magnitude down-regulated phosphorylation

Supplement: S5 File — In this presynaptic protein interaction network, the proteins that were not phospho-regulated were not filtered from the network. The edges (connecting lines between proteins) were scaled with the experimentally determined interaction value from STRING, in the range zero to one, linearly from white to red, and the line thickness was similarly scaled. (PPTX) [file pbio.3000170.s016.pptx]

## Slide 1
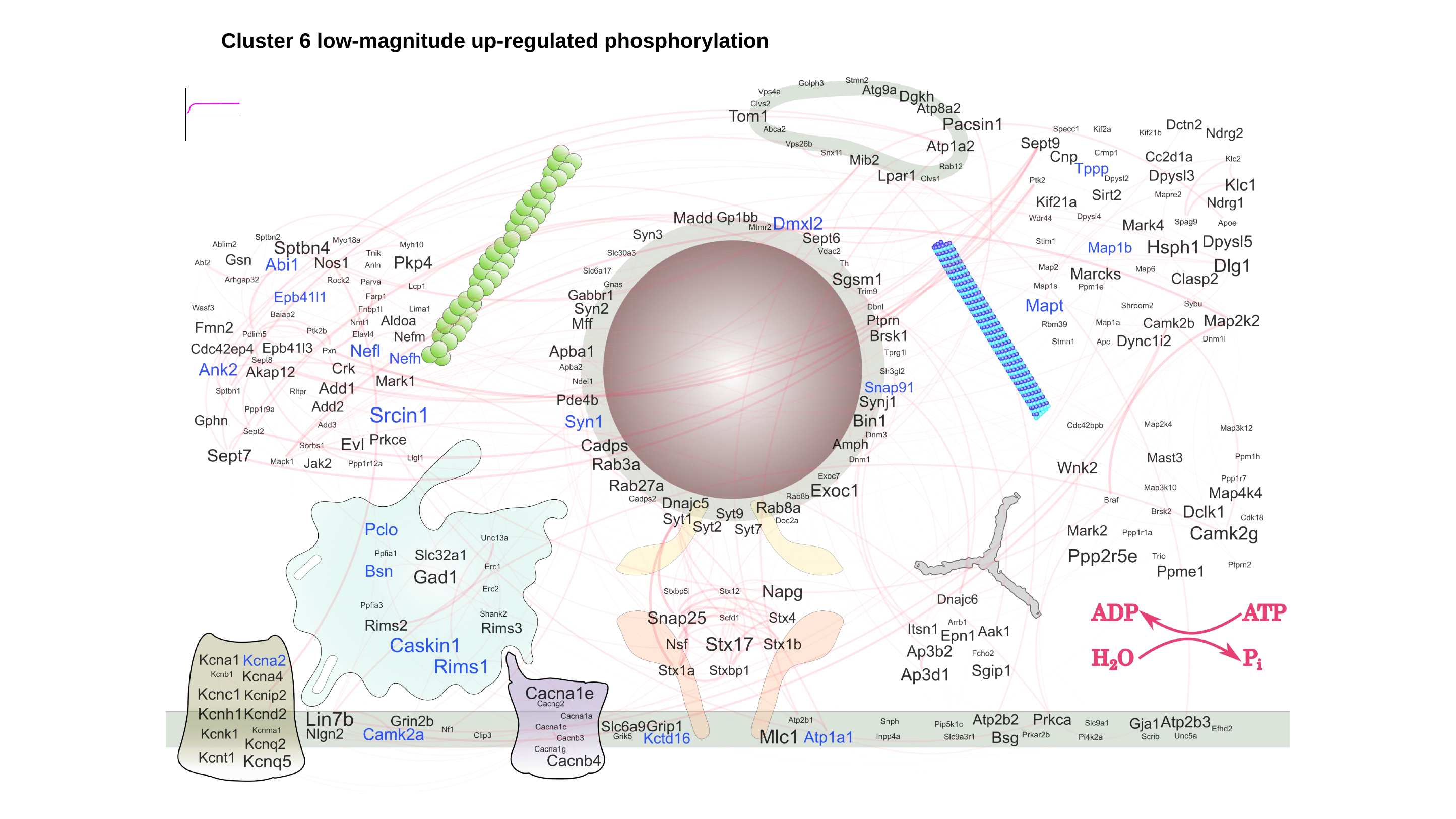

Cluster 6 low-magnitude up-regulated phosphorylation

Supplement: S6 File — In this presynaptic protein interaction network, the proteins that were not phospho-regulated were not filtered from the network. The edges (connecting lines between proteins) were scaled with the experimentally determined interaction value from STRING, in the range zero to one, linearly from white to red, and the line thickness was similarly scaled. (PPTX) [file pbio.3000170.s017.pptx]
