# Supplementary material for: The temporal profile of activity-dependent presynaptic phospho-signalling reveals long-lasting patterns of poststimulus regulation
Source: PLoS Biol. 2019 Mar 1;17(3):e3000170. doi: 10.1371/journal.pbio.3000170 (PMC6415872; doi:10.1371/journal.pbio.3000170)

A **Cluster 5 low-magnitude down-regulated phosphorylation**

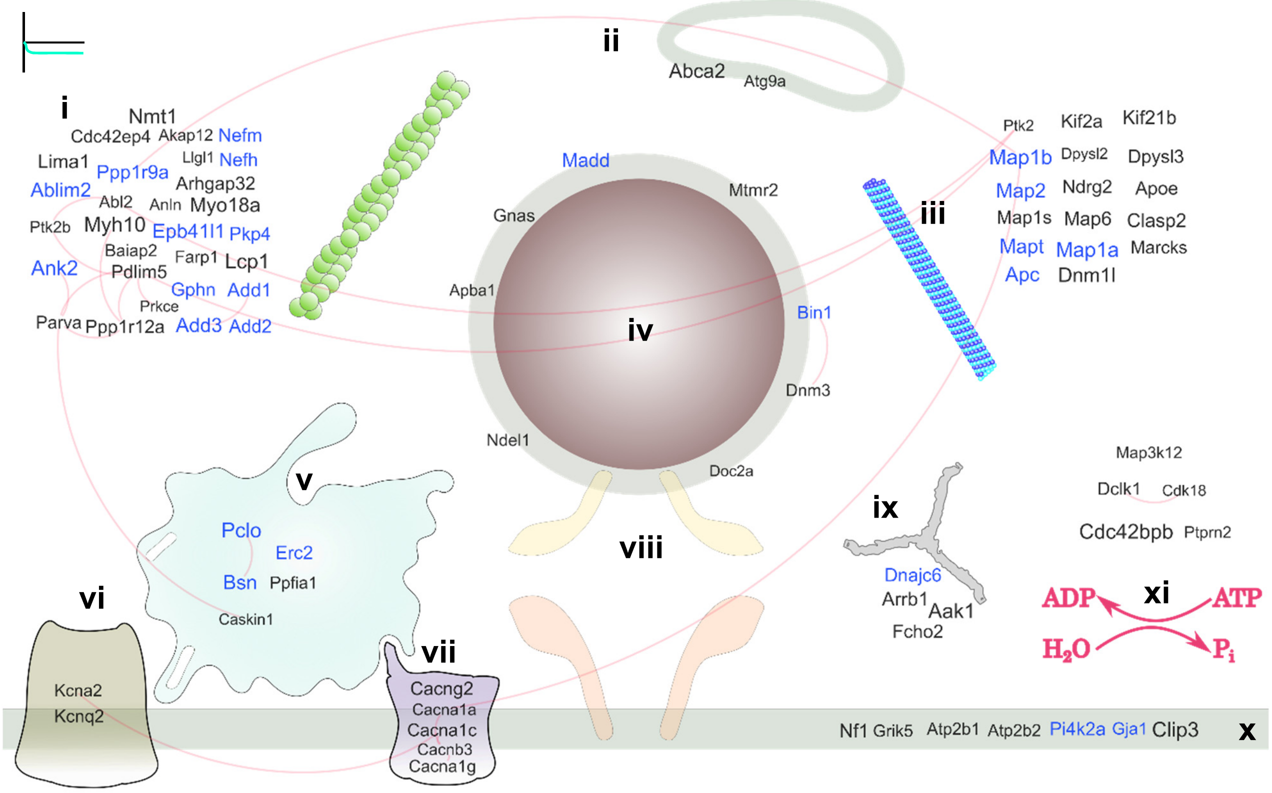

B **Cluster 6 low-magnitude up-regulated phosphorylation**

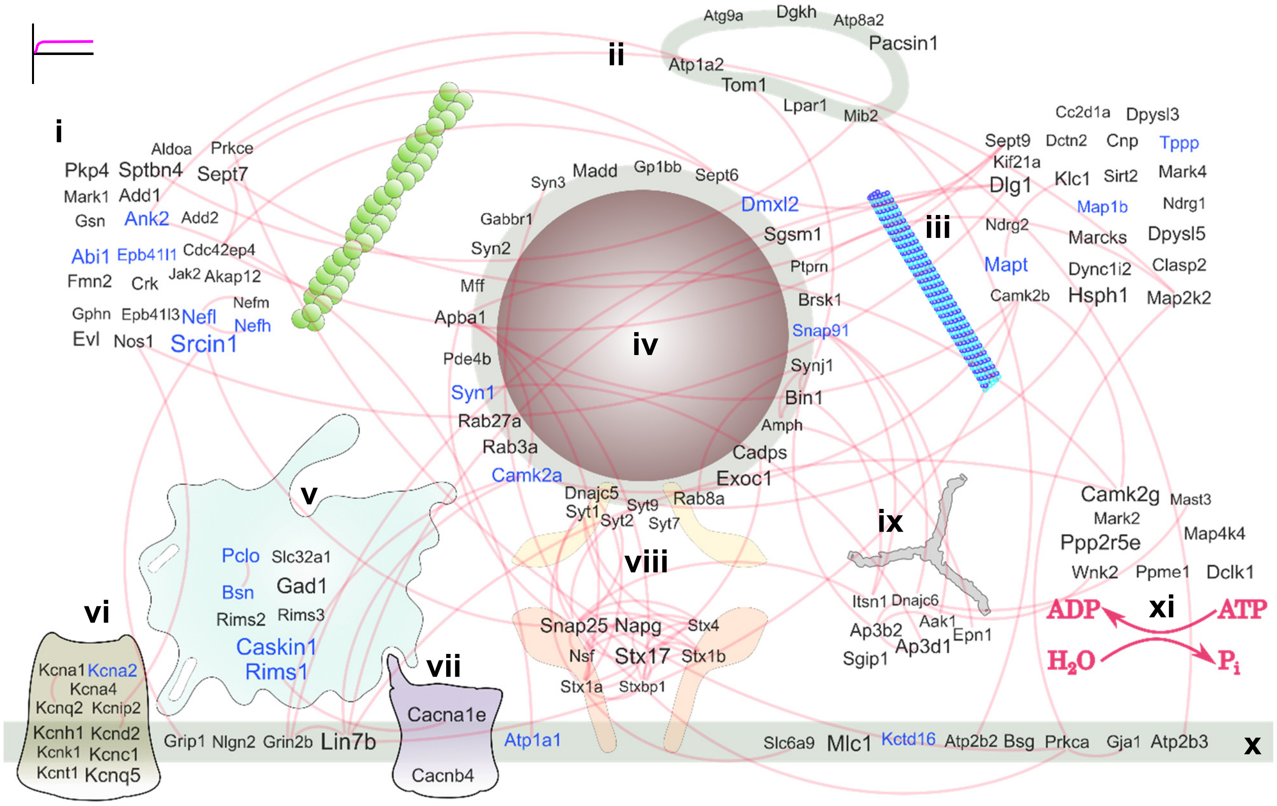

Supplement: S8 Fig — A word cloud visualization of (A) cluster 5 low-magnitude down-regulated phosphorylation and (B) cluster 6 low-magnitude up-regulated phosphorylation, from synaptosomes, using gene names anchored to cellular component ontology (subcellular localization). The size of the gene name was scaled to the average poststimulus log2(stimulated intensity/control intensity) value after both 20 mM and 76 mM KCl stimulation. Localizations: (i) cytoskeleton and actin cytoskeleton, (ii) endosome, (iii) microtubule and microtubule organizing centre, (iv) synaptic vesicle, (v) active zone scaffold, (vi) voltage-gated potassium channel, (vii) voltage-gated calcium channel, (viii) SNARE complex, (ix) clathrin-coated pit, and (x) presynaptic membrane and membrane raft. (xi) Protein kinases and protein phosphatases, including regulatory subunits, with no specific localization are also shown. Gene names in blue letters have three or more responding phosphorylation sites. Experimentally verified protein interactions (STRING) are shown as red edges, i.e., connecting lines. The data are the result of six independent experiments for each stimulation condition (20 mM and 76 mM KCl). SNARE, soluble N-ethylmaleimide-sensitive factor attachment protein receptor. (PDF) [file pbio.3000170.s008.pdf]
